# The late-stage steps of *Burkholderia cenocepacia* protein O-linked glycan biosynthesis are conditionally essential

**DOI:** 10.1016/j.jbc.2025.108515

**Published:** 2025-04-24

**Authors:** Leila Jebeli, Taylor A. McDaniels, Duncan T.T. Ho, Hamza Tahir, Nicholas L. Kai-Ming, Molli Mcgaw, Kristian I. Karlic, Jessica M. Lewis, Nichollas E. Scott

**Affiliations:** Department of Microbiology and Immunology, University of Melbourne at the Peter Doherty Institute for Infection and Immunity, Melbourne, Australia

**Keywords:** glycosylation, *Burkholderia cenocepacia*, B*urkholderia*, post-translational modifications, proteomics, glycoproteomics

## Abstract

Periplasmic *O*-linked protein glycosylation is a highly conserved process observed across the *Burkholderia* genus. Within Burkholderia, protein glycosylation requires the five-gene cluster known as the *O*-glycosylation cluster (OGC, *ogcXABEI*), which facilitates the construction of the *O*-linked trisaccharide attached to periplasmic proteins. Previous studies have reported conflicting results regarding the essentiality of *ogcA*, predicted to be responsible for the addition of the final carbohydrate of the *O*-linked trisaccharide, and *ogcX,* the putative *O*-linked glycan flippase. Within this work, we aimed to dissect the impact of the loss of *ogcA* and *ogcX* on *Burkholderia cenocepacia* viability. We demonstrate that the loss of either *ogcA* or *ogcX* is detrimental if glycosylation is initiated, leading to marked phenotypic effects. Proteomic analysis supports that the loss of *ogcA*/*ogcX* both blocks glycosylation and drives pleotropic effects in the membrane proteome, resulting in the loss of membrane integrity. Consistent with this, strains lacking *ogcA* and *ogcX* exhibit increased sensitivity to membrane stressors, including antibiotics, and demonstrate marked changes in membrane permeability. These effects are consistent with the fouling of the undecaprenyl pool due to dead-end *O*-linked glycan intermediates, and consistent with this, we show that modulation of the undecaprenyl pool through the overexpression of undecaprenyl pyrophosphate synthase (UppS) or the OGC flippase (OgcX) restores viability, while expression of early-stage OGC biosynthesis genes (*ogcI* and *ogcB*) reduces *B. cenocepacia* viability. These findings demonstrate that disrupting *O*-linked glycan biosynthesis or transport appears to dramatically impact *B. cenocepacia* viability, supporting the assignment of *ogcA* and *ogcX* as conditionally essential.

Across bacterial species many surface polysaccharides are synthesized utilizing the essential polyisoprenoid lipid undecaprenyl phosphate (Und-P) (1, 2). Functioning as a chemical carrier, Und-P provides an assembly point for the stepwise construction of diverse lipid-linked oligosaccharide units at the inner face of the cytoplasmic membrane within Gram-negative and Gram-positive species (1, 2). By providing a tethering point to enable glycan elongation, Und-P allows the construction of a range of glycans, which upon translocation across the cytoplasmic membrane can be integrated into glycoconjugates including peptidoglycan, cell wall teichoic acids, O-antigens, capsules, and exopolysaccharides (3, 4). While Und-P and its phosphorylated form undecaprenyl pyrophosphate (Und-PP) are important for the construction of surface carbohydrates, these molecules account for less than 1% of the total lipid pool (5), resulting in competition for available Und-P and the need for rapid flux of oligosaccharide units through the Und-P/Und-PP cycle (6, 7). Under normal physiological conditions, *de novo* synthesis and recycling of Und-P/Und-PP are able to meet physiological demands (6, 7), yet it is increasingly recognized that genetic manipulations that result in Und-PP-glycan intermediates that sequester the Und-P/Und-PP pool can dramatically impact cellular viability, morphology and growth (6, 7, 8, 9, 10, 11). Understanding Und-PP-glycan intermediates with the capacity to foul the Und-P/Und-PP pool has the potential to not only aid antimicrobial development (12, 13) but also explain the mechanisms underpinning the homogeneity observed within the glycan structures of glycoconjugates (14, 15, 16).

Ensuring only glycans possessing specific structural configurations, herein referred to as glycan fidelity, are translocated across the cytoplasmic membrane ensures homogeneity within oligosaccharide units constructed utilizing the Und-P/Und-PP pool (3, 4). For many glycoconjugates generated using Und-PP-linked oligosaccharides, the addition of specific glycan components is a prerequisite for effective translocation across the cytoplasmic membrane by the actions of dedicated translocases known as flippases (17, 18, 19). Of the known flippases the Wzx enzymes represent a highly diverse family of enzymes (19, 20) responsible for the translocation of a range of carbohydrates, including O-antigens (21), capsules (22, 23), exopolysaccharides (24), and the enterobacterial common antigen (ECA) (10). While Wzx flippases were initially thought to possess limited capacity for the recognition of glycan structures (25, 26), it has become clear multiple features of their cognate glycans, such as specific branching or terminal sugars in addition to the initiating sugar influence translocation efficiency when Wzx enzymes are expressed under native-like conditions (3, 15, 27, 28). This specificity results in discrete Wzx flippases being observed genetically linked to their cognate glycan clusters (15, 22, 23, 27) with Wzx enzymes suggested to function as quality control mechanisms (16), ensuring only “complete” glycans are effectively translocated across the cytoplasmic membrane. The specificity for complete glycans by Wzx flippases in concert with the extension of glycans beyond key points commits Und-PP-linked oligosaccharides to the generation of specific glycans (11, 25, 29, 30, 31, 32). In cases where Und-PP-linked intermediates are unable to be completed, this drives detrimental phenotypes due to the sequestration of the Und-P/Und-PP pool, including growth defects, enhanced sensitivity to membrane stresses, and alterations in cellular morphology (6, 7, 11). Importantly, the profound effects of Und-P/Und-PP sequestration are known to rapidly lead to the emergence of suppressor mutations, which complicates the analysis of Und-P/Und-PP glycan biosynthesis pathways by obfuscating the role of specific glycan biosynthesis genes (9, 33, 34). While these potential confounding effects are now well appreciated for the O-antigens (6, 11), capsule (35), and ECA (7), this phenomenon has not been explored within glycans used for other glycoconjugates such as protein glycosylation.

Protein glycosylation systems are widespread across bacterial species (36, 37) including members of the *Campylobacter* (38, 39), *Neisseria* (40, 41, 42, 43, 44), and *Burkholderia* (45, 46, 47) genera. To date, several Und-P/Und-PP dependent glycosylation systems have been described in Gram-negative species, which utilize the Und-P lipid pool to enable periplasmic glycosylation leading to glycosylation of tens to hundreds of proteins (37, 48, 49, 50). While similarities within the biosynthetic clusters used for protein glycosylation systems and lipo-oligosaccharide biosynthetic clusters have been previously highlighted (36, 37), a notable difference within well-characterized glycosylation systems is the use of ABC transporter-based flippases (36, 37) as opposed to Wzx flippases. ABC transporter-based flippases, such as PglK (also known as WlaB (51)) within *Campylobacter* sp (52, 53). and PglF within *Neisseria* sp (54, 55, 56). are dispensable for glycosylation (53, 56) as well as possess relaxed glycan specificities as demonstrated using heterogenous expression studies for PglK (53) and the observation of glycan microheterogeneity within *Neisseria* (57, 58). The tolerance of these glycosylation pathways to both glycan alterations, as well as the loss of their flippases, is in stark contrast to the strict specificity and essentiality seen within other Und-P/Und-PP dependent systems, yet not all Und-P/Und-PP dependent protein glycosylation systems utilize ABC transporter flippases.

Within *Burkholderia* species, periplasmic glycosylation is highly conserved (45, 46, 47), leading to nearly exclusive modification of serine residues (45) with a trisaccharide corresponding to β-Gal-(1,3)–α-GalNAc-(1,3)–β-GalNAc (47). *Burkholderia O*-linked glycan assembly is mediated by a single gene cluster known as the *O*-linked Glycosylation Cluster (*OGC*), consisting of five genes (*ogcX*, *A*, *B*, *E,* and *I* corresponding to BCAL3114 to BCAL3118 within *Burkholderia cenocepacia,* respectively) (47). Our current model of glycan assembly (Fig. 1*A*) supports OgcE acts as an UDP-Glc/UDP-GlcNAc epimerase, OgcI as the initiating glycotransferase, OgcB and OgcA as the glycosyltransferases required for the second GalNAc and final Gal residues, respectively, and with the putative Wzx flippase OgcX responsible for translocating the glycan across the cytoplasmic membrane (47), where it can then be transferred to glycoproteins by the oligosaccharyltransferase PglL (BCAL0960) (47, 59). Previous studies have provided experimental evidence for the function of OgcB, OgcE, and OgcI (47), yet contradictory results have been obtained for the enzymes OgcA and OgcX. To date, strains lacking *ogcA* have not been generated within *B. cenocepacia,* yet loss of *ogcX* has been previously reported to be tolerated (47). Saturation transposon studies from several *Burkholderia* species (60, 61, 62) have revealed variability in the ability to disrupt *ogcA* and *ogcX,* with some studies concluding these enzymes are essential (62) while others suggest that at least for *ogcA* disruptions within this gene may be tolerated (61). These observations support that, unlike the glycosylation systems of the *Campylobacter* and *Neisseria* genera, disruptions in glycan biosynthesis steps may not be tolerated within *Burkholderia.* These contradictory results support that further assessments are required to dissect the impact of loss of *ogcA* and *ogcX* on viability and protein glycosylation.

**Figure 1:**
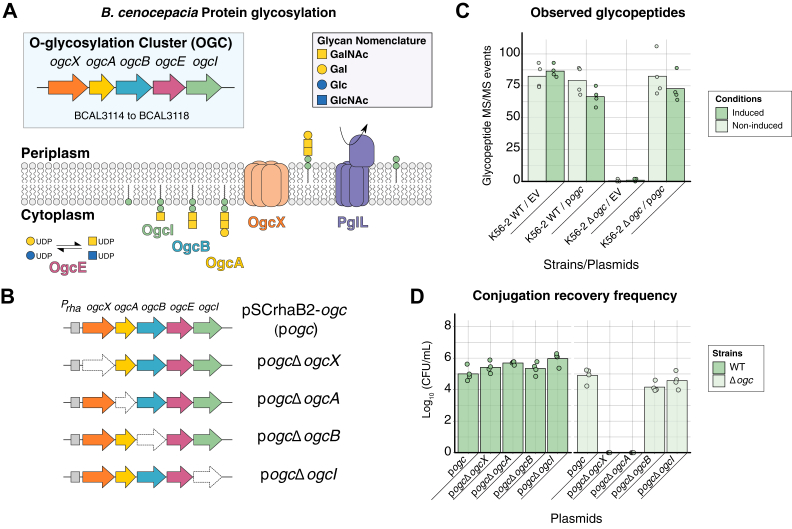
**Loss of *ogc*X and *ogc*A impacts viability of *B. cenocepacia* Δ*ogc*.***A*, diagram of the *O*-glycosylation biosynthesis pathway in *Burkholderia* species and the genetic organization of the *ogc* cluster (BCAL3114–BCAL3118 within *B. cenocepacia* J2315) (47). *O*-glycosylation biosynthesis involves the stepwise construction of a conserved trisaccharide composed of Gal-GalNAc_2_ by the glycosyltransferases, OgcI, OgcB and OgcA, with the nucleotide-sugar donors UDP-GalNAc and UDP-Gal generated by the epimerase OgcE. The resulting trisaccharide is then thought to be translocated across the inner membrane by the putative flippase, OgcX. Once in the periplasm, the glycans can be transferred to proteins by the oligosaccharyltransferase, PglL. Monosaccharides have been annotated according to the Symbol Nomenclature for Glycans (SNFG) (119). *B*, graphic representation of p*ogc* and variants lacking *ogc*X, *ogc*A, *ogc*B and *ogc*I. *C*, quantification of glycopeptide and protein levels encoded by the *ogc* cluster identified from whole cell proteomic analysis of *B. cenocepacia* WT and Δ*ogc* containing either the empty vector pSCrhaB2 (EV) or pSCrhaB2-*ogc* (p*ogc*) with and without induction with 1% rhamnose (n = 4). *D*, conjugation of p*ogc* derivatives into *B. cenocepacia* K56-2 WT and Δ*ogc* reveals differences in recovery rates of p*ogc*Δ*ogc*X and p*ogc*Δ*ogc*A within *B. cenocepacia* Δ*ogc* (n = 4).

Within this work, we sought to assess the requirement of *ogcA* and *ogcX* for protein glycosylation and the impact of the loss of these *ogc* components on *B. cenocepacia.* Utilizing complementary genetic approaches, including *ogc* complementation within strains lacking the *ogc* cluster (47, 63) as well as glycosylation inducible strains containing the initiating transferase, *ogcI,* under rhamnose control (64), we assess the proteomic and physiological impacts of the loss of *ogcA* and *ogcX.* We demonstrate that in the absence of glycosylation initiation, *ogcA* and *ogcX* are dispensable, yet upon glycosylation initiation, loss of these genes leads to growth defects, widespread proteomic impacts, changes in the membrane integrity, and the absence of observable glycosylation. While these changes can be complemented and compensated by the modulation of enzymes associated with *de novo* synthesis of Und-P as well as the overexpression of the flippase OgcX, we observe that the modulation of early steps in the *O*-linked glycan biosynthesis pathways can also drive changes in viability. These data support that the construction of the O-linked glycan in *B. cenocepacia* is finely tuned, with *ogc*A and *ogc*X being conditionally essential, herein defined as these genes being required for viability and wild-type levels of growth, yet dispensable if preceding steps in *O*-linked glycan biosynthesis, including glycan initiation with OgcI or elongation by OgcB, are disrupted.

## Results

### Loss of *ogcA* and *ogcX* impacts the viability of *B. cenocepacia*

To dissect the impact of individual *ogc* genes on viability, we established a rhamnose-inducible vector pSCrhaB2 (64) containing the *ogc* cluster (pSCrhaB2-*ogc*) herein referred to as p*ogc* (Fig. 1*B*), and assessed its ability to restore glycosylation within *B. cenocepacia* Δ*ogc* (47). Using mass spectrometry-based proteomics, we confirmed the restoration of glycosylation regardless of rhamnose induction and the absence of glycosylation within Δ*ogc* containing pSCrhaB2 empty vector (EV) (Fig. 1*C*, Tables S6 and S7). With the identification that p*ogc* restored glycosylation, *ogcX* and *ogcA* were removed within p*ogc* to assess the impact on viability with *ogcI*/*ogcB* removed as additional controls (Fig. 1*B*). Introduction of plasmids into *B. cenocepacia* by conjugation revealed that the removal of *ogcA, B, I,* or *X* resulted in comparable recovery of transformants within *B. cenocepacia* WT (Fig. 1*D*). In contrast, for Δ*ogc,* the introduction of p*ogc*Δ*ogcX* or p*ogc*Δ*ogcA* resulted in dramatic reductions in recoverable transformants (Fig. 1*D*), supporting that the loss of either *ogcX* and *ogcA* from p*ogc* impacts viability in the absence of chromosomally encoded *ogcX* and *ogcA*.

### Deleterious effects due to the loss of *ogcX* or *ogcA* are dependent on glycosylation initiation

**Figure 2:**
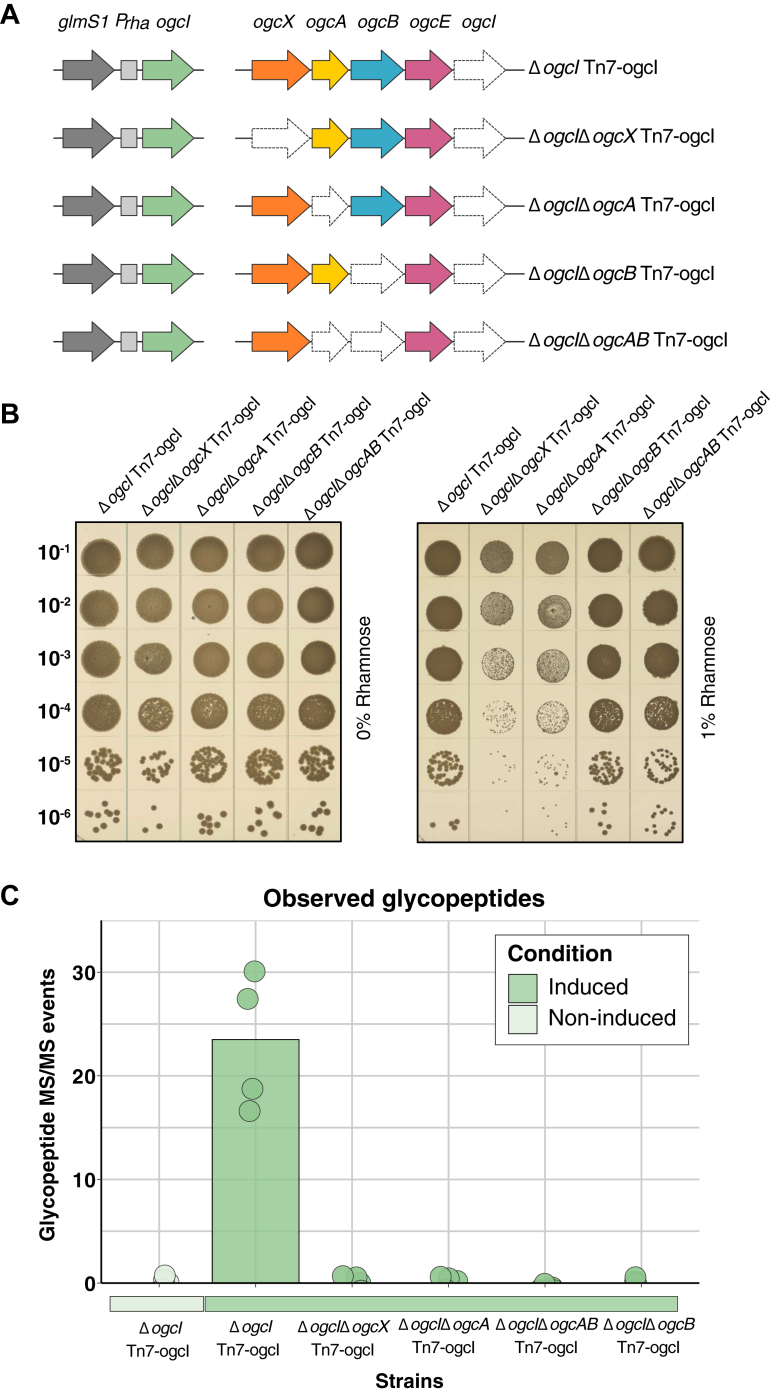
**Loss of *ogcX* or *ogcA* leads to reduced growth when glycosylation is initiated.***A*, graphic representation of strains Δ*ogcX,* Δ*ogcA,* Δ*ogcB and* Δ*ogcAB* generated within the chromosomal inducible background *B. cenocepacia* Δ*ogcI* Tn7-*ogcI*. *B*, spot plate assays assessing growth of strains with and without 1% rhamnose induction. Induction results in reduced colony size of Δ*ogcX* and Δ*ogcA* strains to 10% of the size observed in *B. cenocepacia* Δ*ogcI* Tn7-*ogcI* (n = 4). *C*, quantification of glycopeptides identified from whole-cell proteomic analysis of strains. Glycopeptides were assessed in the absence (*light green*) or presence (*dark green*) of rhamnose induction (n = 4). Induction of *ogcI* expression by 1% rhamnose led to the restoration of glycosylation only in *B. cenocepacia* Δ*ogcI* Tn7-*ogcI* with no glycosylation observed in the Δ*ogcX*, Δ*ogcB*, and Δ*ogcAB* strains.

The absence of effects on viability in response to the loss of the *ogc* (47, 63), yet the requirement of *ogcA*/*ogcX* alone supports that if *O*-linked glycan biosynthesis is allowed to proceed, it is deleterious after the addition of the second GalNAc, mediated by the OgcB (47). If true, we reasoned that controlling the initiation of glycosylation *via* placing OgcI under inducible control should render Δ*ogcA and* Δ*ogcX* viable. To assess this, we removed *ogcI* and chromosomally reintroduced *ogcI* under rhamnose-inducible control within a miniTn7 integrative system (Figs. 2*A*, S1*A*), generating Δ*ogcI* Tn7-*ogcI.* Proteomic and glycoproteomic analysis confirmed the inducible control of *ogcI* as well as the appearance of glycosylation upon the addition of rhamnose in this background (Figs. S1*B* and S2*C*). Following verification of Δ*ogcI* Tn7-*ogcI,* we generated viable mutations in *ogcX and ogcA* (Δ*ogcI*Δ*ogcX* Tn7-*ogcI* and Δ*ogcI*Δ*ogcA* Tn7-*ogcI*) as well as *ogcB and ogcAB (*Δ*ogcI*Δ*ogcB* Tn7-*ogcI* and Δ*ogcI*Δ*ogcAB* Tn7-*ogcI*). Consistent with the requirement of glycosylation initiation to drive deleterious effects, spot plate assays revealed reduced growth of Δ*ogcI*Δ*ogcA* Tn7-*ogcI* and Δ*ogcI*Δ*ogcX* Tn7-*ogcI* strains in the presence of rhamnose, with no impact on growth observed for Δ*ogcI*Δ*ogcAB* Tn7-ogcI*,* Δ*ogcI*Δ*ogcB* Tn7-ogcI, or Δ*ogcI* Tn7-*ogcI* (Fig. 2*B*). Quantification of colony size within strains lacking *ogcX* or *ogcA* revealed a reduction in size to 10% of Δ*ogcI* Tn7-*ogcI*, Δ*ogcI*Δ*ogcB* Tn7-*ogcI* and Δ*ogcI*Δ*ogcAB* Tn7-*ogcI* strains in response to induction (Fig. S2). Importantly, across strains, quantification of glycosylation using glycoproteomics supports that while rhamnose induction led to the restoration of glycosylation within Δ*ogcI* Tn7-*ogcI,* no glycosylation was observable within strains lacking *ogcA, B, X or AB* upon rhamnose induction (Fig. 2*C*, Table S8).

**Figure 3:**
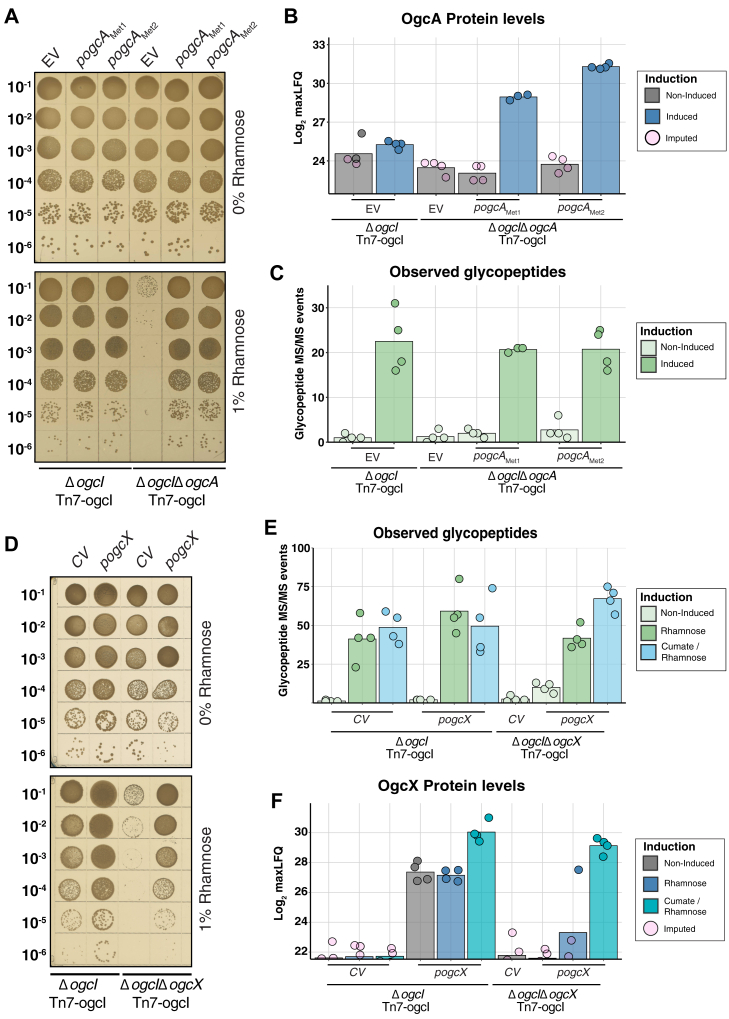
**Complementation of Δ*ogcA* and Δ*ogcX* restores growth and glycosylation.***A*, spot plate assays of *B. cenocepacia* Δ*ogcI* Tn7-*ogcI* and *B. cenocepacia* Δ*ogcI*Δ*ogcA* Tn7-*ogcI* containing pSCrhaB2 (EV), pSCrhaB2-*ogcA*-Met1 (p*ogcA*_Met1_), or pSCrhaB2-*ogcA*-Met2 (p*ogcA*_Met2_) with and without 1% rhamnose induction revealing complementation of Δ*ogcA* with *ogcA* variants restores viability and colony size. *B* and *C*, quantification of glycopeptides and OgcA levels from whole-cell proteomic analysis of *B. cenocepacia* Δ*ogcI* Tn7-*ogcI* and *B. cenocepacia* Δ*ogcI*Δ*ogcA* Tn7-*ogcI* strains carrying EV, *ogcA*_Met1_, or *ogcA*_Met2_. Induction with 1% rhamnose results in the detection of OgcA within *B. cenocepacia* Δ*ogcI*Δ*ogcA* Tn7-*ogcI* and the restoration of glycosylation (n = 4, Δ*ogcI* Δ*ogcA* Tn7-*ogcI ogcA*-Met1 induced group n = 3). *D*, spot plate assays of *B. cenocepacia* Δ*ogcI* Tn7-*ogcI* and *B. cenocepacia* Δ*ogcI*Δ*ogcX* Tn7-*ogcI* carrying the control vector pcumate-sfGFP (CV) and pcumate-*ogcX-his* (p*ogc*X) with and without 1% rhamnose induction revealing complemented Δ*ogcX* restores viability and colony size. *E* and *F*, quantification of glycopeptides and OgcX levels from whole-cell proteomic analysis of *B. cenocepacia* Δ*ogcI* Tn7-*ogcI* and *B. cenocepacia* Δ*ogcI*Δ*ogcX* Tn7-*ogcI* strains carrying sfGFP or *ogcX* plasmids. Induction results in the detection of OgcX within *B. cenocepacia* Δ*ogcI*Δ*ogcX* Tn7-*ogcI* and the restoration of glycosylation (n = 4). Imputed values correspond to missing quantitation events resulting from the low abundance or absence of OgcX or OgcA within biological replicates.

To confirm that the observed growth defects were dependent on *ogcX* and *ogcA*, plasmid-based complementation was performed. For OgcA complementation, two rhamnose-inducible vectors, p*ogcA*_Met1_ and p*ogcA*_Met2_, corresponding to variants with different potential start codons of OgcA (Fig. S3), were generated. Upon induction, both p*ogcA*_Met1_ and p*ogcA*_Met2_ in Δ*ogcI*Δ*ogcA* Tn7-ogcI restored colony size to that of the parental strain Δ*ogcI* Tn7-ogcI compared to the empty vector (EV, Fig. 3*A*). Proteomic and glycoproteomic analyses confirmed the restoration of OgcA as well as glycosylation in a p*ogcA*_Met1_/p*ogcA*_Met2_ and rhamnose-dependent manner (Fig. 3, *B* and *C*, Tables S9 and S10). For OgcX complementation, a cumate-inducible vector p*ogcX* was generated. Introduction of p*ogcX* into Δ*ogcI*Δ*ogcX* Tn7-ogcI restored colony size upon rhamnose induction, with p*ogcX* relieving the deleterious effects of glycosylation initiation compared to the control vector (CV, Fig. 3*D*). Glycoproteomic analysis confirmed the restoration of glycosylation in Δ*ogcI*Δ*ogcX* Tn7-*ogcI* containing p*ogc*X in a rhamnose-dependent manner, with the addition of both cumate and rhamnose observed to enhance glycosylation (Fig. 3*E*, Table S11). Proteomic analysis confirmed that OgcX production was enhanced upon cumate induction. (Fig. 3*F*, Table S12). Consistent with the loss of viability observed using spot assays, it should be noted that for Δ*ogcI*Δ*ogcA* Tn7-*ogcI* and Δ*ogcI*Δ*ogcX* Tn7-*ogcI* carrying EV or CV, respectively, even modest levels of glycosylation initiation led to the loss of viability (Fig. S4). These findings support that the loss of *ogc*A and *ogc*X drives the deleterious effects observed upon glycosylation initiation in *B. cenocepacia*, and the restoration of these proteins restores glycosylation.

### Proteomic analysis reveals loss of *ogcX* or *ogcA* drives alterations within the membrane proteome

**Figure 4:**
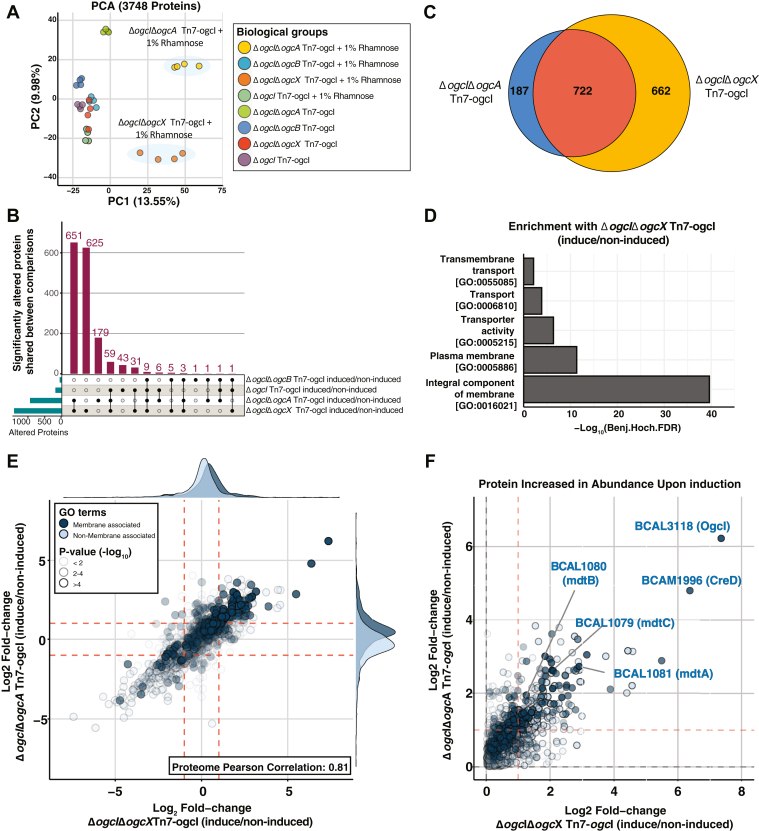
**Proteomic analysis of strains in response to glycosylation initiation.** DIA proteomic analysis of Δ*ogcI* Tn7-ogcI, Δ*ogcI*Δ*ogcA* Tn7-*ogcI*, Δ*ogcI*Δ*ogcB* Tn7-*ogcI*, and Δ*ogcI*Δ*ogcX* Tn7-*ogcI* strains with and without rhamnose induction (n = 4). *A*, principal component analysis (PCA) of Δ*ogcI* Tn7-*ogcI* strains under induced and non-induced conditions. Biological groups are observed to form distinct clusters, with Δ*ogcI*Δ*ogcA* Tn7-*ogcI* and Δ*ogcI*Δ*ogcX* Tn7-*ogcI* strains separating along PC1 upon induction. *B* and *C*, upset plot and Venn diagram of proteomic alterations, defined as >two-fold-change and −log_10_(*p*-value) > 2 observed across strains, reveal that induction results in hundreds of protein alterations within Δ*ogcI*Δ*ogcA* Tn7-*ogcI* and Δ*ogcI*Δ*ogcX* Tn7-*ogcI* strains compared to Δ*ogcI*Δ*ogcB* Tn7-*ogcI* or Δ*ogcI* Tn7-ogcI. *D*, enrichment analysis of GO terms associated with the altered proteins in Δ*ogcI*Δ*ogcX* Tn7-*ogcI* strain reveals an over-representation of proteomic changes in membrane-associated protein classes. *E*, 2D scatter plots comparing proteome changes observed within Δ*ogcI*Δ*ogcA* Tn7-*ogcI* and Δ*ogcI*Δ*ogcX* Tn7-*ogcI* in response to glycosylation initiation. Proteins associated with membrane GO-terms are color-coded dark blue with the opacity corresponding to the average *p*-values observed across both comparisons. *F*, zoomed in 2D scatter plots of proteins observed to increase upon induction within Δ*ogcI*Δ*ogcA* Tn7-*ogcI* and Δ*ogcI*Δ*ogcX* Tn7-*ogcI* with membrane proteins of note highlighted.

To improve the depth and reproducibility of our proteomic analysis, we employed Data-Independent Acquisition (DIA) to compare the response to rhamnose-induction within Δ*ogcI* Tn7-*ogc*I, Δ*ogc*IΔ*ogc*A Tn7-*ogc*I, Δ*ogc*IΔ*ogc*B Tn7-ogcI, and Δ*ogc*IΔ*ogc*X Tn7-*ogcI*. Using DIA proteomics, a total of 3748 proteins were identified, including all members of the OGC, confirming the expected loss of OgcA, OgcB, OgcI, and OgcX in the respective strains/induction states (Figs. S5 and S6*A*, Table S13). Principal component analysis revealed distinct clustering of strains with induced Δ*ogcI*Δ*ogcA* Tn7-*ogcI* and Δ*ogcI*Δ*ogcX* Tn7-*ogc*I separating away from all other groups (Fig. 4*A*). Examination of the protein alterations (defined as >two-fold difference, −log10(*p*-value) > 2) revealed a total of 1384 and 909 alterations observed within strains lacking *ogcX* and *ogcA* upon induction, respectively (Fig. 4*B*), with 722 proteins altered in both strains representing a significant co-enrichment of protein alterations (Fig. 4*C*, Fisher exact test Benjamini-Hochberg corrected *p*-value = 8.20∗10^−204^, Table S14). In contrast, within Δ*ogc*I Tn7-*ogc*I and Δ*ogcI*Δ*ogcB* Tn7-*ogcI,* only 150 and 21 protein alterations were observed, respectively, with these changes having little overlap with those observed in strains lacking ogcX and ogcA (Fig. 4*B*). The low overlap in proteomic changes compared to Δ*ogc*I Tn7-ogcI, where induction restores glycosylation, supports that the changes observed with Δ*ogc*X and Δ*ogc*A are distinct from those caused by the loss of glycosylation alone.

To assess the functional associations within the induced Δ*ogc*X and Δ*ogc*A proteomes, enrichment analysis was undertaken. Consistent with the changes observed in Δ*ogc*X and Δ*ogc*A being incongruent with the impact observed from the loss of glycosylation alone, only a modest overlap within the proteomic effects previously documented in glycosylation-null strains (63) was observed (Fig. S7, Table S14). Examination of the Gene Ontology terms associated with protein changes reveals an over-representation of membrane-associated protein classes within Δ*ogc*IΔ*ogc*X Tn7-*ogc*I (Fig. 4*D*, Table S14), with similar enrichment patterns observed within Δ*ogc*IΔ*ogcA* Tn7-*ogc*I (Table S14). Consistent with these enrichments, we find highly correlated alterations within the membrane proteome of Δ*ogc*X and Δ*ogc*A strains upon induction (Figs. 4*E* and S6*B*). Examination of the elevated membrane proteins reveal proteins with putative functions linked to maintaining envelope integrity such as BCAM1996 (a homolog of the cell envelope integrity protein CreD (65)) as well as transporters known to be induced during membrane stress responses including MdtABC (66, 67) (BCAL1079-BCAL1081) (Fig. 4*F*). Combined, these findings support that the loss of *ogc*X or *ogc*A leads to widespread changes in the *B. cenocepacia* membrane proteome that are unique to those seen from the loss of glycosylation.

### Loss of *ogcA* and *ogcX* impacts the membrane integrity and permeability of *B. cenocepacia*

**Figure 5:**
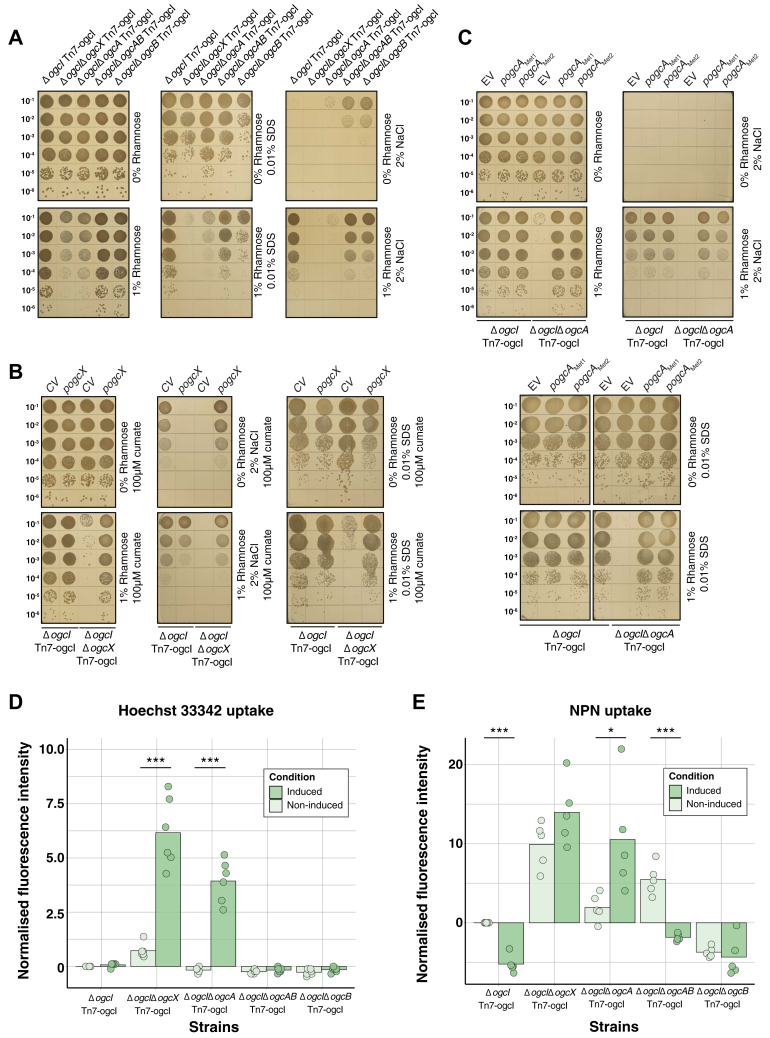
**Loss of *ogcA* and *ogcX* impacts the membrane integrity of *B. cenocepacia*.***A*, spot plate assays of strains in response to membrane (0.01% SDS) and osmotic (2% NaCl) stressors with and without 1% rhamnose induction (n = 4). The deletion of ogcX and *ogcA* increases sensitivity to 0.01% SDS when glycosylation is initiated, while both mutants appear sensitive to osmotic stress (2% NaCl) regardless of induction. *B* and *C*, spot plate assays of complemented Δ*ogc*IΔ*ogc*A Tn7-*ogc*I and Δ*ogc*IΔ*ogc*X Tn7-*ogc*I strains in response to membrane (0.01% SDS) and osmotic (2% NaCl) stress with and without 1% rhamnose induction demonstrating the restoration of growth within complemented strains (n = 4). The empty vector plasmid pSCrhaB2 (EV) and control vector pCumate-sfGFP (CV) correspond to negative control plasmids for p*ogcA*_Met1_/p*ogcA*_Met2_ and p*ogcX*, respectively. *D* and *E*, hoechst 33,342 and NPN uptake assays of strains reveal loss of *ogcA* and *ogcX* leads to increased fluorescence upon induction, supporting enhanced membrane permeability (n = 6 for Hoechst 33,342, n = 5 for NPN). The fluorescence intensities have been normalised against bacterial viability counts (Fig. S9).

Given the dramatic membrane proteomic alterations in response to the loss of *ogcX* and *ogcA* upon induction, this suggests the loss of membrane integrity as noted during the inhibition of several Wzx-dependent glycan pathways (4, 6, 7, 34, 68). To assess membrane integrity, we assayed the impact of membrane stress (0.01% SDS) using spot plate assays revealing increased sensitivity of Δ*ogcI*Δ*ogcA* Tn7-*ogcI* and Δ*ogcI*Δ*ogcX* Tn7-*ogcI* upon glycosylation initiation compared to Δ*ogcI* Tn7-*ogcI,* Δ*ogcI*Δ*ogcB* Tn7-*ogcI or* Δ*ogcI*Δ*ogcAB* Tn7-*ogcI* strains (Fig. 5*A*). In contrast, osmotic stress (2% NaCl) spot plate assays revealed adverse impacts on all strains in the absence of induction, yet only *ogcX* and *ogcA* deletion strains failed to show improved viability following glycosylation initiation (Fig. 5*A*). Microtiter plate-based growth assays recapitulated these observations with the strains lacking *ogcX* and *ogcA* demonstrating alterations in growth kinetics in the presence of 0.01% SDS or 2% NaCl (Fig. S8). Consistent with the loss of viability being driven by the absence of *ogcX* and *ogcA* complementation ameliorated the growth defects in the presence of 0.01% SDS or 2% NaCl (Fig. 5, *B* and *C*). These observations support the loss of membrane integrity within Δ*ogcX* and Δ*ogcA* backgrounds yet to directly assess membrane permeability in response to glycosylation initiation, we undertook Hoechst 33,342 and N-phenyl-1-naphthylamine (NPN) fluorescent-based permeability assays. Utilizing Hoechst 33,342, a fluorescent DNA binding dye, we evaluated DNA binding revealing enhanced fluorescence within Δ*ogcX* and Δ*ogcA* backgrounds upon glycosylation initiation, yet minimal alterations in Δ*ogcI* Tn7-*ogcI,* Δ*ogcI*Δ*ogcB* Tn7-*ogcI or* Δ*ogcI*Δ*ogcAB* Tn7-*ogcI* (Fig. 5*D*). Similarly, NPN uptake assays, which monitor dye partitioning into membranes (69), revealed increased fluorescence in Δ*ogcI*Δ*ogcX* Tn7-*ogcI* and Δ*ogcI*Δ*ogcA* Tn7-*ogcI* upon induction, supporting increased membrane permeability (Figs 5*E*, S9) with complementation of Δ*ogcI*Δ*ogcX* Tn7-*ogcI* and Δ*ogcI*Δ*ogcA* Tn7-*ogcI* reversing membrane permeability (Fig. S10). Combined, these findings support the absence of *ogcX* and *ogcA* upon glycosylation initiation compromises the *B. cenocepacia* envelope, leading to sensitization to stressors and membrane permeability.

### Loss of *ogcA* and *ogcX* enhances sensitivity of *B. cenocepacia* to antimicrobials

**Figure 6:**
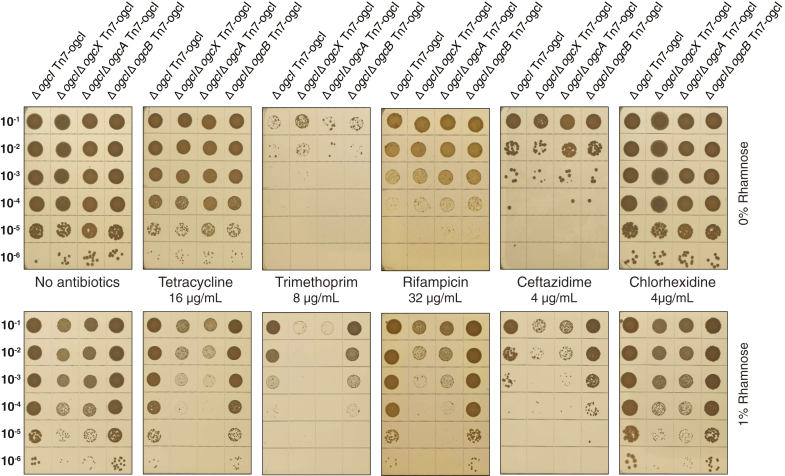
**Loss of *ogcA* and *ogcX* sensitises *B. cenocepacia* to antimicrobials.** Spot plate assays of *B. cenocepacia* strains, including Δ*ogcI* Tn7-*ogcI*, Δ*ogcI*Δ*ogcX* Tn7-*ogcI*, Δ*ogcI*Δ*ogcA* Tn7-*ogcI*, and Δ*ogcI*Δ*ogcB* Tn7-*ogcI* on CAMHA containing various antibiotics, with or without induction with 1% rhamnose. Rhamnose-induced glycosylation resulted in increased susceptibility to tetracycline, rifampicin, trimethoprim and ceftazidime in strains lacking *ogcA* and *ogcX*. Data representative of four biological replicates per strain per antimicrobial.

The observations that the loss of *ogcX* and *ogcA* leads to widespread impacts on the *B. cenocepacia* membrane support that these effects could be exploited to potentiate antimicrobial agents. In line with this, recent transposon studies have suggested that mutations within the *ogc* alter susceptibility to β-lactam antibiotics; however, validation of these effects using CRISPRi resulted in only modest recapitulation of sensitization (61). Given the loss of viability observed under plasmid selection conditions (Figs. 3, *A* and *D*, and S4) compared to the reduced growth observed in the absence of plasmid selection (Figs. 2*B* and S8), this supports that even when possessing a trimethoprim-resistant marker, strains lacking *ogcX* and *ogcA* demonstrate sensitivity to trimethoprim upon glycosylation initiation. Thus, we assessed the impact of antimicrobial agents, including trimethoprim, tetracycline, ceftazidime, chlorhexidine, and rifampicin, on glycosylation inducible strains. Utilizing broth dilution assays, changes in antimicrobial sensitivity were observed in strains lacking *ogcX* and *ogcA* upon induction, yet were modest in magnitude, corresponding to a 1- to 2-fold decrease in the minimal inhibitory concentrations (MIC) of trimethoprim and ceftazidime (Fig. S11 and Table S5). To improve the detection of alterations in antimicrobial sensitivity, we assessed the impact of antimicrobial agents at or below the broth dilution-defined MICs using spot assays. Utilizing this approach, upon induction, strains lacking *ogcX* and *ogcA* were observed to show enhanced sensitivity to tetracycline, rifampicin, trimethoprim, and ceftazidime compared to Δ*ogcI*Δ*ogcB* Tn7-*ogcI* and Δ*ogcI* Tn7-*ogcI* (Fig. 6). For trimethoprim and ceftazidime, all strains showed reduced growth without induction, yet the growth of Δ*ogcI*Δ*ogcB* Tn7-*ogcI* and Δ*ogcI* Tn7-*ogcI* was improved upon induction (Fig. 6). These findings support the loss of *OGC* genes sensitize *B. cenocepacia* to several antimicrobial agents, with these impacts most pronounced for strains lacking *ogcX* or *ogcA* upon initiation of glycosylation.

### Expression of UppS and OgcX improves the viability of *B. cenocepacia* in response to *ogc* biosynthesis blockages

**Figure 7:**
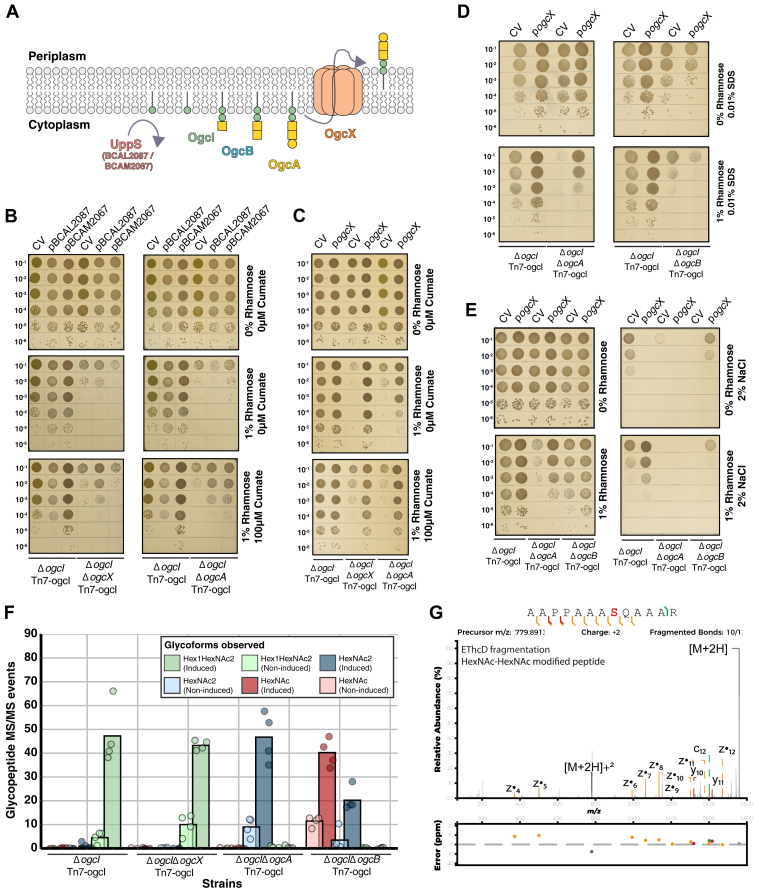
**Overexpression of UppS or OgcX modulates *ogc* associated defects in *B. cenocepacia*.***A*, proposed enzymes responsible for the *de novo* synthesis of the undecaprenyl pool and steps within the *ogc* biosynthesis. Within *B. cenocepacia*, two putative undecaprenyl pyrophosphate synthases (UppS) are assigned corresponding to BCAL2087 and BCAM2067, while OgcI is responsible for *ogc* initiation and OgcB the addition of the second GalNAc (47). The functions of OgcA, as the glycotransferase responsible for the addition of the final Gal and OgcX, as the flippase responsible for glycan translocation have only been inferred to date. *B*, spot plate assays of the *B. cenocepacia* Δ*ogcI* Tn7-*ogcI*, Δ*ogcI*Δ*ogcX* Tn7-*ogcI* and Δ*ogcI*Δ*ogcA* Tn7-*ogcI* containing the control vector pcumate-sfGFP (CV), pcumate-BCAL2087 or pcumate-BCAM2067 reveal the initiation of glycosylation reduces viability while induction of putative UppS partially restores the defect observed within Δ*ogcA* and Δ*ogcX* strains (n = 4). *C*, spot plate assays of the *B. cenocepacia* Δ*ogcI* Tn7-*ogcI*, Δ*ogcI*Δ*ogcX* Tn7-*ogcI* and Δ*ogcI*Δ*ogcA* Tn7-*ogcI* containing the control vector (CV) and pcumate-ogcX (*pogcX*) demonstrating the complementation of Δ*ogcI*Δ*ogcA* Tn7-*ogcI* with *pogcX* restores growth (n = 4). *D and E*, spot plate assays of the *B. cenocepacia* Δ*ogcI* Tn7-*ogcI*, Δ*ogcI*Δ*ogcA* Tn7-*ogcI* and Δ*ogcI*Δ*ogcB* Tn7-*ogcI* containing the control vector (CV) and *pogcX* in response to membrane (0.01% SDS) and osmotic (2% NaCl) stresses with glycosylation initiation demonstrating that p*ogcX* restores resistance to stress in Δ*ogcI*Δ*ogcA* Tn7-*ogcI* yet does not suppress the defect observed in Δ*ogcI*Δ*ogcB* Tn7-*ogcI* (n = 4). *F*, quantification of glycopeptides from whole-cell proteomic analysis of *B. cenocepacia* Tn7-*ogcI* strains carrying p*ogcX*. Induction results in the detection of glycosylation with varying glycoforms observed across strains (n = 4). *G*, EThcD fragmentation of the HexNAc_2_ modified peptide ^397^AAPPAAASQAAAR^409^ (BCAL1674 Uniprot: B4E8U6) confirming the localization of the HexNAc_2_ glycosylation event residue S^404^.

As the deletion of *ogcA* and *ogcX* leads to deleterious effects upon glycosylation initiation, we sought to further support the role of Und-P/Und- PP sequestration in driving these effects. We reasoned that modulating Und-P precursor levels or enhancing glycan intermediate translocation would counteract Und-P/Und-PP sequestration. To probe this, we overexpressed undecaprenyl pyrophosphate synthase (UppS), the *de novo* synthase responsible for generating Und-P, which has been previously shown to relieve Und-P sequestration (Fig. 7*A*) (7, 70). Utilizing cumate-inducible vectors containing the two putative annotated UppS enzymes of *B. cenocepacia* K56-2*,* BCAL2087 and BCAM2067 within the vectors p*BCAL2087* and p*BCAM2067* respectively, we assessed the impact of UppS overexpression in the absence of *ogcA* and *ogcX* with glycosylation initiation. Within Δ*ogcI*Δ*ogcA* Tn7-*ogcI* and Δ*ogcI*Δ*ogcX* Tn7-*ogcI*, the induction of p*BCAL2087* partially alleviated reductions observed within Δ*ogcA* and Δ*ogcX*, while p*BCAM2067* only restored viability within Δ*ogcI*Δ*ogcA* Tn7-*ogcI* (Fig. 7*B*). These observations support the partial repression of growth defects upon induction of UppS. To assess if enhancing glycan translocation restores growth, we introduced *pogcX* into Δ*ogcI*Δ*ogcA* Tn7-*ogcI* improving growth upon the initiation of glycosylation (Fig. 7*C*). Membrane integrity spot assays reveal the amelioration of growth defects within Δ*ogcI*Δ*ogcA* Tn7-*ogcI* upon complementation with *pogcX* compared to the control vector (Fig. 7, *D*/E). While *pogcX* improved resistance to membrane stresses in Δ*ogcI*Δ*ogcA* Tn7-*ogcI* in response to 0.01% SDS (Fig. 7*D*) we observed reduced viability within Δ*ogcI*Δ*ogcB* Tn7-*ogcI* in response to salt and SDS stress compared to the control vector (Fig. 7, *D*/E). To confirm the overexpression of OgcX allowed the translocation of incomplete *O*-linked glycans we undertook glycoproteomic analysis confirming both enhanced glycosylation within inducible strains and the appearance of truncated glycans within Δ*ogcI*Δ*ogcA* Tn7-*ogcI* and Δ*ogcI*Δ*ogcB* Tn7-*ogcI* (Fig. 7*F*, Tables S15 and S16). Manual inspection of glycopeptide assignments within Δ*ogcI*Δ*ogcA* Tn7-*ogcI* confirms the identity of these glycosylation events as corresponding to the modification of peptides with HexNAc_2_ glycans (Fig. 7*G*). While glycopeptide analysis suggested the presence of both HexNAc_2_ and HexNAc modified peptides within Δ*ogcI*Δ*ogcB* Tn7-*ogcI* (Fig. 7*F*), manual inspection of glycopeptide spectra demonstrate the miss assignment of glycopeptides possessing multiple individual glycosylation events as HexNAc_2_ events (Fig. S12). Combined, these data demonstrate that both modulating UppS activity and enhancing glycan translocation of HexNAc_2,_ but not HexNAc, alleviates the growth defects associated with initiating glycosylation in strains defective in completing the Burkholderia O-linked glycan.

### Modulation of early steps in *ogc* biosynthesis are also detrimental to *B. cenocepacia* viability

**Figure 8:**
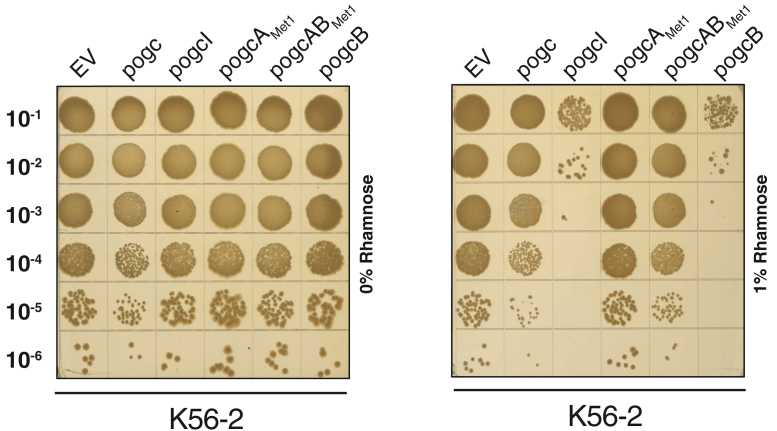
**Modulation of early steps in *ogc* biosynthesis impacts *B. cenocepacia* viability.** Spot plate assays of *B. cenocepacia* K56-2 (WT) containing the expression vectors pSCrhaB2 (EV), pSCrhaB2-*ogc (*p*ogc)*, pSCrhaB2-*ogcI (*p*ogcI)*, pSCrhaB2-*ogcA-Met1 (*p*ogcA*_Met1_), pSCrhaB2-*ogcB* (p*ogcB*), pSCrhaB2-*ogcAB-Met1* (p*ogcAB*_Met1_). Induction of *ogc*I and *ogc*B results in reduced viability (n = 4).

Finally, as changes driven by *ogc* perturbations were detrimental we questioned if shifting the equilibrium of the Und-P/Und-PP pool associated with the *ogc* alone would also impact viability. To probe this, we overexpressed components of the *ogc* within the parental *B. cenocepacia* K56-2 background and assessed viability utilizing spot assays. Introduction of rhamnose inducible vectors containing *ogcI* (p*ogcI*), *ogcB* (p*ogcB*), *ogcA* (p*ogcA*_*Met1*_) as well as the vector p*ogcAB*_*Met1*_*, which* allows the expression of both *ogcA* and *ogcB,* revealed induction of p*ogcI* and p*ogcB* resulted in reduced viability, while the overexpression of *ogcA*(p*ogcA*), *ogcAB* (p*ogcAB*), and the complete *ogc* (p*ogc*) showed no impact on viability (Fig. 8). Complementation of *B. cenocepacia* Δ*ogcAB,* Δ*ogcB*, and Δ*ogcI* with p*ogcAB*_*Met1*_, p*ogcB*, and p*ogcI* induced with 0.05% rhamnose-restored glycosylation, confirming the functionality of these constructs (Figs. S13–S15, Tables S17–S22). These observations support that the overexpression of early steps in *O*-linked glycan biosynthesis, OgcI and OgcB appear to impact viability, yet these effects can be alleviated in the case of *ogcB* by co-expression of *ogcA*. Combined, these observations support alterations in early steps in *O*-linked glycan biosynthesis modulate *ogc*-associated viability supporting the sensitivity of the Und-P/Und-PP pool to sequestration from non-physiological expression levels of *ogc* proteins.

## Discussion

*Burkholderia O*-linked glycosylation, mediated by the *ogc* and *pglL* (47, 59), are highly conserved at the pathway and glycan composition levels (45, 46, 47). While previous work demonstrated the requirement of the *ogc* for the generation of the trisaccharide used for glycosylation (47), a deeper understanding of how glycan fidelity is controlled and why limited glycan diversity is observed within *B. cenocepacia* has not been assessed. In line with the growing recognition of the impact of Und-P/Und-PP sequestration by dead-end intermediates of O-antigens (6, 11), capsule (35), cell wall teichoic acids (71, 72) and the ECA (7, 10) glycans, we demonstrate that blocking *ogc* leads to sequestration of the Und-P/Und-PP pool. In line with previous studies on Wzx-dependent glycan pathways (3, 4, 10, 27) this work provides insight into the mechanism responsible for the near-exclusive observation of Gal–GalNAc_2_ glycans within *Burkholderia* glycoproteomic studies to date (46, 47, 59, 73, 74, 75).

The conservation of the *Burkholderia O*-linked glycan is unique among characterized bacterial protein glycosylation systems to date, including the *O*-linked glycosylation systems of *Acinetobacter* (76, 77, 78) and *Neisseria* (40, 58, 79), as well as the *N*-linked glycosylation systems of *Campylobacter* (38, 39) where extensive glycan diversity has been observed. While the conservation of glycans in the context of protein glycosylation systems appears atypical, the conservation of glycan biosynthesis across species, or even genera, has previously been noted, as in the case of the ECA (80, 81). For the ECA, it has been suggested that the invariance of this oligosaccharide underpins its importance to Enterobacterales physiology (81). While the function of *Burkholderia O*-linked glycosylation is still unknown, this work raises a critical question on the importance of the cognate glycan in glycosylation. Despite the identification of *B. cenocepacia* protein glycosylation over a decade ago, we are yet to identify a specific function for glycosylation nor have individual glycoproteins been identified that require glycosylation for functionality (59, 63, 75). As demonstrated for other Wzx, non-native substrates can be translocated under heightened levels of flippase expression (14, 15, 16) and consistent with this, we demonstrate that overexpression of OgcX enhances the translocation of non-cognate *ogc* structures (Fig. 7, *F*/G). These overexpression studies support the role of OgcX as a flippase and confirm the function of OgcA as the glycosyltransferase responsible for adding the final Gal residue of the *O*-linked trisaccharide. Importantly, this ability to enhance translocation within Δ*ogcI*Δ*ogcA* Tn7-*ogcI* allows us to demonstrate that poor translocation efficiency appears to underpin the detrimental effects observed from the loss of OgcA (Fig. 7, *B*–*E*). Thus, this work provides experimental confirmation of the roles of OgcX as well as OgcA, highlights how *O*-linked glycan fidelity is controlled, and supports the importance of glycosylation with specific glycan structures within *B. cenocepacia* for membrane stress resistance.

Our observations of deleterious effects in response to the loss of *ogcX* (Figure 1, Figure 2, Figure 3) suggest the previously reported Δ*ogcX* isolate within *B. cenocepacia* (47) likely possessed an uncharacterized suppressor mutation. As noted previously, suppressor mutations can rapidly emerge upon Und-P/Und-PP sequestration (9, 33, 34) with the emergence of a potential suppressor mutation highlighted as the potential source of confounding results associated with defining the function of the WzxE flippase within *Escherichia coli* K-12 (10). While we were unable to obtain the previously reported Δ*ogcX* isolate, the observation that low-level glycosylation of a truncated glycan of a single HexNAc residue was observed within this strain (47) suggests a potential disruption within the *O-*linked glycosylation system. In contrast to previous results, our observation of the requirement of OgcX for *O*-glycosylation supports that this flippase is indispensable within *B. cenocepacia* for translocation of the *O*-linked glycan into the periplasmic space. Within other Gram-negatives such as *E. coli* K-12 not all flippases are indispensable and redundancy can exist with the ECA able to be mobilized by the O-antigen (Wzx_O16_) or colanic acid (WzxC) flippases (82). Our analysis of glycosylation in the absence of *ogcX* (Fig. 2*C*) is consistent with a lack of functional redundancy for the cognate *O*-linked glycan by the remaining Wzx flippases of *B. cenocepacia.* Within *B. cenocepacia* K56-2, previous analysis has highlighted the presence of at least five putative Wzx flippases with similar functional indispensability observed for the peptidoglycan flippase murJ_Bc_ (BCAL2764) (83). Thus, these findings support that OgcX is the sole flippase required for translocation of the *O*-linked glycan, and alternative flippases are not functionally redundant for this glycan in *B. cenocepacia.*

We have previously reported the inability to generate Δ*ogcA* within *B. cenocepacia* (47), yet disruptions of *ogcA* were recently suggested to be tolerated (61). Our data assessing the loss of *ogcA* using plasmid-based complementation (Fig. 1) as well as strains with inducible control of glycosylation (Figure 2, Figure 3, Figure 4, Figure 5, Figure 6, Figure 7) supports that, akin to *ogcX*, the loss of *ogcA* is deleterious within *B. cenocepacia*. Previously, putative Δ*ogcA* candidates were observed within high-density transposon libraries, yet *ogcA* CRISPRi silencing failed to recapitulate the resulting phenotypes associated with Δ*ogcA* (61). As such, we suggest that the Δ*ogcA* reported within prior work may contain unexpected secondary mutations or that the presence of transposons within *ogcA* may have adversely affected the expression of neighboring genes of the *ogc*. While transposon libraries coupled with sequencing provide a robust means to assess gene function at a genomic scale (84), it is recognized that this approach can lead to inactivation or polar effects in neighboring genes (85, 86). Furthermore, the enrichment of transposon junctions prior to sequencing (84) does not allow the assessment of unlinked potential suppressor or inactivation events, which may restore variability even in the presence of insertions into conditionally essential genes. Of note within our work, the utilization of proteomics allowed the assessment of *ogcA*, ogc*X,* and ogc*B* mutagenesis, revealing neighboring *ogc* proteins are unaffected by the removal of these genes (Fig. S5). Considering our findings, in addition to prior transposon studies of *B. cenocepacia* and *Burkholderia pseudomallei* that assign *ogcA* as essential (60, 62), this supports the assignment of *ogcA* as conditionally essential.

The observation that both *ogcA* and *ogcX* appear conditionally essential is consistent with a growing body of work highlighting the detrimental impacts of blocking Und-P/PP biosynthetic pathways (6, 7, 11). As with previous studies on the deleterious impacts of Und-P/PP sequestration, the use of inducible systems (10, 11, 14) provided a tractable way to explore the roles of *ogcA*/*ogcX* while limiting potential confounding effects from the emergence of suppressors. The inducible deleterious effects from the loss of *ogcA* and *ogcX* (Fig. 2) support fouling of the Und-P/PP pool with complementation confirming the restoration of glycosylation as well as suppression of growth defects by the re-introduction of *ogcX*/*ogcA* (Figs. 3 and 5). Within this work we noted that while the introduction of plasmid variants lacking *ogcA/X* were unable to be recovered from *B. cenocepacia* Δ*ogc* rhamnose inducible strains lacking *ogcA/X* were viable although exhibited reduced growth rates (Figs. 2 and S8). While the viability of inducible Δ*ogc*IΔ*ogc*A Tn7-ogcI *and* Δ*ogc*IΔ*ogc*X Tn7-ogcI strains was essential for allowing proteomic (Fig. 4) and phenotypic assessments (Figure 5, Figure 6, Figure 7), this does suggest the overall restoration of glycosylation mediated by Tn7-rha-*ogcI* may be reduced compared to wild type *B. cenocepacia,* reducing the magnitude of the effect of sequestration on the Und-P/PP pool. In line with this, we noted higher overall glycosylation levels, as determined by identified glycopeptides, within wild-type *B. cenocepacia* (Fig. 1*C*) compared to the observed levels within glycosylation inducible strains (Figure 2, Figure 3*F* and 7*F*) consistent with an overall reduced glycosylation capacity. Regardless, the ability to demonstrate changes in membrane permeability and stress sensitivity (Figs. 5 and 6) highlights the importance of *O*-linked glycan completion for *B. cenocepacia* physiology as well as allowed the assessment of antibiotic sensitization previously suggested for Δ*ogcA* (61). To our knowledge, this is the first study to probe the proteomic impacts of Und-P sequestration, providing insight into the protein-level alterations underpinning the observed changes in viability, membrane permeability, and stress sensitivity.

Our work demonstrates that upon initiation of glycosylation both Δ*ogcA and* Δ*ogcX* become sensitized to clinically important antibiotics including tetracycline, rifampicin, trimethoprim as well as ceftazidime (Fig. 6). This enhanced sensitivity also reconciles the differences we observe between experiments undertaken using plasmids (Figs. 1*D*, 3*A*, 3*D*, 5*B*, 5*C*, 7*B* and S4) compared to inducible strains alone (Figs. 2*B* and 5*A*) supporting that the antibiotic selection required to maintain plasmids exacerbates the observed viability defect within Δ*ogcA and* Δ*ogcX strains*. It should be noted that the observation that inhibiting *ogcA* or *ogcX* can modulate antibiotic sensitization suggests targeting the *ogc* may be a potent way to sensitize *Burkholderia* to antibiotics. The concept of modulating the Und-P/PP pool to sensitize *B. cenocepacia* has been previously explored by the depletion of *dxr* (BCAL2085) (87), which directly contributes to the precursors required for UppS-mediated *de novo* synthesis of Und-P (1, 88). Consistent with this concept, we note that both Δ*ogc*A and Δ*ogc*X possess enhanced sensitivity to Bacitracin upon the initiation of glycosylation (Fig. S16), further demonstrating the potential to exploit fouling of the Und-P/Und-PP pool through inhibiting glycosylation. Thus, fouling of the Und-P/PP pool through the inhibition of OgcX or OgcA may be an attractive approach for drug re-sensitization.

Previous studies have noted that the inhibition of peptidoglycan biosynthesis is likely the key defect responsible for the deleterious effects of Und-P/PP fouling (6, 7, 70). Consistent with this our DIA proteomic analysis reveals an increase in several proteins associated with peptidoglycan upon initiation of glycosylation within Δ*ogcA/X* strains including MurA (BCAL0310), MtgA (BCAL3412), FtsW (BCAL3463) and BCAL2777, a putative N-acetylmuramoyl-L-alanine amidase (Table S13, Fig. S17). Interestingly, among the altered membrane proteins, one of the most significant changes observed within Δ*ogcA* and Δ*ogcX* corresponds to a >30-fold increase in BCAM1996 (CreD, Fig. 4*F*). Within *Pseudomonas aeruginosa* CreD is known to be associated with sensing changes in peptidoglycan synthesis being potently transcribed in response to perturbations in peptidoglycan binding proteins (65) and suggested to be important for peptidoglycan Und-P/Und-PP recycling (89). Similarly, within *Stenotrophomonas maltophilia* CreD has also been shown to be critical for envelope integrity and maintaining cell morphology (90). The increased abundance of CreD in response to glycosylation initiation within Δ*ogc*IΔ*ogc*A Tn7-ogcI *and* Δ*ogc*IΔ*ogc*X Tn7-ogcI may suggest a more general role for CreD in Und-P/Und-PP recycling. Of the proteomic alterations observed the changes within Δ*ogc*IΔ*ogc*A Tn7-ogcI and Δ*ogc*IΔ*ogc*X Tn7-ogcI are similar yet more extensive than the changes observed from the loss of glycosylation alone (Fig. S7) (63). While this work demonstrates widespread alterations within the membrane proteome of *B. cenocepacia*, the lack of detailed annotation information does limit our ability to rationalize the potential functional roles of many of these membrane proteins. Regardless, these observations support that blocking *ogc* biosynthesis induces extensive and pleiotropic effects, beyond the changes seen from the loss of protein glycosylation alone, including evidence of changes in peptidoglycan biosynthesis which are consistent with Und-P/PP fouling.

In this study, of the two putative UppS enzymes assessed only BCAL2087 was observed to alleviate the growth defects associated with glycosylation initiation in both the Δ*ogcA* and Δ*ogcX* supporting the role of this protein as a putative UppS in *B. cenocepacia* (Fig. 7*B*). Previous studies by Hogan *et al.* support that BCAL2087 is the primary Undecaprenyl Pyrophosphate Synthetase in *B. cenocepacia* based on the loss of growth upon CRISPRi silencing and the additive effect of this silencing within mutants possessing defects which impact the Und-P/PP pool (61). Consistent with changes in the Und-P/PP pool impacting viability our analysis of individual *ogc* components reveals OgcI and OgcB alone is sufficient to inhibit growth when overexpressed within *B. cenocepacia* (Fig. 8). These proteins do not themselves impact growth within *E. coli* (Fig.S 18) and when induced with 0.05% rhamnose restore glycosylation within *B. cenocepacia* Δ*ogcB* and Δ*ogcI* (Figs. S14 and S15). Thus, this reduced viability observed upon induction of OgcI and OgcB supports that increasing the proportion of early-stage intermediates in *ogc* biosynthesis leads to the sequestration of the Und-PP/P pool. In line with this model, the overexpression of OgcA with OgcB was found to relieve toxicity, supporting the importance of adequate enzyme capacities in meeting the demands during *ogc* biosynthesis. A caveat of this model is the assumption that the products of the *ogc* solely supply glycan units used for protein glycosylation, yet whether this is the case is unknown. As noted within other glycan biosynthesis pathways, both glycan intermediates as well as complete glycan units may be incorporated into different glycoconjugates (80, 81). Previous studies of *O*-linked glycosylation systems have demonstrated that glycans used for glycosylation may be derived from several Und-P/PP-dependent biosynthesis pathways, such as the O-antigen in *P. aeruginosa* (91, 92) and capsule pathways within the *Moraxellaceae* (93, 94). Considering this, while the *ogc* is sufficient and required for protein glycosylation (47) and we show the modulation of *ogc* intermediates impacts viability, it is currently unclear if the *ogc* pathway contributes solely to protein glycosylation.

In conclusion, this work demonstrates that the loss of *ogcA*/*X* within *B. cenocepacia* results in detrimental impacts on viability, membrane permeability, and the proteome, which appears to sensitize strains to membrane stresses, including clinically useful antibiotics. This work highlights that, akin to the impacts of glycan biosynthesis blockages observed in O-antigens (6, 9) and the ECA (7, 10), modulating or blocking the generation of *ogc* intermediates appears to lead to sequestration of the Und-P/PP pool within *B. cenocepacia.* These findings not only advance our mechanistic understanding of how glycan fidelity is maintained for Burkholderia *O*-linked protein glycosylation but the therapeutic potential of targeting steps in this pathway.

## Experimental procedures

### Bacterial strains and growth conditions

Bacterial strains used in this study are listed in Table S1. Strains were cultured in Lysogeny Broth (LB) or on LB agar (1.5–2% w/v), prepared in accordance with the manufacturer's instructions, containing 0.5% NaCl (BD). Liquid cultures were incubated overnight at 37 °C with shaking, while agar plates were incubated at 37 °C overnight for *E. coli* and 24 to 72 h for *B. cenocepacia*. When required, LB was supplemented with 200 μg/ml diaminopimelic acid (DAP; Sigma) to support the growth of *E. coli* RHO3 (95). Antibiotics were added to cultures to select or maintain plasmids/transconjugants at a final concentration of 50 μg/ml trimethoprim for *E. coli* and 100 μg/ml for *B. cenocepacia*, 20 μg/ml tetracycline for *E. coli* and 150 μg/ml for *B. cenocepacia,* and 50 μg/ml kanamycin for *E. coli*. To counter-select helper and donor *E. coli* strains during triparental and quadparental mating, ampicillin at 100 μg/ml and polymyxin B at 25 μg/ml were added (96). Culturing of strains containing the temperature-sensitive plasmid pFlptet (see Table S2) was undertaken at 30 °C with cultures shifted to 37 °C to enable plasmid curing (97). Curing of pDAI-SceI-SacB was achieved by sucrose counterselection as previously described with 5% sucrose in LB lacking NaCl (96). Induction of *B. cenocepacia* strains was performed by either adding 20% filter-sterilised L-rhamnose monohydrate (Sigma) or 1 mM 4-Isopropylbenzoic acid (cumate, Sigma) to LB broth or LB agar at a concentration of 0.05% to 1% for rhamnose and 100 μM for cumate. For non-induced controls, an equivalent volume of solvent (sterile water for rhamnose and ethanol for cumate) was added to the culture media.

### Recombinant DNA methods

Oligonucleotides used in this study are listed in Table S3, with all PCR amplifications for cloning carried out using Q5 DNA polymerase (New England Biolabs) with the addition of 2% DMSO for the amplification of *B. cenocepacia* DNA, due to its high GC content. Genomic DNA isolations were performed using an EZ-10 Spin Column Bacterial Genomic DNA Mini-Preps Kit (Bio Basic), while PCR clean-up and restriction digest purifications were performed with a Zymoclean Gel DNA Recovery Kit (ZymoResearch). pGPI-SceI (98) mutagenesis plasmids were constructed using Gibson assembly (99) with DNA fragments flanking genes of interest amplified by Q5-based PCR. *Sma*I-linearized pGPI-SceI and Q5-amplified fragments were assembled using the NEBuilder HiFi DNA master mix according to the manufacturer's instructions (New England Biolabs). pSCrhaB2- (64) and pMLBAD (100)-based expression vectors containing genes of interest were generated using Gibson Assembly or restriction endonuclease-based cloning. For Gibson Assembly, PCR-amplified fragments were introduced into *Nde*I/*Xba*I-linearized pSCrhaB2 or PCR-derived pMLBAD vector backbones using the NEBuilder HiFi DNA master mix. For restriction endonuclease-based cloning, PCR-amplified fragments were digested with *Nde*I/*Xba*I and then ligated into *Nde*I/*Xba*I-linearized pSCrhaB2 overnight at 16°C using T4 Ligase (New England Biolabs). Mutagenesis of pSCrhaB2-ogc to remove *ogc*A, *ogc*B, *ogc*I and *ogc*X was achieved by assembling PCR-amplified fragments into *Nde*I/*Hind*III-linearized pSCrhaB2 using the NEBuilder HiFi DNA master mix.

Prior to Gibson Assembly and restriction cloning, all DNA fragments were assessed for correctness based on size using agarose gel electrophoresis. Ligated/assembled plasmid mixtures were introduced into chemically competent *E. coli* PIR2 cells *via* heat shock transformation (101), plated on selective media, and screened using colony PCR with GoTaq Green Master Mix (Promega). All plasmids used in this study have been confirmed by either Sanger sequencing using the Australian Genome Research Facility or nanopore plasmid sequencing using Plasmidsaurus (SNPsaurus LLC). Donor plasmids for biparental and quadparental mating were further isolated and introduced into *E. coli* RHO3 (95) *via* electroporation (101) to improve conjugation efficiency into *B. cenocepacia*. A complete list of the plasmids used in this study, along with a summary of their construction, is provided in Table S2.

### Conjugation of plasmids into *B. cenocepacia*

Plasmids were introduced into *B. cenocepacia* K56–2 *via* one of three approaches: I) biparental mating using the conjugative diaminopimelic acid auxotroph *E. coli* RHO3 (95) for the introduction of pFLPtet or pDAI-SceI-SacB; II) triparental mating (96) using *E. coli* PIR2 containing pRK2013 (helper plasmid) (102) and *E. coli* PIR2 carrying pGPI-SceI-derived vectors or pSCrhaB2/pMLBAD expression vectors (donor plasmid); or III) quadparental mating using *E. coli* PIR2 containing pRK2013 (102), *E. coli* PIR2 containing pTNS3 (Tn7 transposase plasmid) (103) and *E. coli* RHO3 carrying pUC18T-mini-Tn7T-Tp-rha-ogcI (donor plasmid) for the chromosomal integration of miniTn7-rha-ogcI. Conjugations were allowed to proceed for 24 h, then successful transconjugants were selected with trimethoprim, and the introduction of plasmids of interest was confirmed using PCR-based screening. For the generation of unmarked, non-polar deletions using pGPI-SceI and the removal of the trimethoprim resistance marker within the integrated miniTn7, additional rounds of conjugation were undertaken to introduce pDAI-SceI-SacB (98, 104) or pFLPtet (97), respectively, using tetracycline-based selection. pGPI-SceI/pDAI-SceI-SacB-based mutagenesis as well as FLP-based removal of the trimethoprim resistance marker within Tn7 were confirmed using screening oligonucleotides (Table S3) by colony PCR using GoTaq Green Master Mix. Numeration of transconjugants derived from the introduction of pSCrhaB2-*ogc* and its derivatives into *B. cenocepacia* wildtype (WT) or *B. cenocepacia* Δ*ogc* were assessed across four independent conjugations.

### Spot plate assays

Colony size morphology and membrane stress tolerance assessments were performed as previously described (105, 106, 107) using spot plate assays. For spot plate assays, strains of interest were grown overnight in LB broth without induction, normalized to an OD_600_ of 1.0 and then serially diluted from 10^–1^ to 10^–6^. 10 μl of diluted cultures were manually spotted onto LB plates with and without inducers (1% Rhamnose/100 μM cumate). 0.01% SDS or 2% NaCl were added to plates to assess membrane integrity and the impact of osmotic stress on viability with and without inducers. For assays of *B. cenocepacia* strains containing pSCrhaB2- or pMLBAD-based expression vectors, trimethoprim was added to plates to ensure the maintenance of plasmids. For antimicrobial resistance, spot assays concentrations corresponding to at or below the broth dilution defined MIC assays (Fig. S11) were used for trimethoprim (8 μg/ml), tetracycline (16 μg/ml), chlorhexidine (4 μg/ml), ceftazidime (4 μg/ml), and rifampicin (32 μg/ml) on cation-adjusted Mueller–Hinton agar. Assays to assess Bacitracin sensitivity were undertaken using 300 μg/ml of Bacitracin on LB agar plates. Spots were allowed to dry, and plates were incubated for 48 h at 37 °C before being imaged. All spot assays were assessed using at least three independent replicates. Colony area measurements were undertaken using Fiji (108), assessing the size of 20 colonies per spot assay with independent assays conducted with *t*-tests used to determine significant changes in colony size as well as viable counts between induced and non-induced conditions.

### Microtiter plate-based viability and growth kinetics assays

To assess viability and growth kinetics, Microtiter 96-well-based growth assays were undertaken as previously described (63). Bacterial strains were grown overnight in LB, normalized to an OD_600_ of 1.0 and diluted one in 100 with LB supplemented with or without combinations of inducers and/or membrane stressors (0.01% SDS or 2% NaCl). 200 μl of diluted cultures were added to 96-well flat-bottom plates and incubated at 37 °C with shaking at 200 rpm in a CLARIOstar plate reader (BMG LABTECH, Inc.), measuring the optical density at 600 nm every 10 min over 24 h. Growth assays were assessed in at least technical triplicate across three independent biological replicates. To assess Bacitracin sensitivity, plates grown at 37 °C as above for 48 h were assessed for their optical density at 600 nm with Bacitracin at 2 mg/ml, 1 mg/ml, 0.5 mg/ml, 0.25 mg/ml, 0.125 mg/ml, 0.0625 mg/ml, and 0.03125 mg/ml with and without 1% Rhamnose.

### Fluorescence detection of the permeability of inner and outer membranes

Permeability of the inner membrane was assessed using Hoechst 33,342 (Abcam) as previously described (109, 110, 111). Briefly, bacterial strains were grown overnight in LB (with and without 1% Rhamnose), normalized to an OD_600_ of 2.0, washed three times with PBS and dispensed at 100 μl aliquots into 96-well black clear-bottomed plates. 100 μl of PBS and heat-killed bacteria were added to the plates as controls. 100 μl of 2.5 μM Hoechst 33,342 in PBS was added to wells to generate bacterial suspensions with an OD_600_ of 1.0 and a final concentration of 1.25 μM Hoechst 33,342. Fluorescence was measured in a CLARIOstar plate reader using an excitation of 361 nm and emission detection at 486 nm. The fluorescence intensity was first normalized by assigning the value of 100 to the heat-killed and 0 to the non-induced parental strain. To assess outer membrane permeability, 1-*N*-phenylnaphthylamine (NPN, Thermo Fisher Scientific) was used as previously described (69, 112, 113). Cultures were normalized as above, washed three times with 5 mM HEPES, pH 7.4, 10 mM sodium azide and dispensed at 100 μl aliquots into 96-well black clear-bottomed plates. 100 μl of 5 mM HEPES, pH 7.4, 10 mM sodium azide was added to the plates as a blank. 100 μl of 20 μM NPN in 5 mM HEPES, pH 7.4, 10 mM sodium azide was added to wells to generate bacterial suspensions with an OD_600_ of 1.0 and a final concentration of 100 μM NPN. Fluorescence was measured in a CLARIOstar plate reader using an excitation of 350 nm and emission detection at 420 nm. Hoechst 33,342 and NPN assays were assessed in technical triplicate across five independent biological replicates comparing changes in fluorescence intensity to the non-induced parental strain. To assess viability and ensure comparable cell numbers, the OD-adjusted bacterial suspensions were serially diluted and plated on LB and viable cells numerated (Fig. S9). The fluorescence intensity from both assays was normalized against the determined cell number.

### Proteomic sample preparation and LC-MS analysis

*B. cenocepacia* cultures for proteomic analysis were grown overnight with or without induction with rhamnose and/or cumate as outlined above with shaking at 180 rpm. Overnight cultures were normalized to an OD_600nm_ of 1.0 and then collected by centrifugation at 10,000*g* at 4 °C for 10 min, washed 3 times withice-cold PBS and then snap frozen at −80 °C until processing. Frozen whole cell samples were prepared for analysis using sodium deoxycholate (SDC) based lysis and the in-StageTip preparation approach as previously described (114). For proteomic analysis of OgcX complementation SDS-based lysis and S-trap sample preparation was undertaken to improve the detection of OgcX. Following digestion, samples were cleaned up using SDB-RPS (Sigma) StageTips (114, 115, 116) and stored at −20 °C prior to analysis. Cleaned-up peptide samples were re-suspended in Buffer A∗ (2% acetonitrile, 0.1% trifluoroacetic acid in Milli-Q water) and separated using a two-column chromatography set-up on a Dionex Ultimate 3000 UPLC composed of a PepMap100 C18 20 mm × 75 μm trap and a PepMap C18 500 mm × 75 μm analytical column (Thermo Fisher Scientific) coupled to a Orbitrap Fusion Lumos Tribrid Mass Spectrometer (Thermo Fisher Scientific) with datasets collected with and without the use of the FAIMS Pro interface (Thermo Fisher Scientific). A complete description of the sample preparation methods, the Data-Dependent Acquisition (DDA) and Data-Independent Acquisition (DIA) LC-MS methods as well as the associated bioinformatic analysis used to identify/quantify the proteomics datasets are provided within the Supplementary Methods.

## Data availability

All mass spectrometry data (RAW files, FragPipe outputs, Spectronaut outputs/experiment files, Rmarkdown scripts, and output tables) have been deposited into the PRIDE ProteomeXchange repository (117, 118). All PRIDE accession numbers, descriptions of the associated experiments and experiment types (DIA or DDA) are provided in Table S4.

## Supporting information

This article contains supporting information (73, 120, 121, 122, 123, 124, 125, 126, 127, 128, 129, 130, 131, 132).

## Conflict of interest

The authors declare that they have no conflicts of interest with the contents of this article.
