# Supplementary material for: The late-stage steps of Burkholderia cenocepacia protein O-linked glycan biosynthesis are conditionally essential
Source: J Biol Chem. 2025 Apr 24;301(6):108515. doi: 10.1016/j.jbc.2025.108515 (PMC12152626; doi:10.1016/j.jbc.2025.108515)
Supplement: Supplementary Tables and Figures [file mmc1.docx]

**The Late-Stage Steps of *Burkholderia cenocepacia* Protein *O*-Linked Glycan Biosynthesis Are Conditionally Essential.**

Leila Jebeli^1^, Taylor A. McDaniels^1^, Duncan T. T. Ho^1^, Hamza Tahir^1^, Nicholas L. Kai-Ming^1^, Molli Mcgaw^1^, Kristian I. Karlic^1^, Jessica M. Lewis^2^ and Nichollas E. Scott^1^

^1^Department of Microbiology and Immunology, University of Melbourne at the Peter Doherty Institute for Infection and Immunity, Melbourne 3000, Australia

Current addresses:

^2^School of Life Sciences, University of Warwick, Coventry, CV4 7AL, UK.

^#^To whom correspondence and requests for materials should be addressed N.E.S ([Nichollas.scott@unimelb.edu.au](mailto:Nichollas.scott@unimelb.edu.au))

**Key words:** Glycosylation, *Burkholderia cenocepacia*, *Burkholderia*, Post-translational modifications, Proteomics, Glycoproteomics

**Table of Contents**

| **Title** | **Page** |
| --- | --- |
| **Supplementary Table 1: Strain list** | **4** |
| **Supplementary Table 2: Plasmid list** | **5** |
| **Supplementary Table 3: Primer list** | **8** |
| **Supplementary Table 4: Proteomic datasets** | **14** |
| **Supplementary Table 5: Antimicrobial Minimal inhibitory concentrations (MIC).** | **17** |
| **Supplementary Tables 6 – 22: Supplementary Proteomic tables** | **18** |
| **Supplementary Methods: Complete Proteomics and LC-MS methods** | **22** |
| **Supplementary Figure 1: Proteomic/glycoproteomic validation of *B. cenocepacia* Tn7-*ogcI*.** | **25** |
| **Supplementary Figure 2. Quantitative assessment of Δ*ogcX,* Δ*ogcA,* Δ*ogcB and* Δ*ogcAB* colony sizes with and without 1% rhamnose induction.** | **26** |
| **Supplementary Figure 3. Start codon assignments of OgcA used for OgcA complementation within *B. cenocepacia* Δ*ogcI* Δ*ogcA* Tn7-*ogcI.*** | **27** |
| **Supplementary Figure 4. Viability of *B. cenocepacia* Δ*ogcI*Δ*ogcA* Tn7-*ogcI* and Δ*ogcI*Δ*ogcX* Tn7-ogcI containing control plasmids in response to induction.** | **28** |
| **Supplementary Figure 5. Proteomic analysis of the proteins of the *ogc* within Δ*ogcI*Δ*ogcX* Tn7-*ogcI*, Δ*ogcI*Δ*ogcA* Tn7-*ogcI*, Δ*ogcI*Δ*ogcB* Tn7-*ogcI* and Δ*ogcI* Tn7-*ogcI* strains.** | **29** |
| **Supplementary Figure 6. Proteomic analysis of *B. cenocepacia* Δ*ogcI* Tn7-*ogcI* strains.** | **30** |
| **Supplementary Figure 7. Comparison of proteomic alterations observed within *B. cenocepacia* Δ*ogc*IΔ*ogc*X Tn7-*ogc*I and Δ*ogc*IΔ*ogc*A Tn7-ogcI compared to *B. cenocepacia* glycosylation-null strains.** | **31** |
| **Supplementary Figure 8. Plate-based growth assays of Δ*ogcI* Tn7-*ogcI,* Δ*ogcI*Δ*ogcX* Tn7-*ogcI*, Δ*ogcI*Δ*ogcA* Tn7-*ogcI*, Δ*ogcI*Δ*ogcAB* Tn7-*ogcI* and Δ*ogcI*Δ*ogcB* Tn7-*ogcI* strains in the presence of membrane / osmotic stress agents.** | **32** |
| **Supplementary Figure 9. Viable count for Hoechst and NPN uptake assays.** | **33** |
| **Supplementary Figure 10. Hoechst and NPN uptake assays of complemented Δ*ogc*IΔ*ogc*A Tn7-*ogc*I and Δ*ogc*IΔ*ogc*X Tn7-*ogc*I.** | **34** |
| **Supplementary Figure 11. Antibiotic susceptibility assays of glycosylation inducible *B. cenocepacia* strains, with and without 1% Rhamnose induction.** | **35** |
| **Supplementary Figure 12. *B. cenocepacia* Δ*ogcI*Δ*ogcB* Tn7-*ogcI* Glycopeptides.** | **36** |
| **Supplementary Figure 13. Proteomics analysis of pSCrhaB2-*ogcAB in* Δ*ogcAB*.** | **37** |
| **Supplementary Figure 14. Proteomics analysis of pSCrhaB2-*ogcB* in Δ*ogcB*.** | **38** |
| **Supplementary Figure 15. Proteomics analysis of pSCrhaB2-*ogcI* in Δ*ogcI*.** | **39** |
| **Supplementary Figure 16. Loss of *ogcA* and *ogcX* sensitises *B. cenocepacia* to sub-inhibitory concentrations of Bacitracin.** | **40** |
| **Supplementary Figure 17. Proteomic analysis of peptidoglycan associated proteins within Δ*ogcI*Δ*ogcA* Tn7-*ogcI* and Δ*ogcI*Δ*ogcX* Tn7-*ogcI* in response to glycosylation initiation.** | **41** |
| **Supplementary Figure 18. Spot plate assays of *E. coli* containing the expression vectors pSCrhaB2 (EV), pSCrhaB2-*ogcI (*p*ogcI)* and pSCrhaB2-*ogcB* (p*ogcB*).** | **42** |
| **References.** | **43** |

**Supplementary Document Table 1: Strain list**

| **Strain** | **Description** | **Source** |
| --- | --- | --- |
| ***E. coli*** | | |
| DH5α | F^−^ Φ80*lac*ZΔM15 Δ(*lac*ZYA-*argF*) U169 *rec*A1 *end*A1 *hsd*R17(r_K_^–^, m_K_^+^) *pho*A *sup*E44 *thi*-1 *gyr*A96 *rel*A1 λ^–^ | Invitrogen |
| PIR2 | F^−^ ∆*lac*169 *rpo*S(am) *rob*A1 *cre*C510 *hsd*R514 *end*A *rec*A1 *uidA*(∆*Mlu*I)::*pir* | Thermo Scientific |
| RHO3 | Δ*asd* *thi*-1 *thr*-1 *leu*B26 *ton*A21 lacY1 *sup*E44 *rec*A; integrated RP4-2 Tcr::Mu Δ*aphA* (λpir+) | ^1^ |
| ***B. cenocepacia*** | | |
| K56-2 WT | CF clinical isolate of the ET12 lineage, closely related to *B. cenocepacia* J2315 | Canadian *B. cepacia* research and referral repository ^2^ |
| K56-2 Δ*ogc* | Unmarked *ogc* (BCAL3114–BCAL3118) deletion | ^3^ |
| K56-2 Δ*ogcAB* | Unmarked *ogcAB* (BCAL3114–BCAL3118) deletion | ^3^ |
| K56-2 Δ*ogcB* | unmarked *ogcB* (BCAL3116) deletion generated using pGPI-SceI-*ogcB*/pDAI-SceI-SacB | This study |
| K56-2 Δ*ogcI* | Unmarked *ogcI* (BCAL3118) deletion generated using pGPI-SceI-*ogcI*/pDAI-SceI-SacB | This study |
| K56-2 Δ*ogcI* Tn7-*ogcI* | Trimethoprim sensitive (Tmp^S^) tetracycline sensitive (Tet^S^), Δ*ogcI* strain with chromosomally integrated *ogcI* under the rhamnose inducible promoter p_Rha_ with *rhaS/R* downstream of *glmS1* (BCAL0611) created using the miniTn7 delivery system. Tmp resistance cassette from miniTn7 element was subsequently removed using pFlpTet, and the strain was cured of pFlpTet. | This study |
| K56-2 Δ*ogcI* Tn7-*ogcI* Δ*ogcX* | K56-2 Δ*ogcI* Tn7-*ogcI* with unmarked *ogcX* (BCAL3114) deletion generated using pGPI-SceI-*ogcX*/pDAI-SceI-SacB | This study |
| K56-2 Δ*ogcI* Tn7-*ogcI* Δ*ogcA* | K56-2 Δ*ogcI* Tn7-*ogcI* with unmarked *ogcA* (BCAL3115) deletion generated using pGPI-SceI-*ogcA*/pDAI-SceI-SacB | This study |
| K56-2 Δ*ogcI* Tn7-*ogcI* Δ*ogcB* | K56-2 Δ*ogcI* Tn7-*ogcI* with unmarked *ogcB* (BCAL3116) deletion generated using pGPI-SceI-*ogcB*/pDAI-SceI-SacB | This study |
| K56-2 Δ*ogcI* Tn7-*ogcI* Δ*ogcAB* | K56-2 Δ*ogcI* Tn7-*ogcI* with unmarked *ogcAB* (BCAL3115-6) deletion generated using pGPI-SceI-*ogcAB*/pDAI-SceI-SacB | This study |

**Supplementary Document Table 2: Plasmid list**

| **Plasmid** | **Description** | **Source** |
| --- | --- | --- |
| pRK2013 | Helper plasmid for conjugation*, ori*_colE1_, RK2 derivative, Kan^R^, *mob*^+^, *tra*^+^ | ^4^ |
| pFlpTet | pFlpe4-derived plasmid, rhamnose-inducible *flp* (encoding flippase), Tet^R^, temperature-sensitive | ^5^ |
| pDAI-SceI-SacB | *ori*_pBBR_, Tet^R^, P_dhfr_, *mob^+^*, expressing I-SceI and SacB | ^6^ |
| pTNS3 | *ori*_R6K_, Amp^R^, expressing *tnsABCD* from *P1* and *P_lac_* (for Tn7 site-specific transposition). Addgene Plasmid #63127 | ^7^ |
| pUC18T-mini-Tn7T-Tp | mini-Tn7 suicide vector, *ori*_R6K_, *oriT* (for mobilisation), Amp^R^/Tmp^R^. Addgene Plasmid #65024 | ^7^ |
| pUC18T-mini-Tn7T-Tp-rha-ogcI | mini-Tn7 element containing rhamnose-inducible ogcI (BCAL3118) generated by subcloning the rhamnose-inducible *ogcI* element from pSCrhaB2-ogcI using *Nsi*I/*Hind*III into pUC18T-mini-Tn7T linearised with *Nsi*I/*Hind*III, Amp^R^ / Tmp^R^, | This study |
| pSCrhaB2 | *ori*_pBBR1_, *rhaR*, *rhaS*, P*_rhaB_*, *mob^+^,* Tmp^R^, Addgene Plasmid #113634 | ^8^ |
| pSCrhaB2-ogcI | *ogcI* (BCAL3118) cloned into pSCrhaB2 under the rhamnose-inducible promoter using Gibson assembly using the PCR product of primers Nsco_0526 / Nsco_0527 into *Nde*I/*Xba*I-linearised pSCrhaB2, Tmp^R^ | This study |
| pSCrhaB2-ogcA-met1 | *ogcA* (BCAL3115) from the assigned GTG start codon (modified to ATG) cloned into pSCrhaB2 under the rhamnose-inducible promoter using Gibson assembly using the PCR product of primers Nsco_0566 / Nsco_0525 into *Nde*I/*Xba*I-linearised pSCrhaB2, Tmp^R^ | This study |
| pSCrhaB2-ogcA-met2 | *ogcA* (BCAL3115) from the putative alternative start codon of *ogcA* located within *ogcX* cloned into pSCrhaB2 under the rhamnose-inducible promoter using Gibson assembly using the PCR product of primers Nsco_0567 / Nsco_0525 into *Nde*I/*Xba*I-linearised pSCrhaB2, Tmp^R^ | This study |
| pSCrhaB2-ogcAB-met1 | *ogcAB* (BCAL3115-6) from the assigned GTG start codon (modified into ATG) of *ogc*A cloned into pSCrhaB2 under the rhamnose-inducible promoter by ligating the *Nde*I/*Xba*I-digested PCR product of primers Nsco_0215 / Nsco_0567 and *Nde*I/*Xba*I-linearised pSCrhaB2, Tmp^R^ | This study |
| pSCrhaB2-ogcAB-met2 | *ogcAB* (BCAL3115-6) from the putative alternative start codon of *ogcA* located within *ogcX* cloned into pSCrhaB2 under the rhamnose-inducible promoter by ligating the *Nde*I/*Xba*I-digested PCR product of primers Nsco_0215 / Nsco_0911 and *Nde*I/*Xba*I-linearised pSCrhaB2, Tmp^R^ | This study |
| pSCrhaB2-ogcB | *ogcB* (BCAL3116) cloned into pSCrhaB2 under the rhamnose-inducible promoter using Gibson assembly using the PCR product of primers Nsco_0214 / Nsco_0215 into *Nde*I/*Xba*I-linearised pSCrhaB2, Tmp^R^ | This study |
| pSCrhaB2-ogc | *ogc* (BCAL3114-BCAL3118) cloned into pSCrhaB2 under the rhamnose-inducible promoter using Gibson assembly using the PCR product of primers Nsco_0919 / Nsco_0920 and *Nde*I/*Xba*I-linearised pSCrhaB2, Tmp^R^ | This study |
| pSCrhaB2-ogcΔogcX | pSCrhaB2 containing the *ogc* cluster under the rhamnose-inducible promoter with *ogcX* (BCAL3114) removed. Generated using Gibson assembly of the PCR products of primers Nsco_0216 / Nsco_1187 and Nsco_0217 / Nsco_1186 and *Nde*I/*Hind*III-linearised pSCrhaB2, Tmp^R^ | This study |
| pSCrhaB2-ogcΔogcA | pSCrhaB2 containing the *ogc* cluster under the rhamnose-inducible promoter with *ogcA* (BCAL3115) removed. Generated using Gibson assembly of the PCR products of primers Nsco_0216 / Nsco_1189 and Nsco_0217 / Nsco_1188 and *Nde*I/*Hind*III-linearised pSCrhaB2, Tmp^R^ | This study |
| pSCrhaB2-ogcΔogcB | pSCrhaB2 containing the *ogc* cluster under the rhamnose-inducible promoter with *ogcB* (BCAL3116) removed. Generated using Gibson assembly of the PCR products of primers Nsco_0216 / Nsco_1191 and Nsco_0217 / Nsco_1190 and *Nde*I/*Hind*III-linearised pSCrhaB2, Tmp^R^ | This study |
| pSCrhaB2-ogcΔogcI | pSCrhaB2 containing the *ogc* cluster under the rhamnose-inducible promoter with *ogcI* (BCAL3118) removed. Generated using Gibson assembly of the PCR products of primers Nsco_0216 / Nsco_1193 and Nsco_0217 / Nsco_1192 and *Nde*I/*Hind*III-linearised pSCrhaB2, Tmp^R^ | This study |
| pGPI-SceI | *ori_R6K_*, *mob*^+^, I-SceI restriction site, Tmp^R^ | ^9^ |
| pGPI-SceI-ogcX | pGPI-SceI containing fragments flanking *ogcX* (BCAL3114) generated using Gibson assembly of the PCR products of primers Nsco_0279 / Nsco_0280 and Nsco_0281 / Nsco_0282 with *Sma*I-linearised pGPI-SceI, Tmp^R^ | This study |
| pGPI-SceI-ogcA | pGPI-SceI containing fragments flanking *ogcA* (BCAL3115) generated using Gibson assembly of the PCR products of primers Nsco_0176 / Nsco_0177 and Nsco_0178 / Nsco_0179 with *Sma*I-linearised pGPI-SceI, Tmp^R^ | This study |
| pGPI-SceI-ogcB | pGPI-SceI containing fragments flanking *ogcB* (BCAL3116) generated using Gibson assembly of the PCR products of primers Nsco_0182 / Nsco_0183 and Nsco_0184 / Nsco_0185 with *Sma*I-linearised pGPI-SceI, Tmp^R^ | This study |
| pGPI-SceI-ogcAB | pGPI-SceI containing fragments flanking *ogcAB* (BCAL3115-6) generated using Gibson assembly of the PCR products of primers Nsco_0213 / Nsco_0185 and Nsco_0194 / Nsco_0212 with *Sma*I-linearised pGPI-SceI, Tmp^R^ | This study |
| pGPI-SceI-ogcI | pGPI-SceI containing fragments flanking *ogcI* (BCAL3118) generated using Gibson assembly of the PCR products of primers Nsco_0271 / Nsco_0272 and Nsco_0273 / Nsco_0274 with *Sma*I-linearised pGPI-SceI, Tmp^R^ | This study |
| pUS250-sfGFP | Derivative of pUS250 (Addgene plasmid # 198322) containing the cumate-inducible system of *Pseudomonas putida* (CymR) regulating the expression of superfolder green fluorescent protein (*sfgfp*) gene, Kan^R^ | Nick Coleman (unpublished) |
| pMLBAD | *ori*_pBBR1_, *araC* P_BAD_, *mob^+^*, Tmp^R^ | ^10^ |
| pCumate-sfGFP | pMLBAD backbone containing *ori*_pBBR1_, *mob^+^*, modified to contain the cumate-inducible system from *P. putida* (CymR) regulating the expression of superfolder green fluorescent protein (*sfgfp*) gene generated using Gibson assembly of the PCR product of primers Nsco_1094 / Nsco_1095 from pUS250-sfGFP and the amplified pMLBAD generated using primers Nsco_1092/Nsco_1093, Tmp^R^ | This study |
| pCumate-ogcX-his | pMLBAD with cumate-inducible system from *P. putida* (CymR) regulating the expression of *ogcX*-His_6_ generated using Gibson assembly of the PCR product of primers Nsco_1453 / Nsco_1458 and the amplified pMLBAD-cumate backbone from pCumate-sfGFP generated using primers Nsco_1455 / Nsco_1457, Tmp^R^ | This study |
| pCumate-BCAL2087 | pMLBAD with cumate-inducible system from *P. putida* (CymR) regulating the expression of *uppS* (BCAL2087) generated using Gibson assembly of the PCR product of primers Nsco_1588 / Nsco_1589 and the amplified pMLBAD-cumate backbone from pCumate-sfGFP generated using primers Nsco_1590 / Nsco_1591, Tmp^R^ | This study |
| pCumate-BCAM2067 | pMLBAD with cumate-inducible system from *Pseudomonas putida* regulating the expression of *uppS* (BCAM2067) generated using Gibson assembly of the PCR product of primers Nsco_1594 / Nsco_1662 and the amplified pMLBAD-cumate backbone from pCumate-sfGFP generated using primers Nsco_1419 / Nsco_1420, Tmp^R^ | This study |

Kan^R^, kanamycin resistance, Tet^R^, tetracycline resistance, Amp^R^, ampicillin resistance, Tmp^R^, trimethoprim resistance

**Supplementary Document Table 3: Primer list**

| **Primer** | **Sequence** | **Description / Purpose** |
| --- | --- | --- |
| mini-Tn7 | | |
| Nsco_0190 | cgaaccgaacaggcttatgt | Forward screening primers to assess insertion into pUC18T-mini-Tn7T-Tp or mini-Tn7 integration into chromosome |
| Nsco_0191 | ctgtgggcggacaaaatagt | Reverse screening primers to assess of insertion into pUC18T-mini-Tn7T-Tp or mini-Tn7 integration into chromosome |
| Nsco_0193 | cacagcataactggactgatttc | Internal Tn7 right end primer for screening mini-Tn7 integration ^7^ |
| Nsco_0784 | cataagcctgttcggttcgt | Binds between Tn7L and Tn7R within mini-Tn7 element. Paired with Nsco_0158 or Nsco_0201 verifies the mini-Tn7 integration into chromosome |
| Nsco_0157 | cggtcgagttagcacaggat | *B. cenocepacia* *glmS1* (*BCAL0611*) Att site forward screening primers |
| Nsco_0158 | caactgctcgcgtatcacac | *B. cenocepacia* *glmS1* (*BCAL0611*) Att site reverse screening primers |
| Nsco_0200 | gtggagcaccatttcgtgag | *B. cenocepacia* *glmS2 (BCAM0478)* Att site forward screening primers |
| Nsco_0201 | tgataagccgaggaatttgg | *B. cenocepacia* *glmS2 (BCAM0478)* Att site reverse screening primers |
| 6135 | ggattcgacatgggtcaaag | Forward Screening primer for the Tmp^R^ cassette within mini-Tn7 |
| Nsco_0931 | gctcgaattagcttcaaaagcgc | Reverse Screening primer for the Tmp^R^ cassette within mini-Tn7 |
| Mutation construct (pGPI-SceI) construction and mutant screening | | |
| Nsco_0279 | gcatgcgatatcgagctctccctcttgaagccgttgtagtcg | Upstream *ogcX* forward primer for creation of pGPI-SceI-ogcX |
| Nsco_0280 | gtcatgttcgcagcttgaaattagatctgggtgagccgttc | Upstream *ogcX* reverse primer for creation of pGPI-SceI-ogcX |
| Nsco_0281 | gaacggctcacccagatctaatttcaagctgcgaacatgac | Downstream *ogcX* forward primer for creation of pGPI-SceI-ogcX |
| Nsco_0282 | cggataacaatttgtggaattcccgaaatgcagcaggttcttgg | Downstream *ogcX* reverse primer for creation of pGPI-SceI-ogcX |
| Nsco_0194 | gcatgcgatatcgagctctcccaatcctcgtcccgaccat | Upstream *ogcAB* forward primer for creation of pGPI-SceI-ogcAB |
| Nsco_0212 | cgatgcttccatcagcgtctagccggcatcagcacggccac | Upstream *ogcAB* reverse primer for creation of pGPI-SceI-ogcAB |
| Nsco_0213 | gtggccgtgctgatgccggctagacgctgatggaagcatcg | Downstream *ogcAB* forward primer for creation of pGPI-SceI-ogcAB |
| Nsco_0182 | gcatgcgatatcgagctctcccgtgcacgtgctgatcgtc | Upstream *ogcB* forward primer for creation of pGPI-SceI-ogcB |
| Nsco_0183 | cgatgcttccatcagcgtctaaggtggcggacatgaaaa | Upstream *ogcB* reverse primer for creation of pGPI-SceI-ogcB |
| Nsco_0184 | ttttcatgtccgccaccttagacgctgatggaagcatcg | Downstream *ogcB* forward primer for creation of pGPI-SceI-ogcB |
| Nsco_0185 | cggataacaatttgtggaattcccgcgatcagcttcgactgg | Downstream *ogcB* reverse primer for creation of pGPI-SceI-ogcB/pGPI-SceI-ogcAB |
| Nsco_0176 | gcatgcgatatcgagctctcccgttcgacaagccgctgtt | Upstream *ogcA* forward primer for creation of pGPI-SceI-ogcA |
| Nsco_0177 | gtcacgaaatgcagcaggtctagtcgtcgtgcccgttgtag | Upstream *ogcA* reverse primer for creation of pGPI-SceI-ogcA |
| Nsco_0178 | ctacaacgggcacgacgactagacctgctgcatttcgtgac | Downstream *ogcA* forward primer for creation of pGPI-SceI-ogcA |
| Nsco_0179 | cggataacaatttgtggaattcccgttcgagatcgacgccttc | Downstream *ogcA* reverse primer for creation of pGPI-SceI-ogcA |
| Nsco_0271 | gcatgcgatatcgagctctcccccgtgctcaaggtcatgc | Upstream *ogcI* forward primer for creation of pGPI-SceI-ogcI |
| Nsco_0272 | gtatgccgccagaacagcctatagcagggagacgatgaagc | Upstream *ogcI* reverse primer for creation of pGPI-SceI-ogcI |
| Nsco_0273 | gcttcatcgtctccctgctataggctgttctggcggcatac | Downstream *ogcI* forward primer for creation of pGPI-SceI-ogcI |
| Nsco_0274 | cggataacaatttgtggaattccccctgtacggtttctcggaag | Downstream *ogcI* reverse primer for creation of pGPI-SceI-ogcI |
| 6108 | taacggttgtggacaacaagccaggg | Forward primer for screening insertion into pGPI-SceI and integration of pGPI-SceI into the chromosomes ^11^ |
| 6109 | gccctacacaaattgggagatatatc | Reverse primer for screening insertion into pGPI-SceI and integration of pGPI-SceI into the chromosomes ^11^ |
| Nsco_0275 | tgactcgcatgatcgaactc | Forward primer to verify *ogcI* deletion |
| Nsco_0276 | cgtcacttcgtgctgatctc | Reverse primer to verify *ogcI* deletion |
| Nsco_0180 | atgcgaatacgcttctcctg | Forward primer to verify *ogcA* deletion |
| Nsco_0181 | agggtgataccggttaacga | Reverse primer to verify *ogcA* deletion |
| Nsco_0186 | tttcaagctgcgaacatgac | Forward primer to verify *ogcB* deletion |
| Nsco_0187 | atacggcatcaggttgttcg | Reverse primer to verify *ogcB* deletion |
| Nsco_0283 | gatctgctcgccgtagattg | Forward primer to verify *ogcX* deletion |
| Nsco_0284 | atcagcccgtggcgatac | Reverse primer to verify *ogcX* deletion |
| 6689 | accacgccacgaatgtcata | Forward primer to verify *ogc* deletion |
| 6690 | cgaacatcatgaagctgacc | Reverse primer to verify *ogc* deletion |
| Nsco_0780 | ggcaactatcgggcaaagtaccgc | Forward primer to verify *ogcA* deletion, binds internally within *ogcA* |
| Nsco_0781 | gcggtactttgcccgatagttgcc | Reverse primer to verify *ogcA* deletion, binds internally within *ogcA* |
| 7162 | gatctgctcgccgtagattg | Forward primer to verify the removal of the *ogc* |
| 7163 | gcgagaagctttacgaggaa | Reverse primer to verify the removal of the *ogc* |
| Construction of pSCrhaB2/pCumate expression vectors | | |
| Nsco_0216 | cgaattcaggcgctttttag | pSCrhaB2-ogc screening forward primer |
| Nsco_0217 | acggcgtttcacttctgagt | pSCrhaB2-ogc screening reverse primer |
| Nsco_0698 | ctttccctggttgccaatggccc | pSCrhaB2-ogc screening forward primer alternative 1 |
| Nsco_0699 | cggcgtttcacttctgagttcggc | pSCrhaB2-ogc screening reverse primer alternative 2 |
| Nsco_0777 | tgagcatcacatcaccacaattcagc | pSCrhaB2-ogc screening forward primer alternative 2 |
| Nsco_0778 | ccgccaggcaaattctgttttatcagac | pSCrhaB2-ogc screening reverse primer alternative 3 |
| Nsco_0526 | cgtaatgaaattcagcaggatcacatatgctcagcttcgcgtccgg | Forward primer containing *Nde*I for *ogcI* to insert into pSCrhaB2 |
| Nsco_0527 | tgcctgcaggtcgactctagagtatcgctcaacggctctgt | Reverse primer containing *Xba*I for *ogcI* to insert into pSCrhaB2 |
| Nsco_0566 | cgtaatgaaattcagcaggatcacatAtggccgtgctgatgccggcctacaacg | Forward primer containing *Nde*I to insert *ogcA* met1 for insertion into pSCrhaB2 |
| Nsco_0567 | cgtaatgaaattcagcaggatcacatatgacgtcccctgcttgcccgacc | Forward primer containing *Nde*I to insert *ogcA*-met2 for insertion into pSCrhaB2 |
| Nsco_0525 | tgcctgcaggtcgactctagagcgtcgaaattgccgaac | Reverse primer containing *Xba*I for amplification of *ogcA* and insertion into pSCrhaB2 |
| Nsco_0911 | cgtaatgaaattcagcaggatcacatatggctgctcgcgctggccatcg | Forward primer containing *Nde*I site upstream of the alterative start codon *of ogcA* for amplification of *ogcAB* and insertion into pSCrhaB2 |
| Nsco_0214 | aaaaacatatgtccgccacctccccgctgcgc | Forward primer containing *Nde*I site for amplification of *ogcB* and insertion into pSCrhaB2 |
| Nsco_0215 | aaaaatctagagctgatgcgctccttcagagg | Reverse primer containing *Xba*I for amplification of *ogcB* and insertion into pSCrhaB2 |
| Nsco_0919 | gaaattcagcaggatcacatatgctgaagcgcttcggcaacccgg | Forward primer for amplification of the *ogc* cluster for insertion into pSCrhaB2 |
| Nsco_0920 | gcatgcctgcaggtcgactctaggggtgacgtggctccaggccggaac | Reverse primer for amplification of the *ogc* cluster for insertion into pSCrhaB2 |
| Nsco_1092 | ctgaaatttgcttcggggtcattatagggtctgataaaacagaatttgcctgg | Forward primer to amplify the pMLBAD vector and allow integration of CymR- sfGFP |
| Nsco_1093 | cctttttctttaaaaccgaaaagattaccgatgggagatcctaagatatcgc | Reverse primer to amplify the pMLBAD vector and allow integration of CymR- sfGFP |
| Nsco_1094 | ccaggcaaattctgttttatcagaccctataatgaccccgaagcaaatttcag | Forward primer to amplify CymR-sfGFP for insertion into pMLBAD |
| Nsco_1095 | gcgatatcttaggatctcccatcggtaatcttttcggttttaaagaaaaagg | Reverse primer to amplify CymR-sfGFP for insertion into pMLBAD |
| Nsco_1455 | ccgggttgccgaagcgcttcagcattatcttacctccttaatttgatttc | Forward primer to amplify pCumate vector backbone and the insertion *ogcX* |
| Nsco_1457 | ttacatccgtttcaagctgcgaacacaccaccaccaccaccactgatgatactagtagcggccgctgcagc | Forward primer to amplify pCumate vector backbone and the insertion *ogcX*-His_6_ |
| Nsco_1453 | gaaatcaaattaaggaggtaagataatgctgaagcgcttcggcaacccgg | Forward primer to amplify ogcX for insertion into pCumate |
| Nsco_1458 | gctgcagcggccgctactagtatcatcagtggtggtggtggtggtgtgttcgcagcttgaaacggatgtaa | Reverse primer to amplify *ogc*X with a His_6_-tag for insertion into pCumate |
| Nsco_1590 | cggtagagctggtataggtcattatcttacctccttaatttgatttc | Forward primer to amplify pCumate vector backbone and the insertion of BCAL2087 (UppS1) |
| Nsco_1591 | gcagaacgccgactccctttcatgctgatgatactagtagcggccgctgcagc | Reverse primer to amplify pCumate vector backbone and the insertion of BCAL2087 (UppS1) |
| Nsco_1588 | gaaatcaaattaaggaggtaagataatgacctataccagctctaccg | Forward primer to amplify BCAL2087 (UppS1) for insertion into pCumate |
| Nsco_1589 | gctgcagcggccgctactagtatcatcagcatgaaagggagtcggcgttctgc | Reverse primer to amplify BCAL2087 (UppS1) for insertion into pCumate |
| Nsco_1419 | cattatcttacctccttaatttgatttc | Forward primer to amplify pCumate vector backbone and the insertion of BCAM2067 (UppS2) |
| Nsco_1420 | tgatactagtagcggccgctgcagca | Reverse primer to amplify pCumate vector backbone and the insertion of BCAM2067 (UppS2) |
| Nsco_1594 | gaaatcaaattaaggaggtaagataatgactcaagagctgattctgcgcg | Forward primer to amplify BCAM2067 (UppS2) for insertion into pCumate |
| Nsco_1662 | gctgcagcggccgctactagtatcaaacgggtgtcatgaaggacggtcc | Reverse primer to amplify BCAM2067 (UppS2) for insertion into pCumate |
| Nsco_1502 | cccagaatgttaccatcctctt | Forward screening primer to verify insertion of sfGFP into pCumate |
| Nsco_1256 | agatctgccatgagacccaa | Reverse screening primer to verify insertion of sfGFP into pCumate |
| Nsco_1473 | ttcggtgatctgttcgtaaagc | Forward sequencing primer to verify insertion into pCumate |
| Nsco_1308 | gccttgaccgaaacggaggaat | Reverse sequencing primer to verify insertion into pCumate |
| pSCrhaB2 mutagenesis | | |
| Nsco_1186 | cgtaatgaaattcagcaggatcacattgacgtcccctgcttgcccgaccccgc | Forward primer for deletion of *ogcX* from pSCrhaB2-*ogc* |
| Nsco_1187 | gcggggtcgggcaagcaggggacgtcaatgtgatcctgctgaatttcattacg | Reverse primer for deletion of *ogcX* from pSCrhaB2-*ogc* |
| Nsco_1188 | cgctcgacgacgtggccgtgctgtgacgtgcatccggcccgttcggcaatttcg | Forward primer for deletion of *ogcA* from pSCrhaB2-*ogc* |
| Nsco_1189 | cgaaattgccgaacgggccggatgcacgtcacagcacggccacgtcgtcgagcg | Reverse primer for deletion of *ogcA* from pSCrhaB2-*ogc* |
| Nsco_1190 | cgacgctttattttttctttttttcggaattcgacgaacagcaggtcgtcgaacg | Forward primer for deletion of *ogcB* from pSCrhaB2-*ogc* |
| Nsco_1191 | cgttcgacgacctgctgttcgtcgaattccgaaaaaaagaaaaaataaagcgtcg | Reverse primer for deletion of *ogc*B from pSCrhaB2-*ogc* |
| Nsco_1192 | ccgctgtccattttccgagcggtccctgagcgatacgctcgcttccgaacaaaaaag | Forward primer for deletion of *ogcI* from pSCrhaB2-*ogc* |
| Nsco_1193 | cttttttgttcggaagcgagcgtatcgctcagggaccgctcggaaaatggacagcgg | Reverse primer for deletion of *ogc*I from pSCrhaB2-*ogc* |

**Supplementary Table 4: Proteomic Dataset**

| **Pride accession number (Review login details)** | **MS instrument** | **Number of Biological groups, replicates and total datafiles** | **Description of dataset** |
| --- | --- | --- | --- |
| PXD054841  Username: reviewer_pxd054841@ebi.ac.uk Password: vtOYlh2QeMmH | Orbitrap Fusion Lumos | 2 biological groups, 4 replicates total of 8 datafiles | DDA experiment assessing glycosylation and expression of OgcB from pSCrhaB2-OgcB or pSCrhaB2 within K56-2 Δ*ogc*B at stationary phase within LB with 0.05% rhamnose. |
| PXD054867  Username: reviewer_pxd054867@ebi.ac.uk Password: 1KCRw6fckXC0 | Orbitrap Fusion Lumos | 2 biological groups, 4 replicates total of 8 datafiles | DDA experiment assessing glycosylation and expression of OgcI from pSCrhaB2-OgcI or pSCrhaB2 within K56-2 Δ*ogcI* at stationary phase within LB with 0.05% rhamnose. |
| PXD055163  Username: reviewer_pxd055163@ebi.ac.uk Password: xQ0vECsjoX74 | Orbitrap Fusion Lumos | 6 biological groups, 4 replicates total of 24 datafiles | DDA experiment assessing glycosylation and expression of OgcAB from pSCrhaB2-OgcAB_Met1_;  pSCrhaB2-OgcAB_Met2_ or pSCrhaB2 within K56-2 Δ*ogcAB* at stationary phase within LB with 1% rhamnose. |
| PXD054929  Username: reviewer_pxd054929@ebi.ac.uk Password: QFVtjaWskLkP | Orbitrap Fusion Lumos equipped with a FAIMS Pro interface | 8 biological groups, 4 replicates total of 32 datafiles | DDA experiment assessing glycosylation within K56-2 Δ*ogc* and K56-2 WT containing pSCrhaB2-*ogc* or pSCrhaB2 at stationary phase within LB with 1% rhamnose. |
| PXD054956  Username: reviewer_pxd054956@ebi.ac.uk Password: irFS6N8GHplR | Orbitrap Fusion Lumos | 7 biological groups, 4 replicates total of 28 datafiles | DDA experiment assessing glycosylation and expression of *ogcA* from pSCrhaB2-*ogcA*_Met1_; pSCrhaB2-*ogcA*_Met2_; within K56-2 Δ*ogcI*Δ*ogcA* Tn7-*ogc*I and K56-2 Δ*ogcI* Tn7-*ogc*I at stationary phase within LB with 1% rhamnose with pSCrhaB2 containing strains used as negative controls. |
| PXD056440  **Username:** reviewer_pxd056440@ebi.ac.uk  **Password:** 4e7AjHcW3n8v | Orbitrap Fusion Lumos | 10 biological groups, 4 replicates total of 40 datafiles | DDA experiment assessing glycosylation and expression of *ogcX* from pCumate-*ogcX-his* within K56-2 Δ*ogcI*Δ*ogcX* Tn7-*ogc*I and K56-2 Δ*ogcI* Tn7-*ogc*I at stationary phase within LB with 1% rhamnose/100 μM cumate with  pCumate-sfGFP containing strains used as negative controls. |
| PXD054923  Username: reviewer_pxd054923@ebi.ac.uk Password: ZiiuDcoa2nre | Orbitrap Fusion Lumos equipped with a FAIMS Pro interface | 6 biological groups, 4 replicates total of 24 datafiles | DDA experiment assessing glycosylation at stationary phase within LB of K56-2 Δ*ogcI* Tn7-*ogc*I (non-induced) compared to K56-2 Δ*ogcI* Tn7-*ogc*I; K56-2 Δ*ogc*IΔ*ogcA* Tn7-*ogc*I; K56-2 Δ*ogcI*Δ*ogcAB* Tn7-*ogc*I; K56-2 Δ*ogcI*Δ*ogcB* Tn7-*ogc*I and K56-2 Δ*ogcI*Δ*ogcX* Tn7-*ogcI* induced with 0.1% rhamnose. |
| PXD054949  Username: reviewer_pxd054949@ebi.ac.uk Password: imU103bFkKkO | Orbitrap Fusion Lumos | 8 biological groups, 4 replicates total of 32 datafiles | DIA experiment assessing proteome changes at stationary phase within LB of K56-2 Δ*ogcI* Tn7-*ogcI*; K56-2 Δ*ogcI*Δ*ogcA* Tn7-*ogcI*; K56-2 Δ*ogcI*Δ*ogcB* Tn7-*ogc*I and K56-2 Δ*ogcI*Δ*ogcX* Tn7-*ogc*I (non-induced) compared to K56-2 Δ*ogc*I Tn7-*ogcI*; K56-2 Δ*ogc*IΔ*ogcA* Tn7-*ogcI*; K56-2 Δ*ogc*IΔ*ogcB* Tn7-*ogc*I and K56-2 Δ*ogcI*Δ*ogcX* Tn7-*ogcI* induced with 0.1% rhamnose. |
| PXD055576  Username: reviewer_pxd055576@ebi.ac.uk Password: PLRffXCkJY41 | Orbitrap Fusion Lumos equipped with a FAIMS Pro interface | 3 biological groups, 4 replicates total of 12 datafiles | DDA experiment assessing glycosylation at stationary phase within LB of K56-2 Δ*ogcI* Tn7-*ogcI* (non-induced) compared to K56-2 Δ*ogcI* Tn7-*ogcI* with 0.05% and 0.1% rhamnose. |
| PXD059283  Username: reviewer_pxd059283@ebi.ac.uk Password: 8F2GdarAPyoS | Orbitrap Fusion Lumos | 8 biological groups, 4 replicates total of 32 datafiles | DDA experiment assessing the impact of OgcX overexpression of pCumate-*ogcX-his* on glycosylation glycan patterns within  K56-2 Δ*ogcI* Tn7-*ogcI*; K56-2 Δ*ogcI*Δ*ogcA* Tn7-*ogcI*; K56-2 Δ*ogcI*Δ*ogcB* Tn7-*ogc*I and K56-2 Δ*ogcI*Δ*ogcX* Tn7-*ogc*I  at stationary phase in LB with and without 1% rhamnose/100 μM cumate. |

**Supplementary Table 5: Antimicrobial Minimal inhibitory concentrations (MIC).**

| **Strains** | **Rhamnose** | **Tetracycline** | **Trimethoprim** | **Rifampicin** | **Ceftazidime** | **Chlorhexidine** |
| --- | --- | --- | --- | --- | --- | --- |
| Δ*ogcI* Tn7-*ogcI* | 0% | 32-64 | 16 | 64 | 32 | 16-32 |
| Δ*ogcI* Tn7-*ogcI* | 1% | 32-64 | 32 | 64 | 32 | 16-32 |
| Δ*ogcI*Δ*ogcX* Tn7-*ogcI* | 0% | 32-64 | 16 | 64 | 32 | 16-32 |
| Δ*ogcI*Δ*ogcX* Tn7-*ogcI* | 1% | 32-64 | 16 | 64 | 4 | 16-32 |
| Δ*ogcI*Δ*ogcA* Tn7-*ogcI* | 0% | 32-64 | 16 | 16-64 | 64 | 16-32 |
| Δ*ogcI*Δ*ogcA* Tn7-*ogcI* | 1% | 32-64 | 16 | 64 | 4 | 16-32 |
| Δ*ogcI*Δ*ogcB* Tn7-*ogcI* | 0% | 32-64 | 16 | 64 | 32 | 16-32 |
| Δ*ogcI*Δ*ogcB* Tn7-*ogcI* | 1% | 32-64 | 32 | 64 | 64 | 16-32 |

The antimicrobial susceptibility of bacterial strains was determined via broth dilution methods in accordance with Clinical and Laboratory Standards Institute (CLSI) guidelines. Strains were cultured in LB broth to an OD_600_ of 0.5–0.6, followed by a 1:100 dilution in cation-adjusted Mueller–Hinton broth (CAMHB) with and without the addition of rhamnose. Serial twofold dilutions of antibiotics—trimethoprim, tetracycline, chlorhexidine, ceftazidime, and rifampicin—were prepared in CAMHB within flat-bottom microplates. Bacterial inoculum was added to each well, with one well per plate reserved as a growth control (no antibiotics) and another as a sterility control (no inoculum). Plates were incubated at 37°C for 20 hours. Post-incubation, microbial growth was assessed visually, and optical density was measured at 600 nm to quantify bacterial growth.

**Supplementary Proteomics tables**

**Supplementary Table 6. DDA peptide-spectrum matches (PSMs) summary of *B. cenocepacia* WT and Δ*ogc* containing pSCrhaB2 and pSCrhaB2-*ogc****.* The MSfragger PSM search summary for the proteome analysis of *B. cenocepacia* WT and Δ*ogc* strains containing pSCrhaB2 and pSCrhaB2-*ogc* are provided. For each modification type identified within the proteome the total number of PSMs assigned are tabulated.

**Supplementary Table 7. DDA protein level LFQ analysis of *B. cenocepacia* WT and Δ*ogc* containing pSCrhaB2 and pSCrhaB2-*ogc*.** The Perseus processed MSfragger search results for the protein analysis of four biological replicates of strains WT and Δ*ogc* containing the plasmids pSCrhaB2 and pSCrhaB2-*ogc* are provided. For each identified protein, the log2 LFQ protein values, the T-test information including the -log_10_(*p*-value), differences in the mean between the groups and if the resulting *p*-values is below the multiple hypothesis corrected *p*-value are provided. For each protein the top peptide probability, total number of MS/MS events for the corresponding protein as well as both the imputed and non-imputed data is provided.

**Supplementary Table 8. DDA PSM summary of *B. cenocepacia* Δ*ogcI* Tn7-*ogcI* strains with and without induction*.*** The MSfragger PSMs search summary for the proteome analysis of *B. cenocepacia* Δ*ogcI* Tn7-*ogcI* with and without induction for *B. cenocepacia* Δ*ogcI*Δ*ogcX* Tn7-*ogcI, B. cenocepacia* Δ*ogcI*Δ*ogcA* Tn7-*ogcI, B. cenocepacia* Δ*ogcI*Δ*ogcB* Tn7-*ogcI and B. cenocepacia* Δ*ogcI*Δ*ogcAB* Tn7-*ogcI* is provided. For each modification type identified within the proteome the total number of PSMs assigned are tabulated.

**Supplementary Table 9. DDA protein level LFQ analysis of *B. cenocepacia* Δ*ogcI* Tn7-*ogcI* and *B. cenocepacia* Δ*ogcI*Δ*ogcA* Tn7-*ogcI* containing pSCrhaB2, pSCrhaB2-*ogcA*_Met1_, or pSCrhaB2-*ogcA*_Met2_.** The Perseus processed MSfragger search results for the protein analysis of four biological replicates of strains *B. cenocepacia* Δ*ogcI* Tn7-*ogcI* and *B. cenocepacia* Δ*ogcI*Δ*ogcA* Tn7-*ogcI* containing the plasmids pSCrhaB2, pSCrhaB2-*ogcA*_Met1_, or pSCrhaB2-*ogcA* _Met2_without and with 1% rhamnose (with the exception of *B. cenocepacia* Δ*ogcI*Δ*ogcA* Tn7-*ogcI* pSCrhaB2 due to lack of viability). For each identified protein, the log2 LFQ protein values, the T-test information including the -log_10_(*p*-value), differences in the mean between the groups and if the resulting *p*-values is below the multiple hypothesis corrected *p*-value are provided. For each protein the top peptide probability, total number of MS/MS events for the corresponding protein as well as both the imputed and non-imputed data is provided.

**Supplementary Table 10. DDA PSM summary of *B. cenocepacia* Δ*ogcI* Tn7-*ogcI* and *B. cenocepacia* Δ*ogcI*Δ*ogcA* Tn7-*ogcI* containing pSCrhaB2, pSCrhaB2-*ogcA*_Met1_, or pSCrhaB2-*ogcA*_Met2_ strains with and without induction*.*** The MSfragger PSM search summary for the proteome analysis of *B. cenocepacia* Δ*ogcI* Tn7-*ogcI* and *B. cenocepacia* Δ*ogcI*Δ*ogcA* Tn7-*ogcI* containing pSCrhaB2, pSCrhaB2-*ogcA*_Met1_, or pSCrhaB2-*ogcA*_Met2_ strains is provided. For each modification type identified within the proteome the total number of PSMs assigned are tabulated.

**Supplementary Table 11. DDA protein level LFQ analysis of *B. cenocepacia* Δ*ogcI* Tn7-*ogcI* and *B. cenocepacia* Δ*ogcI*Δ*ogcX* Tn7-*ogcI* containing pCumate-sfGFP and pCumate-*ogcX-his*.** The Perseus processed MSfragger search results for the protein analysis of four biological replicates of strains *B. cenocepacia* Δ*ogcI* Tn7-*ogcI* and *B. cenocepacia* Δ*ogcI*Δ*ogcX* Tn7-*ogcI* containing the plasmids pCumate-sfGFP and pCumate-*ogcX-his* without and with 1% rhamnose/100 μM cumate. For each identified protein, the log2 LFQ protein values, the T-test information including the -log_10_(*p*-value), differences in the mean between the groups and if the resulting *p*-values is below the multiple hypothesis corrected *p*-value are provided. For each protein the top peptide probability, total number of MS/MS events for the corresponding protein as well as both the imputed and non-imputed data is provided.

**Supplementary Table 12. DDA PSM summary of *B. cenocepacia* Δ*ogcI* Tn7-*ogcI* and *B. cenocepacia* Δ*ogcI*Δ*ogcX* Tn7-*ogcI* containing pCumate-sfGFP and pCumate-*ogcX-his* with and without induction*.*** The MSfragger PSMs search summary for the proteome analysis of *B. cenocepacia* Δ*ogcI* Tn7-*ogcI* and *B. cenocepacia* Δ*ogcI*Δ*ogcX* Tn7-*ogcI* containing pCumate-sfGFP and pCumate-*ogcX-his* strains are provided. For each modification type identified within the proteome the total number of PSMs assigned are tabulated.

**Supplementary Table 13. DIA Proteomic analysis of Δ*ogcI* Tn7-ogcI, Δ*ogcI*Δ*ogcA* Tn7-*ogcI*, Δ*ogcI*Δ*ogcB* Tn7-*ogcI*, and Δ*ogcI*Δ*ogcX* Tn7-*ogcI* strains with and without rhamnose induction (1% rhamnose).** The Spectronaut search results for protein level analysis of biological replicates (n=4) grown +/- the addition of Rhamnose of Δ*ogcI* Tn7-ogcI, Δ*ogcI*Δ*ogcA* Tn7-*ogcI*, Δ*ogcI*Δ*ogcB* Tn7-*ogcI*, and Δ*ogcI*Δ*ogcX* Tn7-*ogcI*. Imputed and non-imputed Perseus processed data with statistical analysis is provided. For each identified protein, the log_2_ LFQ protein values, T-‑test information including the -log_10_(*p*-value), difference in the mean between the groups and if the resulting *p*-values are below 0.05 and the multiple hypothesis corrected *p*‑values (permutation-based false discovery rate of 0.05) are provided. Categorical information associated with protein accessions, gene name, and GO terms are provided in addition to if proteins were identified by a single PSM within a given experiment as well as the total number of precursors assigned for each protein within each replicate.

**Supplementary Table 14. Enrichment analysis of proteome changes observed within Δ*ogcI*Δ*ogcX* Tn7-*ogcI* and Δ*ogcI*Δ*ogcA* Tn7-*ogcI.*** Fisher exact tests outputs assessing the co-occurrence of categorical assignments (statistically significant changes to proteins observed based on GO terms).

**Supplementary Table 15. DDA protein level LFQ analysis of *B. cenocepacia* Δ*ogcI* Tn7-*ogcI* strains containing p*ogcX* with and without induction.** The Perseus processed MSfragger search results for the protein analysis of four biological replicates of *B. cenocepacia* Δ*ogcI* Tn7-*ogcI,* Δ*ogcI*Δ*ogcX* Tn7-*ogcI, B. cenocepacia* Δ*ogcI*Δ*ogcA* Tn7-*ogcI and B. cenocepacia* Δ*ogcI*Δ*ogcB* Tn7-*ogcI* containing pCumate-*ogcX-his* with and without induction (1% Rhamnose, 100 μM cumate) are provided. For each identified protein, the log2 LFQ protein values, the ANOVA test including -log_10_(*p*-value) and if the proteins with *p*-values below the multiple hypothesis corrected *p*-value are provided. For each protein the top peptide probability, total number of MS/MS events for the corresponding protein as well as both the imputed and non-imputed data is provided.

**Supplementary Table 16. DDA PSM summary of *B. cenocepacia* Δ*ogcI* Tn7-*ogcI* strains containing p*ogcX* with and without induction*.*** The MSfragger PSMs search summary for the proteome analysis of *B. cenocepacia* Δ*ogcI* Tn7-*ogcI,* Δ*ogcI*Δ*ogcX* Tn7-*ogcI, B. cenocepacia* Δ*ogcI*Δ*ogcA* Tn7-*ogcI and B. cenocepacia* Δ*ogcI*Δ*ogcB* Tn7-*ogcI* containing pCumate-*ogcX-his* with and without induction is provided. For each modification type identified within the proteome the total number of PSMs assigned are tabulated.

**Supplementary Table 17. DDA protein level LFQ analysis of *B. cenocepacia* Δ*ogcAB* containing pSCrhaB2, pSCrhaB2-*ogcAB*_Met1_, or pSCrhaB2-*ogcAB*_Met2_.** The Perseus processed MSfragger search results for the protein analysis of four biological replicates of strains *B. cenocepacia* Δ*ogcAB* containing the plasmids pSCrhaB2, pSCrhaB2-*ogcAB*-Met1, or pSCrhaB2-*ogcAB*-Met2 without and with induction. For each identified protein, the log2 LFQ protein values, the T-test information including the -log_10_(*p*-value), differences in the mean between the groups and if the resulting *p*-values is below the multiple hypothesis corrected *p*-value are provided. For each protein the top peptide probability, total number of MS/MS events for the corresponding protein as well as both the imputed and non-imputed data is provided.

**Supplementary Table 18. DDA PSMs summary of *B. cenocepacia* Δ*ogcAB* containing pSCrhaB2, pSCrhaB2-*ogcAB*_Met1_, or pSCrhaB2-*ogcAB*_Met2_ with and without induction*.*** The MSfragger PSM search summary for the whole proteome analysis of *B. cenocepacia* Δ*ogcI* Δ*ogcAB* Tn7-*ogcI* containing pSCrhaB2, pSCrhaB2-*ogcAB*_Met1_, or pSCrhaB2-*ogcAB*_Met2_ strains are provided. For each modification type identified within the proteome the total number of PSMs assigned are tabulated.

**Supplementary Table 19. DDA protein level LFQ analysis of *B. cenocepacia* Δ*ogcB* containing pSCrhaB2 and pSCrhaB2-*ogcB*.** The Perseus processed MSfragger search results for the protein analysis of four biological replicates of strains *B. cenocepacia* Δ*ogcB* containing the plasmids pSCrhaB2 and pSCrhaB2-*ogcB* grown with 0.05% rhamnose induction. For each identified protein, the log2 LFQ protein values, the T-test information including the -log_10_(*p*-value), differences in the mean between the groups and if the resulting *p*-values is below the multiple hypothesis corrected *p*-value are provided. For each protein the top peptide probability, total number of MS/MS events for the corresponding protein as well as both the imputed and non-imputed data is provided.

**Supplementary Table 20. DDA PSM summary of *B. cenocepacia* Δ*ogcB* containing pSCrhaB2 and pSCrhaB2-*ogcB* with induction*.*** The MSfragger PSM search summary for proteome analysis of *B. cenocepacia* Δ*ogcB* containing pSCrhaB2 and pSCrhaB2-*ogcB* strains are provided. For each modification type identified within the proteome the total number of PSMs assigned are tabulated.

**Supplementary Table 21. DDA protein level LFQ analysis of *B. cenocepacia* Δ*ogcI* containing pSCrhaB2 and pSCrhaB2-*ogcI*.** The Perseus processed MSfragger search results for the protein analysis of four biological replicates of strains *B. cenocepacia* Δ*ogcI* containing the plasmids pSCrhaB2 and pSCrhaB2-*ogcI* grown with 0.05% rhamnose induction. For each identified protein, the log2 LFQ protein values, the T-test information including the -log_10_(*p*-value), differences in the mean between the groups and if the resulting *p*-values is below the multiple hypothesis corrected *p*-value are provided. For each protein the top peptide probability, total number of MS/MS events for the corresponding protein as well as both the imputed and non-imputed data is provided.

**Supplementary Table 22. DDA PSM summary of *B. cenocepacia* Δ*ogcI* containing pSCrhaB2 and pSCrhaB2-*ogcI* with induction*.*** The MSfragger PSM search summary for the proteome analysis of *B. cenocepacia* Δ*ogcI* containing pSCrhaB2 and pSCrhaB2-*ogcI* strains are provided. For each modification type identified within the proteome the total number of PSMs assigned are tabulated.

**Supplementary Methods**

**Preparation of proteomic samples:** *B. cenocepacia* cultures for proteomic analysis were grown overnight with or without induction with rhamnose and cumate as outlined above with shaking at 180 rpm. Overnight cultures were normalised to an OD_600_ of 1.0 and then collected by centrifugation at 10,000 x *g* at 4˚C for 10 minutes, washed 3 times with ice-cold PBS and then snap frozen at -80˚C until processing. Frozen whole cell samples were prepared for analysis using sodium deoxycholate (SDC) based lysis and the in-StageTip preparation approach as previously described ^12^. OgcX complementation assays were undertaken using SDS-based lysis and S-trap sample preparation to improve the detection of OgcX. For in-StageTip preparation, cells were resuspended in 4% SDC, 100 mM Tris pH 8.0 and boiled at 95˚C with shaking (2000 rpm) for 10 minutes to solubilise the proteome. Samples were allowed to cool for 10 minutes and then boiled for a further 10 minutes (95°C, 2000 rpm) before the protein concentrations were determined by bicinchoninic acid assays (Thermo Fisher Scientific). Samples were reduced/alkylated with the addition of Tris-2-carboxyethyl phosphine and chloroacetamide (final concentration 10 mM and 40 mM, respectively), and samples were incubated in the dark for 0.5-1 hour at 45˚C. Following reduction/alkylation, samples were digested overnight with Trypsin (1/50 w/w Solu-trypsin, Sigma) at 37˚C with shaking at 1000rpm. Digests were then quenched with the addition of 1.25 volumes of isopropanol before being cleaned up using SDB-RPS (Sigma) StageTips.

For S-trap-based preparation, cells were resuspended in 4% sodium dodecyl sulphate (SDS), 100 mM Tris pH 8.0, and boiled at 95˚C with shaking (2000 rpm) for 10 minutes; the protein concentrations were then determined by bicinchoninic acid assays, and samples were reduced/alkylated as above. Samples were acidified to 1.2% phosphoric acid and diluted with seven volumes of S-trap wash buffer (90% methanol, 100mM tetraethylammonium bromide pH 7.1) before being loaded onto S-trap mini spin columns (Protifi) and washed 3 times with 400 µL of S-trap wash buffer. Samples were then digested with 2 µg of Trypsin (a 1:50 protease/protein ratio) in 100 mM tetraethylammonium bromide overnight at 37 °C before being collected by centrifugation with washes of 100 mM tetraethylammonium bromide, followed by 0.2% formic acid followed by 0.2% formic acid / 50% acetonitrile before being dried by vacuum centrifugation at room temperature and stored at -20˚C.

SDB-RPS StageTip-based clean-up of digests was undertaken by adjusting or resuspending samples in 50% isopropanol, 1% trifluoroacetic acid and then loading them on in-house created SDB-RPS StageTips, which were prepared according to previously described protocols ^12-14^. Briefly, five frits of SDB-RPS were excised using a blunt 16-gauge Hamilton needle and loaded into 200 μL tips. SDB-RPS StageTips were placed in a Spin96 tip holder ^13^ to enable batch-based spinning of samples and tips conditioned with 100% acetonitrile, followed by 30% methanol, 1% trifluoroacetic acid, followed by 90% isopropanol, 1% trifluoroacetic acid with each wash spun through the column at 1000 x *g* for 3 minutes. Acidified isopropanol/peptide mixtures were loaded onto the SDB-RPS columns and spun through tips before being washed with 90% isopropanol, 1% trifluoroacetic acid, followed by 90% ethyl acetate, 1% trifluoroacetic acid, followed by 1% trifluoroacetic acid in Milli-Q water. Peptide samples were eluted with 80% acetonitrile, 5% ammonium hydroxide and dried by vacuum centrifugation at room temperature and stored at -20˚C.

**LC-MS analysis of DDA samples.** Cleaned-up peptide samples were re-suspended in Buffer A* (2% acetonitrile, 0.1% trifluoroacetic acid in Milli-Q water) and separated using a two-column chromatography set-up on a Dionex Ultimate 3000 UPLC composed of a PepMap100 C18 20 mm x 75 μm trap and a PepMap C18 500 mm x 75 μm analytical column (Thermo Fisher Scientific) coupled to a Orbitrap Fusion™ Lumos™ Tribrid™ Mass Spectrometer (Thermo Fisher Scientific) with datasets collected with and without the use of the FAIMS Pro interface (Thermo Fisher Scientific). 145-minute gradients were run for each sample, with samples loaded onto the trap column with 98% Buffer A (2% acetonitrile, 0.1% formic acid in Milli-Q water) and 2% Buffer B (80% acetonitrile, 0.1% formic acid) with peptides separated by altering the buffer composition from 2% Buffer B to 28% B over 126 minutes, then from 28% B to 40% B over 9 minutes, then from 40% B to 80% B over 3 minutes, the composition was held at 80% B for 2 minutes, and then dropped to 2% B over 2 minutes and held at 2% B for another 3 minutes. For datasets collected without the use of the FAIMS Pro interface, the Lumos™ Mass Spectrometer was operated in a data-dependent mode, switching between the collection of a single Orbitrap MS scan (450-2000 m/z, maximal injection time of 50 ms, an Automatic Gain Control (AGC) of maximum of 4*10^5^ ions and a resolution of 60k) acquired every 3 seconds followed by Orbitrap MS/MS HCD scans of precursors (NCE 30%, maximal injection time of 80 ms, an AGC set to a maximum of 1.25*10^5^ ions and a resolution of 30k). For datasets collected with the FAIMS Pro interface, a data-dependent stepped FAIMS approach was utilised with three different FAIMS CVs -25, -45 and -65, as previously described ^15^. For each FAIMS CV a single Orbitrap MS scan (450-2000 m/z, maximal injection time of 50 ms, an AGC of maximum of 4*10^5^ ions and a resolution of 60k) was acquired every 1.2 seconds followed by Orbitrap MS/MS HCD scans of precursors (NCE 30%, maximal injection time of 80 ms, an AGC set to a maximum of 1.25*10^5^ ions and a resolution of 30k). HCD scans containing HexNAc-associated oxonium ions (204.0867, 138.0545 and 366.1396 m/z) triggered two additional product-dependent MS/MS scans ^16^ of potential glycopeptides; an Orbitrap EThcD scan (NCE 15%, maximal injection time of 150 ms, AGC set to a maximum of 2*10^5^ ions with a resolution of 30k using the extended mass range setting to improve the detection of high mass glycopeptide fragment ions ^17^) and a stepped collision energy HCD scan (using NCE 35% with 5% Stepping, maximal injection time of 150 ms, an AGC set to a maximum of 2*10^5^ ions and a resolution of 30k).

**LC-MS analysis of DIA samples:** Cleaned-up peptide samples were resuspended in Buffer A* and separated using a two-column chromatography set-up on a Dionex Ultimate 3000 UPLC composed of a PepMap100 C18 20 mm x 75 μm trap and a PepMap C18 500 mm x 75 μm analytical column. Samples were concentrated onto the trap column at 5 μL/min for 5 minutes with 98% Buffer A and 2% Buffer B, then infused into an Orbitrap Fusion™ Lumos™ Tribrid™ Mass Spectrometer at 300 nL/minute via the analytical column. 125-minute analytical runs were undertaken by altering the buffer composition from 3% Buffer B to 25% B over 112 minutes, then from 25% B to 40% B over 4 minutes, then from 40% B to 80% B over 1 minute. The composition was held at 80% B for 3 minutes and then dropped to 3% B over 1 minute before being held at 3% B for another 4 minutes. Data was collected in a Data-independent manner with a single MS1 event (120k resolution, AGC 1*10^6^, 350-1400 m/z) and 50 MS2 scans (NCE 30%, 30k resolution, AGC 1*10^6^, 200-2000 m/z and a maximal injection time of 55 ms) of a width of 13.7 m/z collected over the mass range of 360 to 1033.5 m/z.

**DDA-based proteomic analysis:** DDA datasets were analysed using MSFragger (versions 18.0, 19.0, 20.0 or 22.0) ^18-20^. Samples were searched with a Tryptic specificity, allowing a maximum of two missed cleavage events and Carbamidomethyl set as a fixed modification of Cysteine while oxidation of Methionine allowed as a variable modification. For searches to identify canonical Bukrholderia glycosylation the *Burkholderia* glycans HexHexNAc_2_ (elemental composition: C_22_O_15_H_36_N_2_, mass: 568.2115 Da) and Suc-HexHexNAc_2_ (elemental composition: C_26_O_18_H_40_N_2_, mass: 668.2276 Da) were included as variable modifications at Serine in line with the strong preference for PglL glycosylation at Serine residues ^21^. To allow the assessment of glycoforms within strains lacking *ogc* genes the *Burkholderia* glycans HexHexNAc_2_, Suc-HexHexNAc_2_, HexNAc_2_ (elemental composition: C_16_O_10_H_26_N_2_, mass: 406.1488 Da) and HexNAc (elemental composition: C_8_O_5_H_13_N, mass: 203.0794 Da) were included as variable modifications at Serine. For all glycopeptide searches the glycan fragment ions were defined as 204.0866, 186.0760 168.0655, 366.1395, 144.0656, 138.0550, 466.1555 and 407.1594. A maximum mass precursor tolerance of 20 ppm was allowed at both the MS1 and MS2 levels. Samples were searched against the *B. cenocepacia* reference proteome J2315 (Uniprot accession: UP000001035, 6,993 proteins, downloaded July 24^th^ 2020) ^22^ supplemented with the proteins RhaS (HTH-type transcriptional activator RhaS, P09377), RhaR (HTH-type transcriptional activator RhaR, P09378), TmpR (Dihydrofolate reductase resistance marker, P00384) and CymR (HTH-type transcriptional regulator CymR, O33453). Assessments of changes in glycosylation were undertaken based on spectral counting of glycopeptide identification events with data visualization undertaken using ggplot2 ^23^ in R. To confirm the identity of glycoforms representative HCD and EThcD spectra were annotated with the aid of the Interactive Peptide Spectral Annotator tool (<http://www.interactivepeptidespectralannotator.com/PeptideAnnotator.html>) ^24^.

**DIA-based proteomic analysis:** DIA Datasets were searched using DIA-library free analysis within Spectronaut (version 17.6). Data files were searched against the *B. cenocepacia* K56-2 proteome (Uniprot accession: UP000011196, 7467 proteins, downloaded October 13^th^ 2020) ^25^ and *B. cenocepacia* strain J2315 (Uniprot accession: UP000001035) ^22^, merging these proteomes enabling the matching of proteins to both J2315 and K56-2 accessions. Oxidation of Methionine was allowed as a variable modification, Carbamidomethyl was set as a fixed modification of Cysteine, and protease specificity was set to Trypsin. Protein quantitation was undertaken using MaxLFQ ^26^ based analysis. The precursor PEP was altered to 0.01 from the default 0.2 to improve quantitative accuracy, with all single peptide protein matches excluded. Statistical analysis was undertaken using Perseus ^27^ with missing values imputed based on the total observed protein intensities with a range of 0.3 σ and a downshift of 2.5 σ. Biological replicates were grouped together, and student t-tests were used to compare individual groups with a minimum fold change of +/- 1 considered for further analysis. Multiple hypothesis correction was undertaken using a permutation-based FDR approach allowing an FDR of 5%. Enrichment analysis using Fisher exact tests was undertaken in Perseus using Gene Ontology (GO) terms obtained from Uniprot (*B. cenocepacia* strain J2315 proteome: UP000001035) as well as against proteins previously reported as altered within K56-2 Δ*pglL,* K56-2 Δ*ogc or* K56-2 Δ*pglL*Δ*ogc* compared to K56-2 WT by Oppy *et al.* ^3^. Data visualization was undertaken using ggplot2 ^23^ in R.


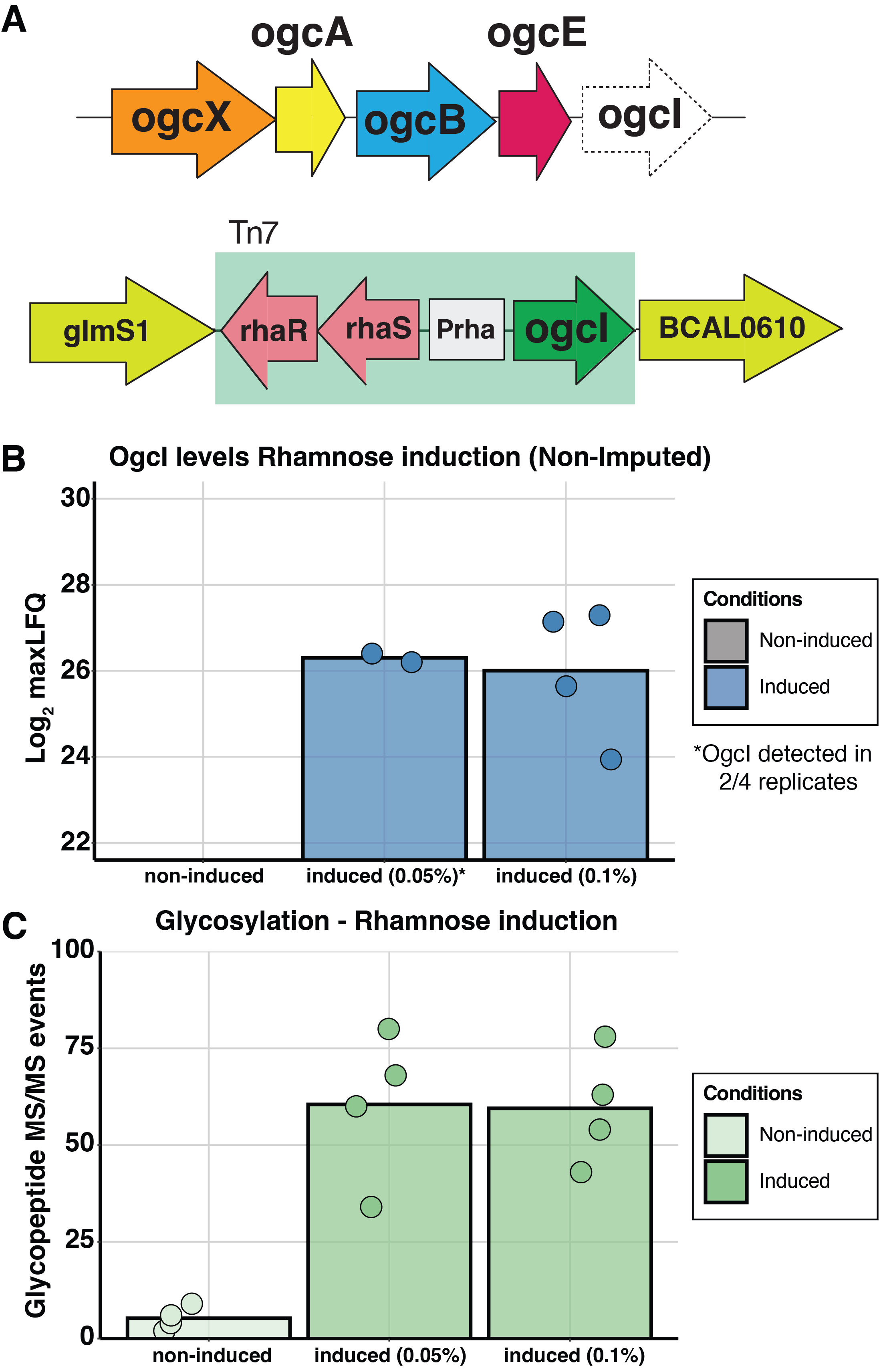


**Supplementary Figure 1. Proteomic/glycoproteomic validation of *B. cenocepacia* Δ*ogcI* Tn7-*ogcI*. A)** Graphic representation of the *ogc* cluster (BCAL3114–BCAL3118) demonstrating the loss of *ogcI* (BCAL3118) and integration of rhamnose-inducible *ogcI* downstream of *glmS1*. Before generating *ogc* mutants, inducible expression and restoration of glycosylation were confirmed by proteomic analysis. Induction of *ogcI* expression by 0.05% and 0.1% rhamnose led to the restoration of both **B)** OgcI and **C)** Glycosylation, as determined by identified glycopeptides, in the strain *B. cenocepacia* Δ*ogcI* Tn7-*ogcI*.

**
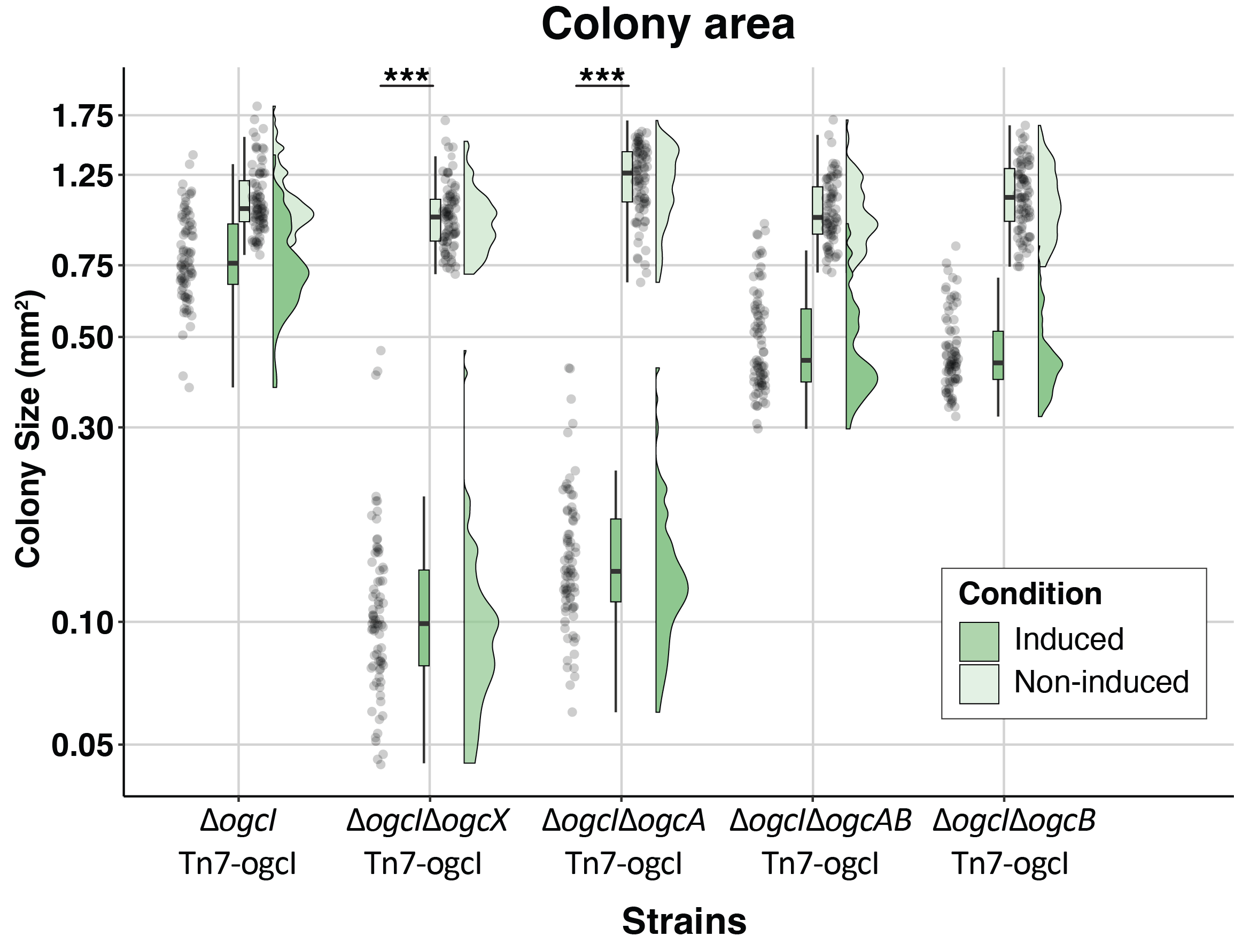
**

**Supplementary Figure 2. Quantitative assessment of Δ*ogcX,* Δ*ogcA,* Δ*ogcB and* Δ*ogcAB* colony sizes with and without 1% rhamnose induction.** Analysis of at least 20 colonies per independent replicate reveals Δ*ogcX* and Δ*ogcA* strains possess a reduced colony size in response to the initiation of glycosylation leading to colonies ~10% the size of *B. cenocepacia* Δ*ogcI* Tn7-*ogcI* and strains without induction (n=4).

**
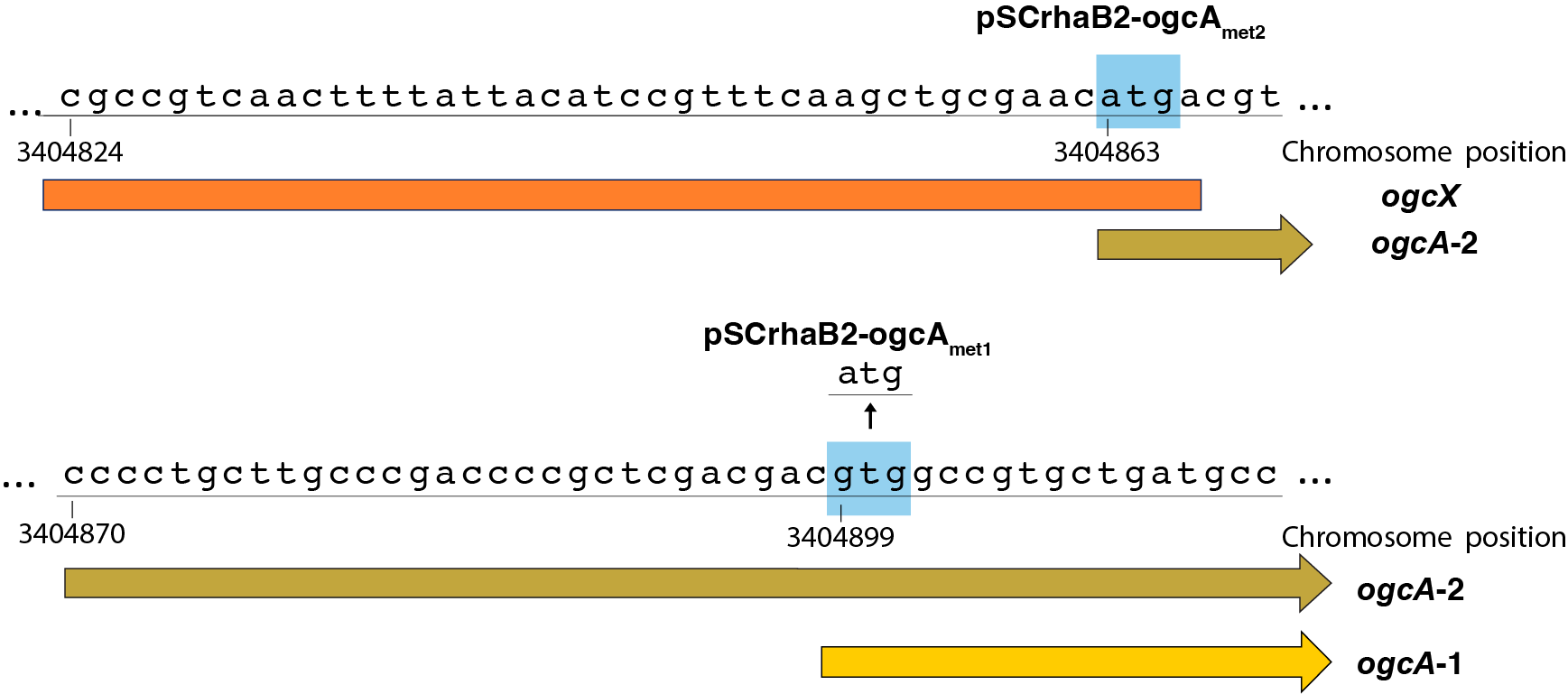
**

**Supplementary Figure 3. Start codon assignments of OgcA used for OgcA complementation within *B. cenocepacia* Δ*ogcI* Δ*ogcA* Tn7-*ogcI.*** Graphic representation of putative start sites of *ogcA*. Due to the lack of *ogcA* expression from the annotated start codon, the gene was cloned into the pSCrhaB2 plasmid from two alternative start codons: a GTG (valine) converted to ATG, and a start codon located further upstream were designated as *ogcA*_Met1_ and *ogcA*_Met2_, respectively.

**
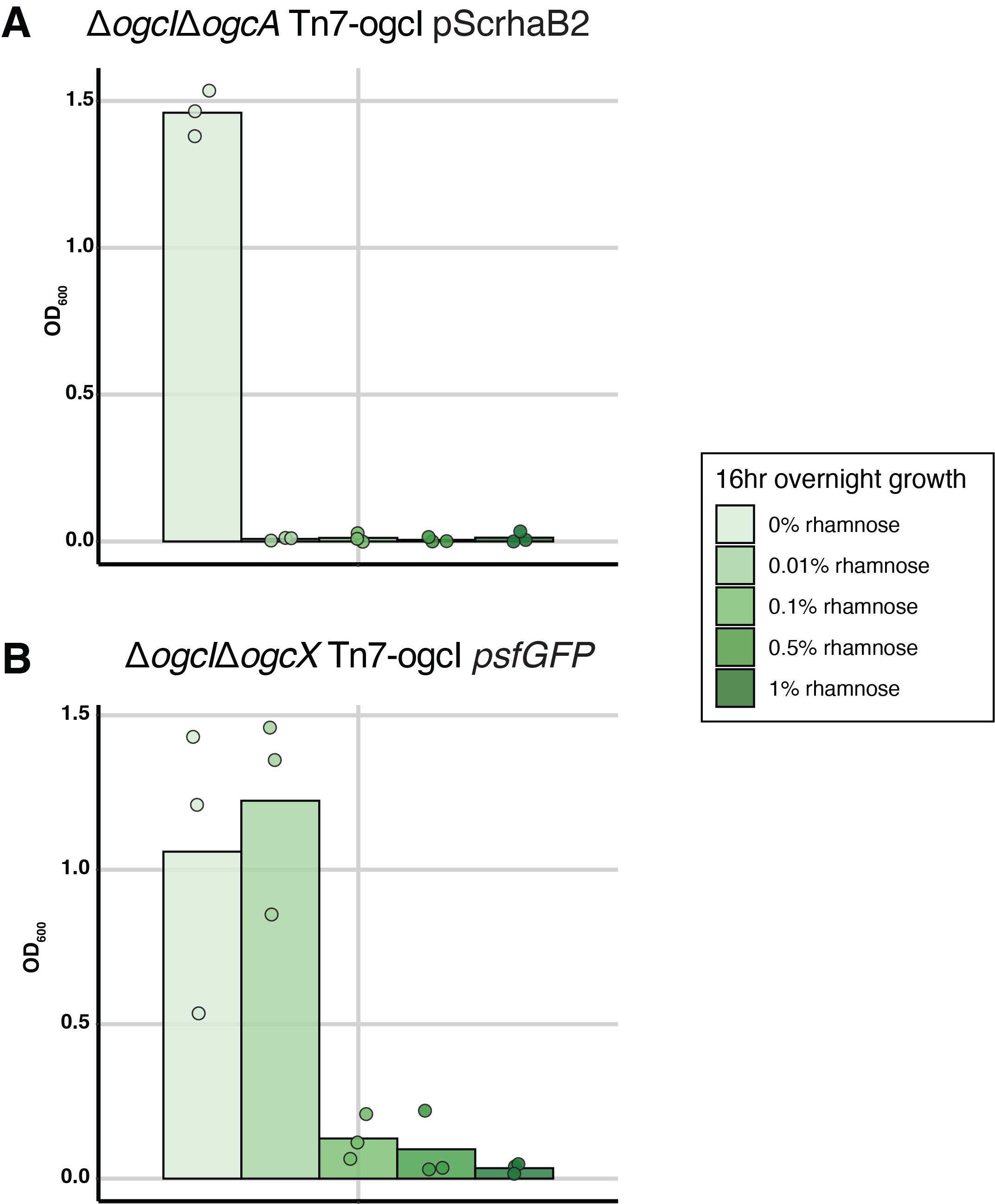
**

**Supplementary Figure 4. Viability of *B. cenocepacia* Δ*ogcI*Δ*ogcA* Tn7-*ogcI* and Δ*ogcI*Δ*ogcX* Tn7-ogcI containing control plasmids in response to induction. A)** Overnight growth measurements of Δ*ogcI*Δ*ogcA* Tn7-*ogcI* carrying pSCrhaB2 induced under different concentrations of rhamnose reveal that the induction of OgcI under growth conditions to maintain plasmids results in a loss of viability. **B)** Overnight growth measurements of Δ*ogcI*Δ*ogcX* Tn7-*ogcI* carrying *psfGFP* induced under different concentrations of rhamnose reveal that the induction of OgcI under growth conditions to maintain plasmids results in a loss of viability at rhamnose concentrations above 0.01%.

**Supplementary Figure 5. Proteomic analysis of the proteins of the *ogc* within Δ*ogcI*Δ*ogcX* Tn7-*ogcI*, Δ*ogcI*Δ*ogcA* Tn7-*ogcI*, Δ*ogcI*Δ*ogcB* Tn7-*ogcI* and Δ*ogcI* Tn7-*ogcI* strains.** DIA proteomic analysis demonstrates the selective absence of the OGC proteins within mutants and confirms that these mutations are non-polar, with the abundance of undisrupted OGC proteins equivalent to that in the parental strain (Δ*ogcI* Tn7-*ogcI*).


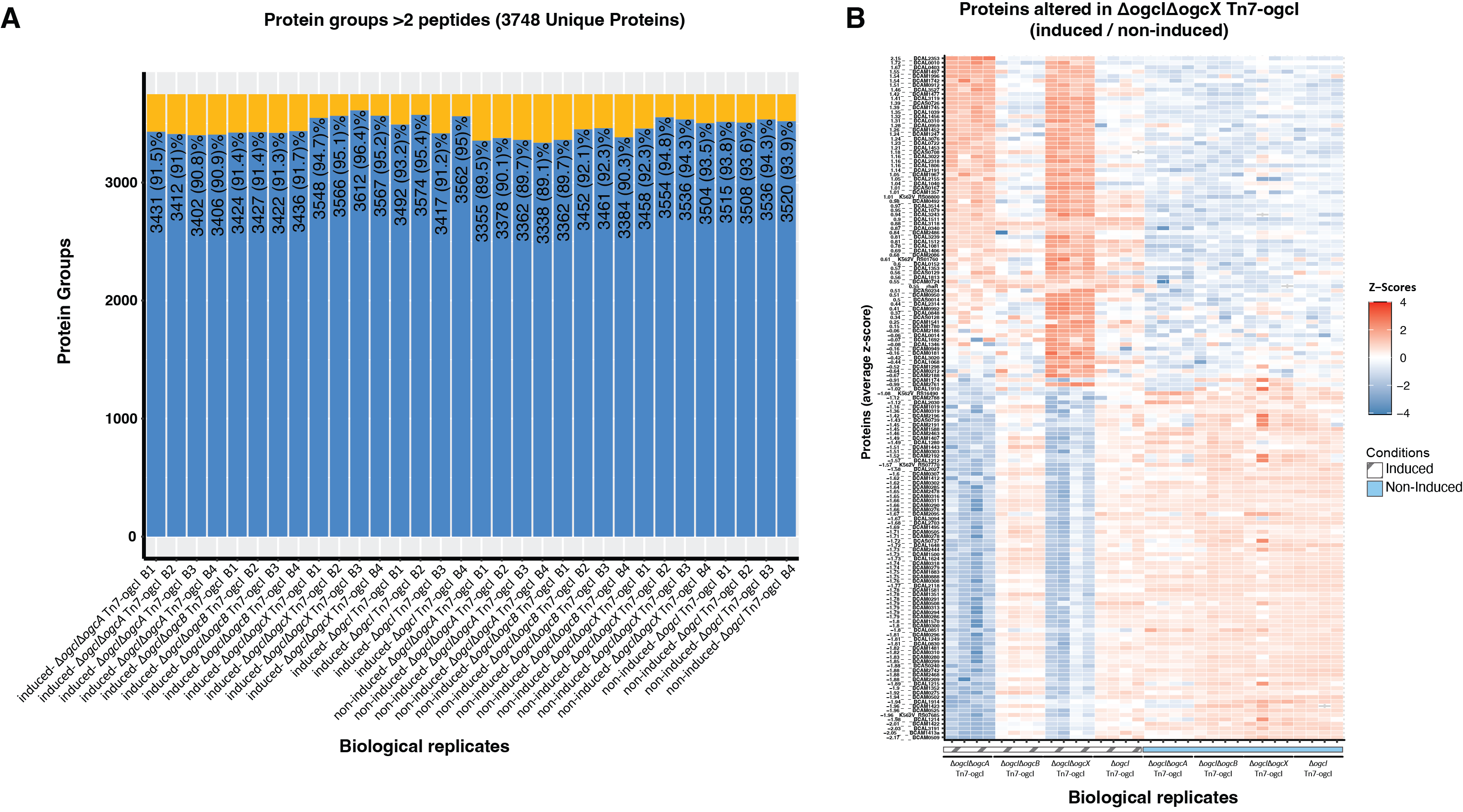


**Supplementary Figure 6. Proteomic analysis of *B. cenocepacia* Δ*ogcI*** **Tn7-*ogcI* strains. A)** Proteomic coverage of *B. cenocepacia* proteins observed across biological replicates demonstrate that >90% of all proteins were identified within samples with at least two unique precursors**. B)** Heatmap of proteomic changes associated with Z-score > ±2 in at least one biological group reveals similar alterations observed within Δ*ogcI*Δ*ogcX* Tn7-*ogcI* and Δ*ogcI*Δ*ogcA* Tn7-*ogcI* upon induction of OgcI.

**
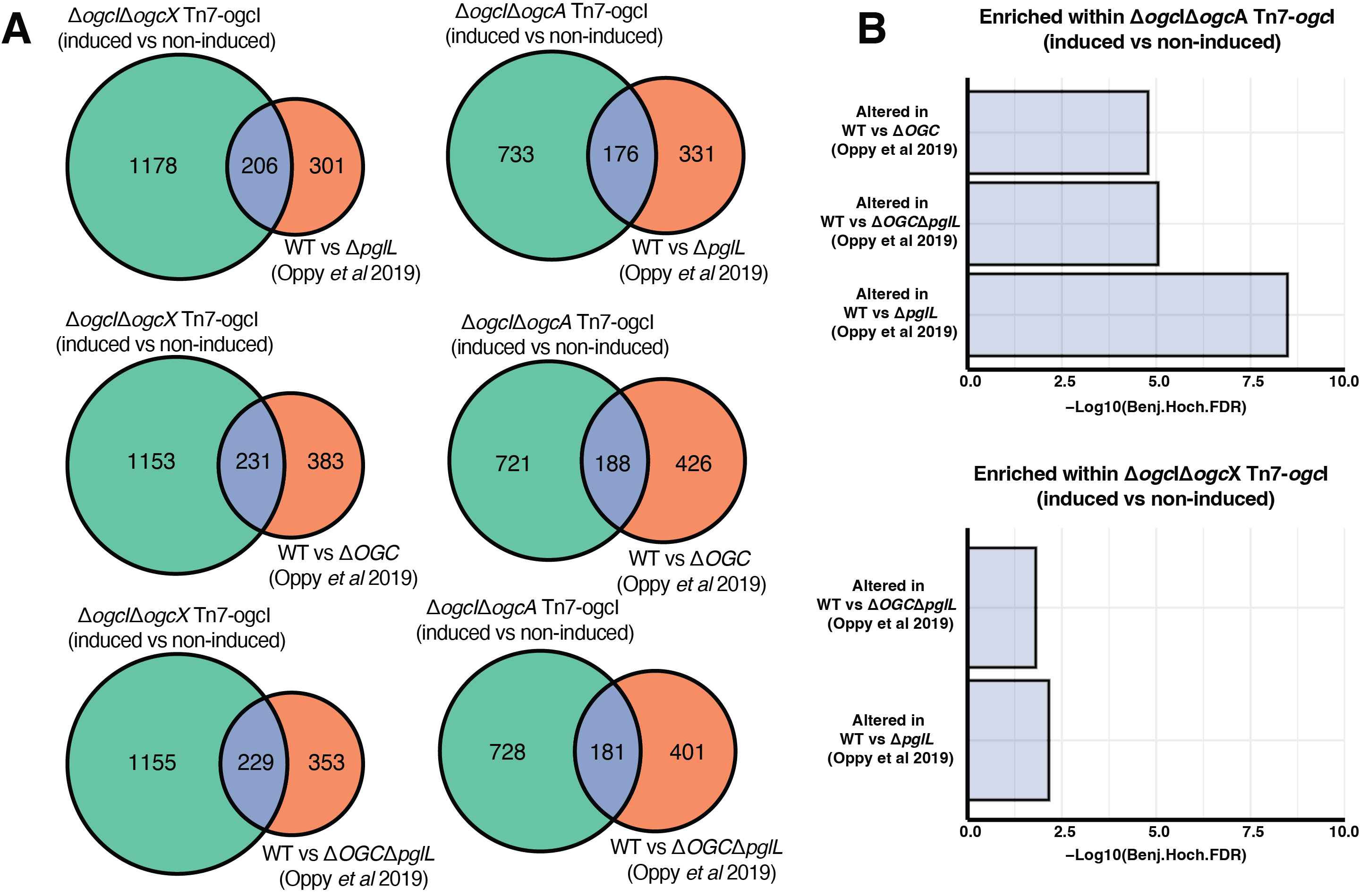
**

**Supplementary Figure 7. Comparison of proteomic alterations observed within *B. cenocepacia* Δ*ogc*IΔ*ogc*X Tn7-*ogc*I and Δ*ogc*IΔ*ogc*A Tn7-ogcI compared to *B. cenocepacia* glycosylation-null strains. A)** Venn diagrams showing the overlap in proteomic changes observed within Δ*ogc*IΔ*ogc*X Tn7-*ogc*I and Δ*ogc*IΔ*ogc*A Tn7-ogcI upon induction, compared to previously reported proteomic alterations in *B. cenocepacia* glycosylation-null strains Δ*pgl*L, Δ*ogc*, and Δ*pgl*LΔ*OGC*, relative to *B. cenocepacia* K56-2 WT ^3^. **B)** Enrichment analysis of the overlap in altered proteins within Δ*ogc*IΔ*ogc*X Tn7-ogcI and Δ*ogc*IΔ*ogc*A Tn7-ogcI upon induction, compared to previously reported proteomic alterations in the *B. cenocepacia* glycosylation-null strains Δ*pgl*L, Δ*OGC*, and Δ*pgl*LΔ*OGC*, relative to *B. cenocepacia* K56-2 WT, reveals a statistically significant enrichment. These findings support similar proteomic alterations between the induced strains and the glycosylation-null strains.

**
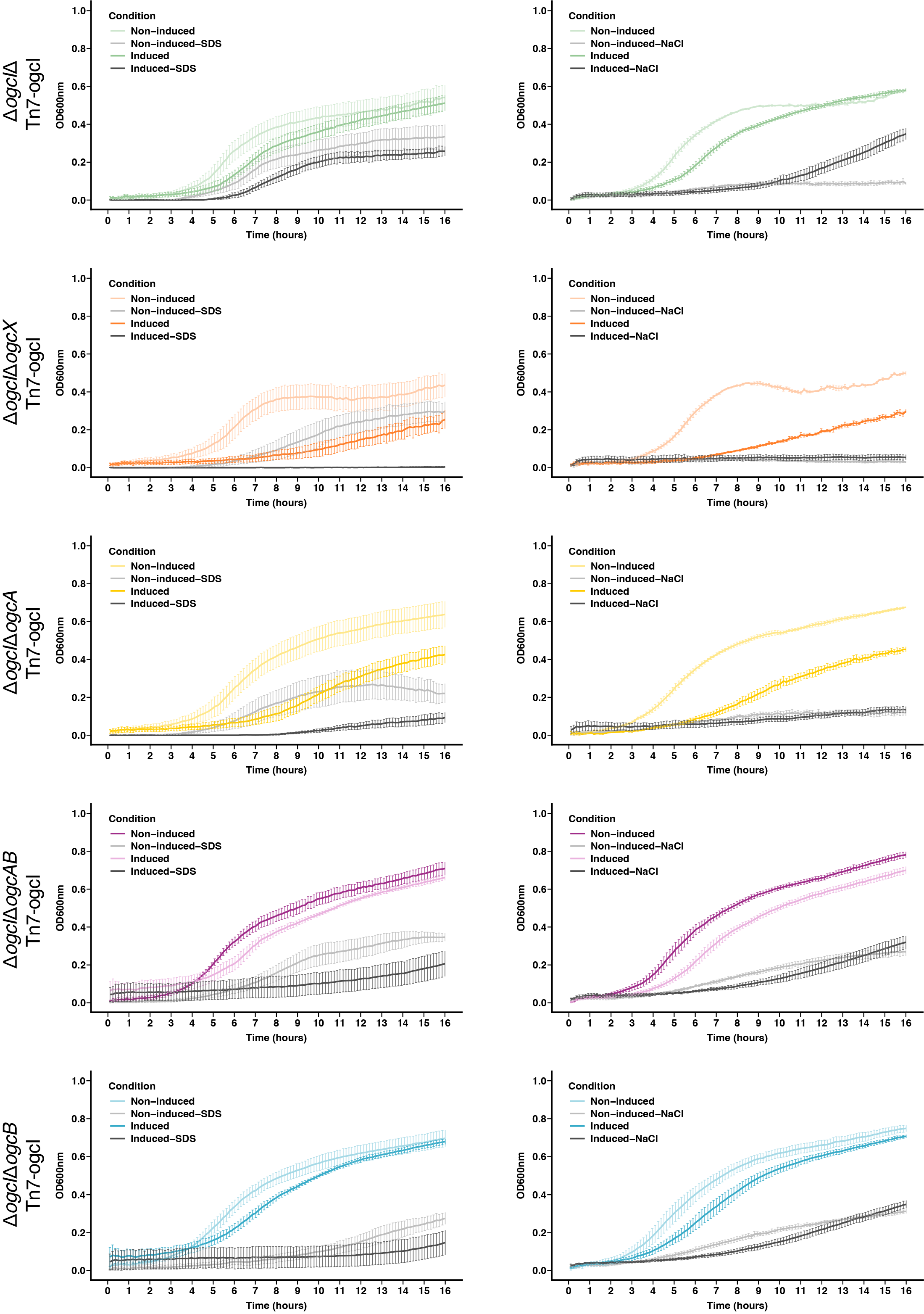
**

**Supplementary Figure 8. Plate-based growth assays of Δ*ogcI* Tn7-*ogcI,* Δ*ogcI*Δ*ogcX* Tn7-*ogcI*, Δ*ogcI*Δ*ogcA* Tn7-*ogcI*, Δ*ogcI*Δ*ogcAB* Tn7-*ogcI* and Δ*ogcI*Δ*ogcB* Tn7-*ogcI* strains in the presence of membrane / osmotic stress agents.** Growth curves of the strains subjected to membrane stress (0.01% SDS) and osmotic stress (2% NaCl) with and without 1% rhamnose induction. The growth curves demonstrate that the deletion of *ogcX* and *ogcA* results in nearly total loss of growth in the presence of 0.01% SDS when glycosylation is initiated. In the presence of osmotic stress (2% NaCl), both mutants lacking *ogcX* and *ogcA* failed to grow regardless of induction.

**
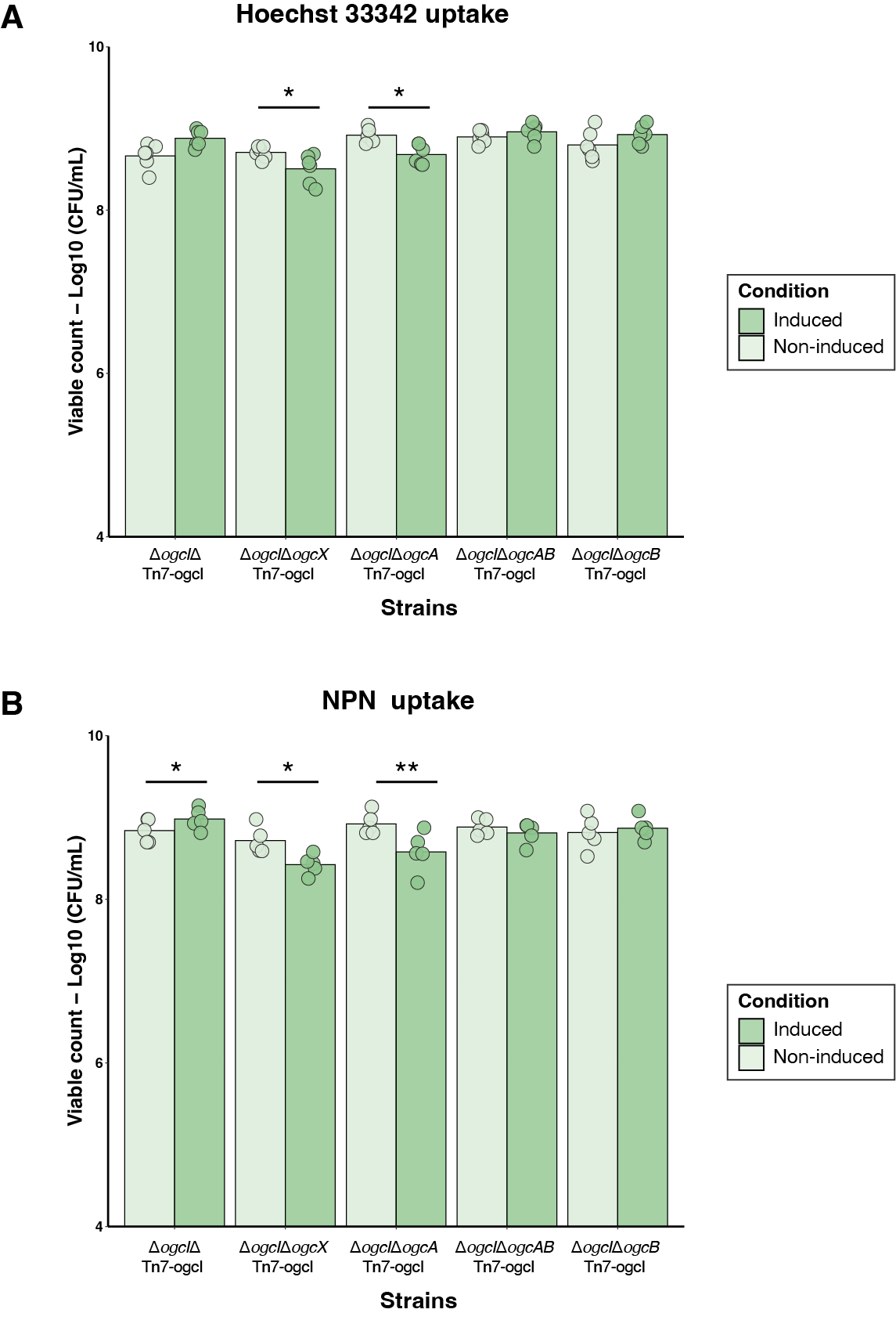
**

**Supplementary Figure 9. Viable counts of Hoechst and NPN uptake assays.** Viable counts of the parental strain and mutants demonstrate viability of strains prior to the addition of Hoechst 33342 and NPN dyes. Bacterial suspensions were serially diluted and spotted onto LB agar plates, and colonies counted after 48 hours of incubation at 37°C. Due to the presence of sodium azide in the buffer used for washing cells in the NPN assay, bacterial suspensions from the same overnight culture and OD, but resuspended in PBS, were used for determining the viable cell counts.

**
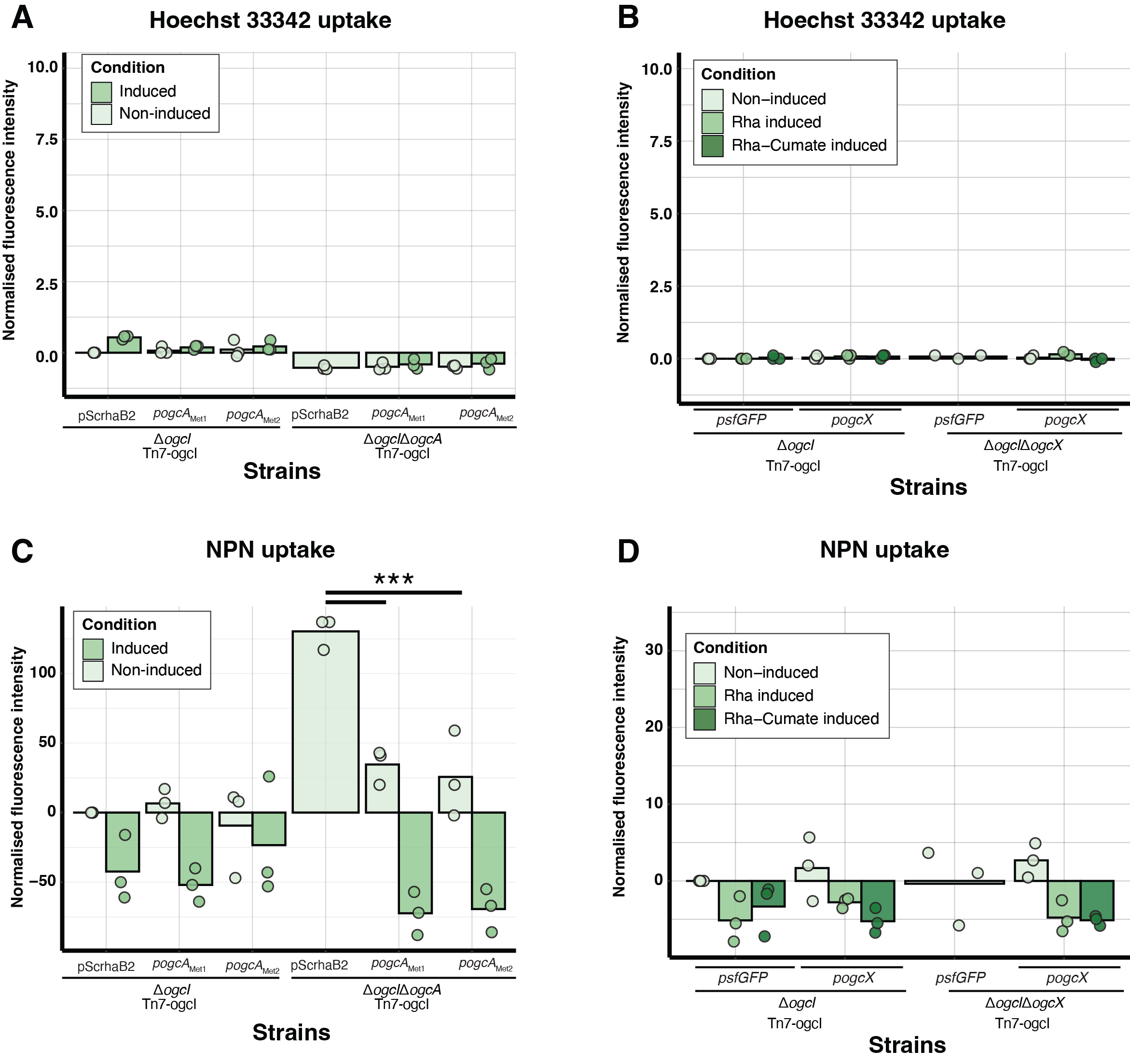
**

**Supplementary Figure 10. Hoechst and NPN uptake assays of complemented Δ*ogc*IΔ*ogc*A Tn7-*ogc*I and Δ*ogc*IΔ*ogc*X Tn7-*ogc*I. Hoechst 33342 (A/C) and NPN (B/D) uptake assays reveal that complementation of Δ*ogc*IΔ*ogc*A Tn7-*ogc*I and Δ*ogc*IΔ*ogc*X Tn7-*ogc*I reduces dye uptake to levels equivalent to Δ*ogc*I Tn7-*ogc*I containing plasmids (n=3 for Hoechst 33342, n=3 for NPN). The fluorescence intensities have been normalized against bacterial cell counts.**

**
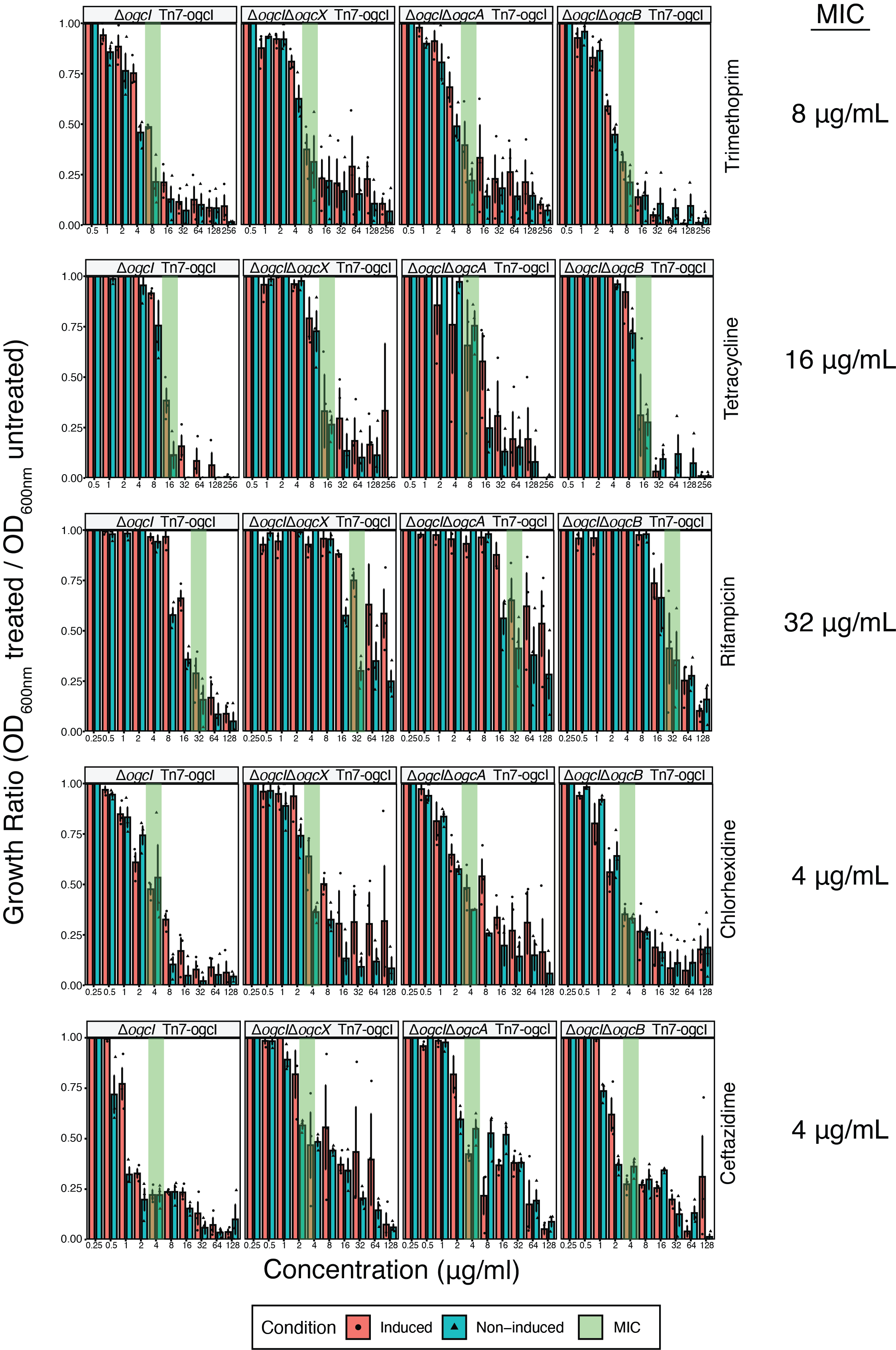
**

**Supplementary Figure 11. Antibiotic susceptibility assays of glycosylation inducible *B. cenocepacia* strains, with and without 1% rhamnose induction.** The growth of antibiotic-treated bacteria was measured by reading absorbance at 600 nm (OD_600nm_) and normalised to the OD_600nm_ of the lowest antibiotic concentration. Individual values (dots) represent normalised values for three biological replicates, and bars represent the mean of the three replicates.

**
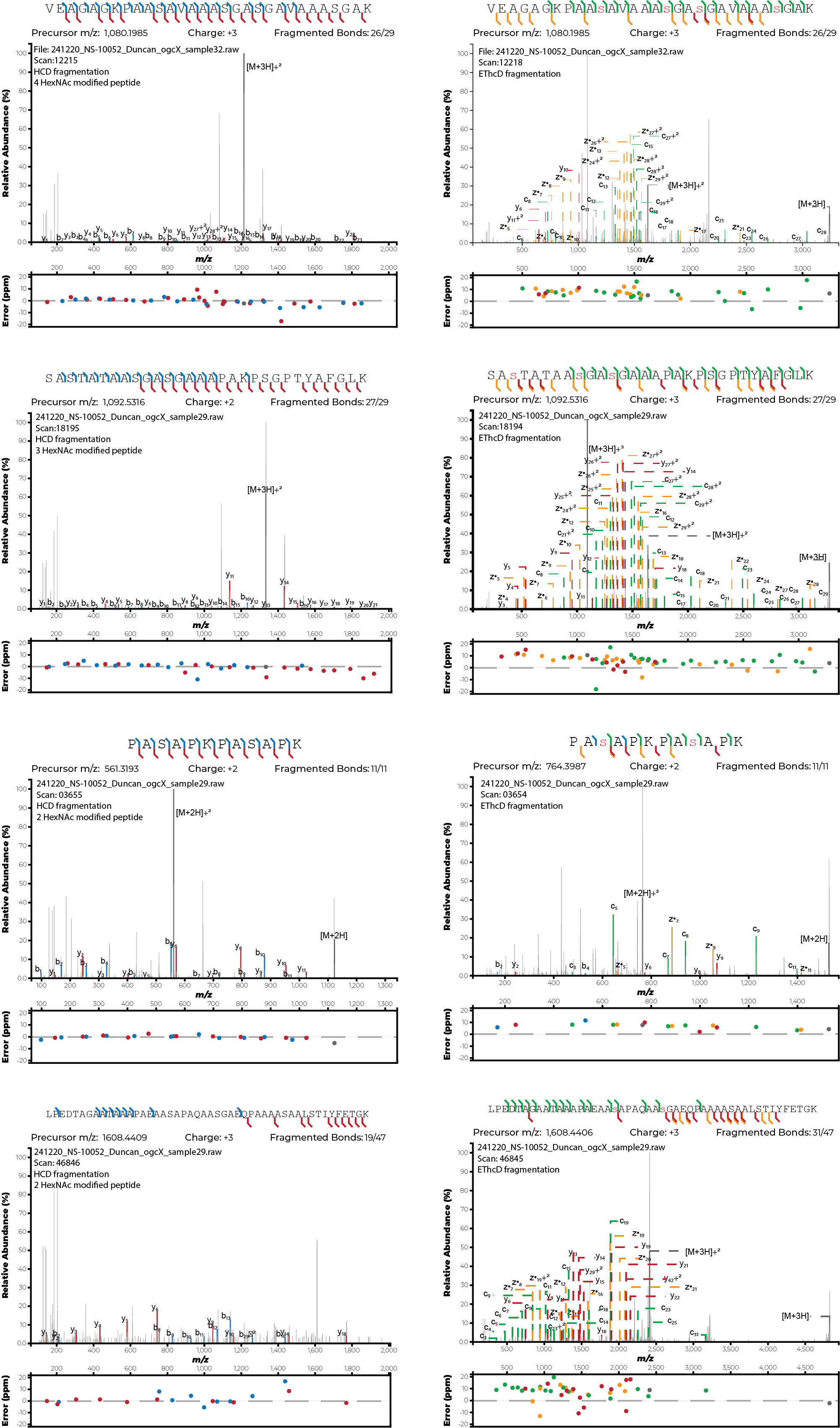
**

**Supplementary Figure 12. *B. cenocepacia* Δ*ogcI*Δ*ogcB* Tn7-*ogcI* Glycopeptides:** Manual inspection of EThcD and HCD glycopeptide spectra observed within *B. cenocepacia* Δ*ogcI*Δ*ogcB* Tn7-*ogcI* containing p*ogcX* demonstrates that assigned HexNAc2-modified glycopeptides correspond to peptides with multiple single HexNAc residues attached to distinct serine residues**.**

**
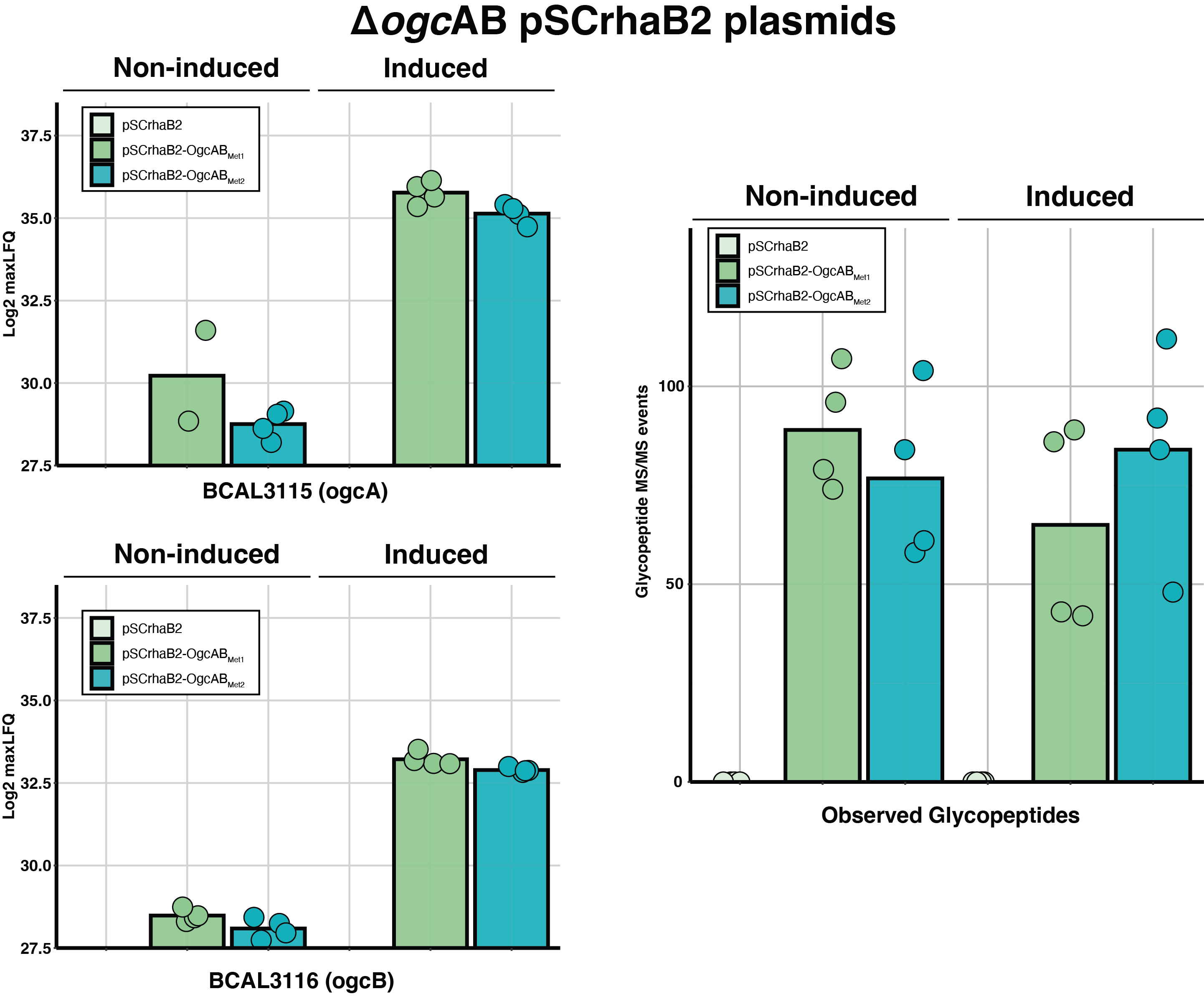
**

**Supplementary Figure 13. Proteomic analysis of pSCrhaB2-*ogcAB in* Δ*ogcAB*.** Quantification of OgcA and OgcB from whole-cell proteomic analysis of *B. cenocepacia* Δ*ogcAB* strains carrying empty pSCrhaB2 or pSCrhaB2-*ogcAB* constructs from two different start sites confirm restoration of OgcA and OgcB as well as glycosylation. Production of OgcA and OgcB was observed in the non-induced conditions with comparable levels of glycosylation observed to samples induced with 0.05% Rhamnose. Proteomic and glycopeptide data is provided in Supplementary Table 14 and 15.


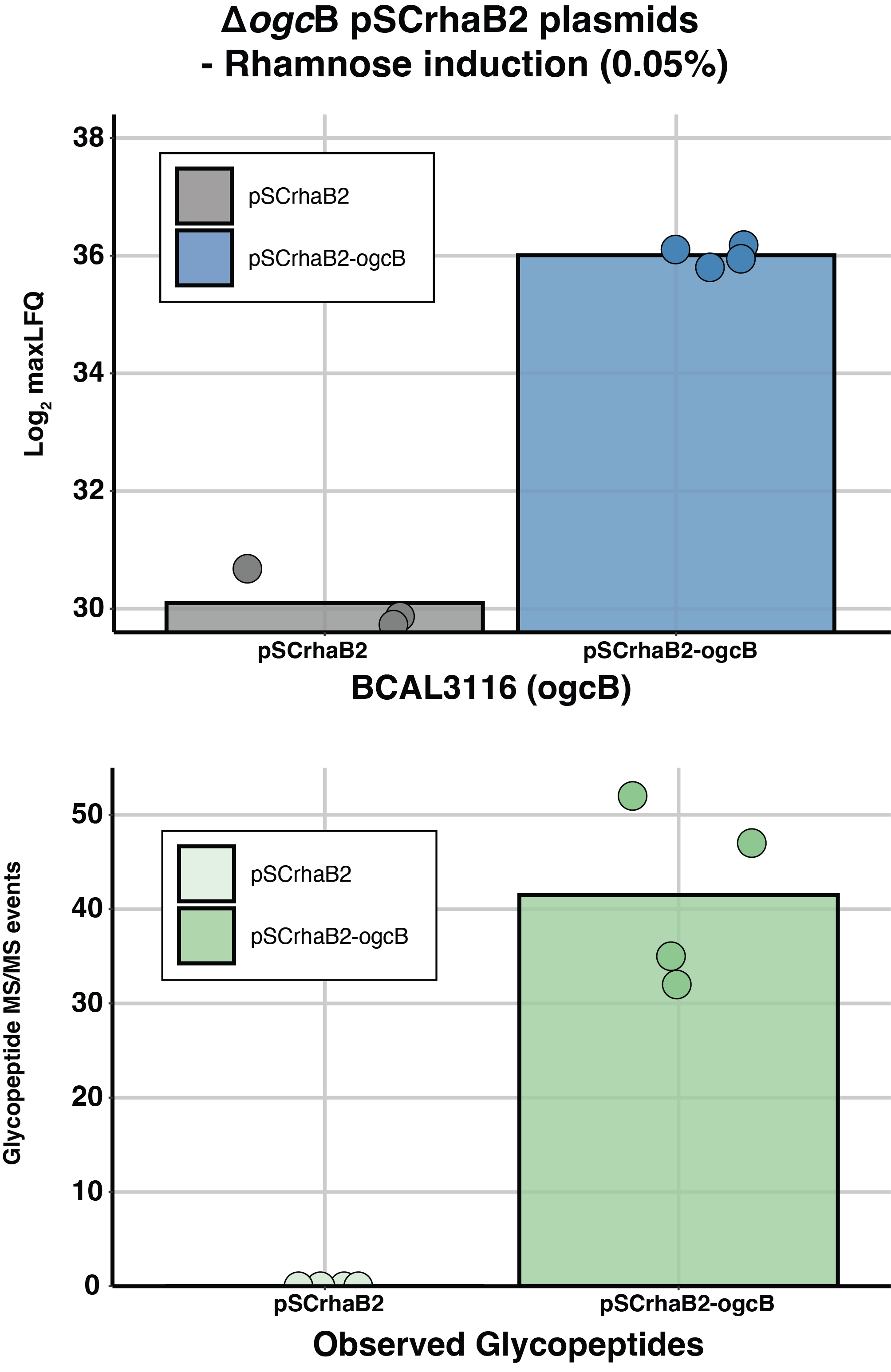


**Supplementary Figure 14. Proteomic analysis of pSCrhaB2-*ogcB* in Δ*ogcB*.** Quantification of OgcB from whole-cell proteomic analysis of *B. cenocepacia* Δ*ogcB* strains carrying empty pSCrhaB2 or pSCrhaB2-*ogcB* grown with 0.05% rhamnose induction. Induction resulted in elevated levels of OgcB within *B. cenocepacia* Δ*ogcB* carrying pSCrhaB2-*ogcB* and the restoration of glycosylation. Proteomic and glycopeptide data is provided in Supplementary Table 16 and 17.


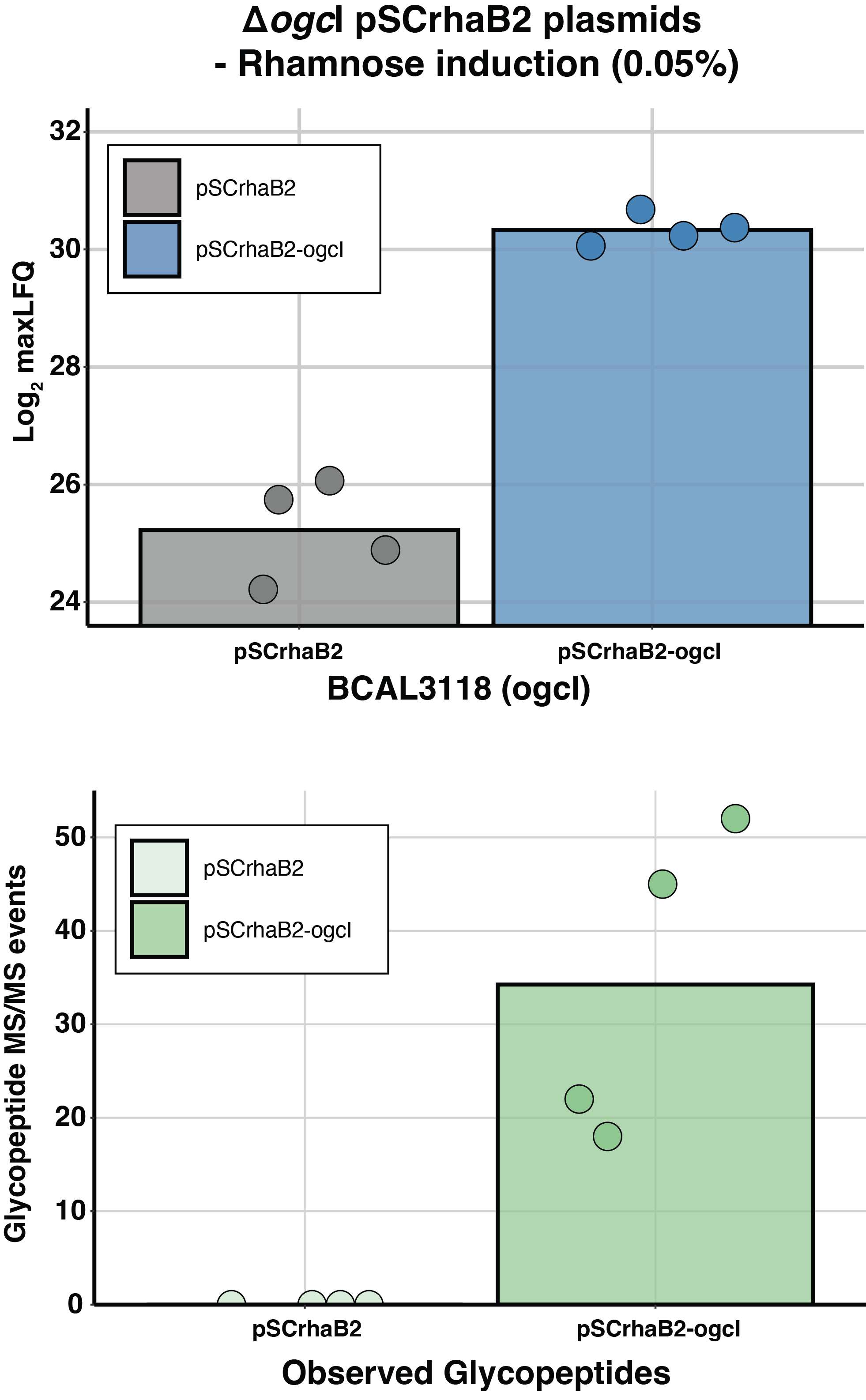


**Supplementary Figure 15. Proteomic analysis of pSCrhaB2-*ogcI* in Δ*ogcI*.** Quantification of OgcI from whole-cell proteomic analysis of *B. cenocepacia* Δ*ogcI* strains carrying empty pSCrhaB2 or pSCrhaB2-*ogcI* grown with 0.05% rhamnose induction. Induction resulted in elevated levels of OgcI within *B. cenocepacia* Δ*ogcI* carrying pSCrhaB2-*ogcI* and the restoration of glycosylation. Proteomic and glycopeptide data is provided in Supplementary Table 18 and 19.


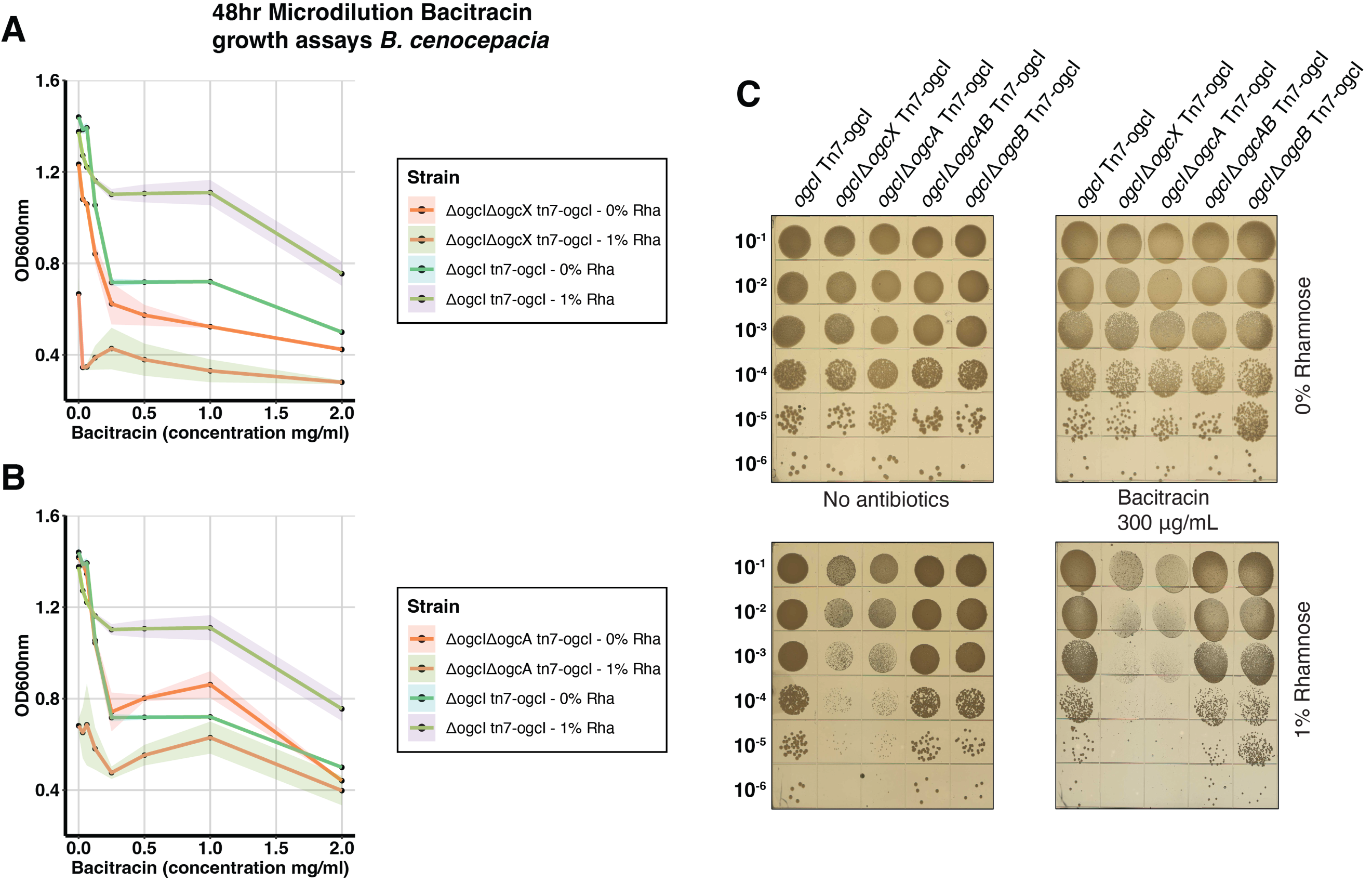


**Supplementary Figure 16. Loss of *ogcA* and *ogcX* sensitises *B. cenocepacia* to Bacitracin.** Microplate dilution assays of strains grown for 48 hours at 37°C with Bacitracin 2 mg/ml, 1 mg/ml, 0.5 mg/ml, 0.25 mg/ml, 0.125 mg/ml, 0.0625 mg/ml, and 0.03125 mg/ml with and without 1% Rhamnose supports reduced growth in the presence of **Bacitracin (n=3) in A)** Δ*ogcI* Tn7-*ogcI* and Δ*ogcI*Δ*ogcA* Tn7-*ogcI* and **B)** Δ*ogcI* Tn7-*ogcI* and Δ*ogcI*Δ*ogcX* Tn7-*ogcI* **C)** Spot plate assays of *B. cenocepacia* strains, including Δ*ogcI* Tn7-*ogcI*, Δ*ogcI*Δ*ogcX* Tn7-*ogcI*, Δ*ogcI*Δ*ogcA* Tn7-*ogcI*, Δ*ogcI*Δ*ogcAB* Tn7-*ogcI* and Δ*ogcI*Δ*ogcB* Tn7-*ogcI* on LB containing **Bacitracin**, with or without induction with 1% rhamnose. Rhamnose-induced glycosylation resulted in increased susceptibility to **Bacitracin**. Previous studies have demonstrated the MIC of **Bacitracin for** *B. cenocepacia* K56-2 is 32,000 μg/ml ^28^ with 300 μg/ml representing 1/100 of the MIC. Data representative of three biological replicates.

**
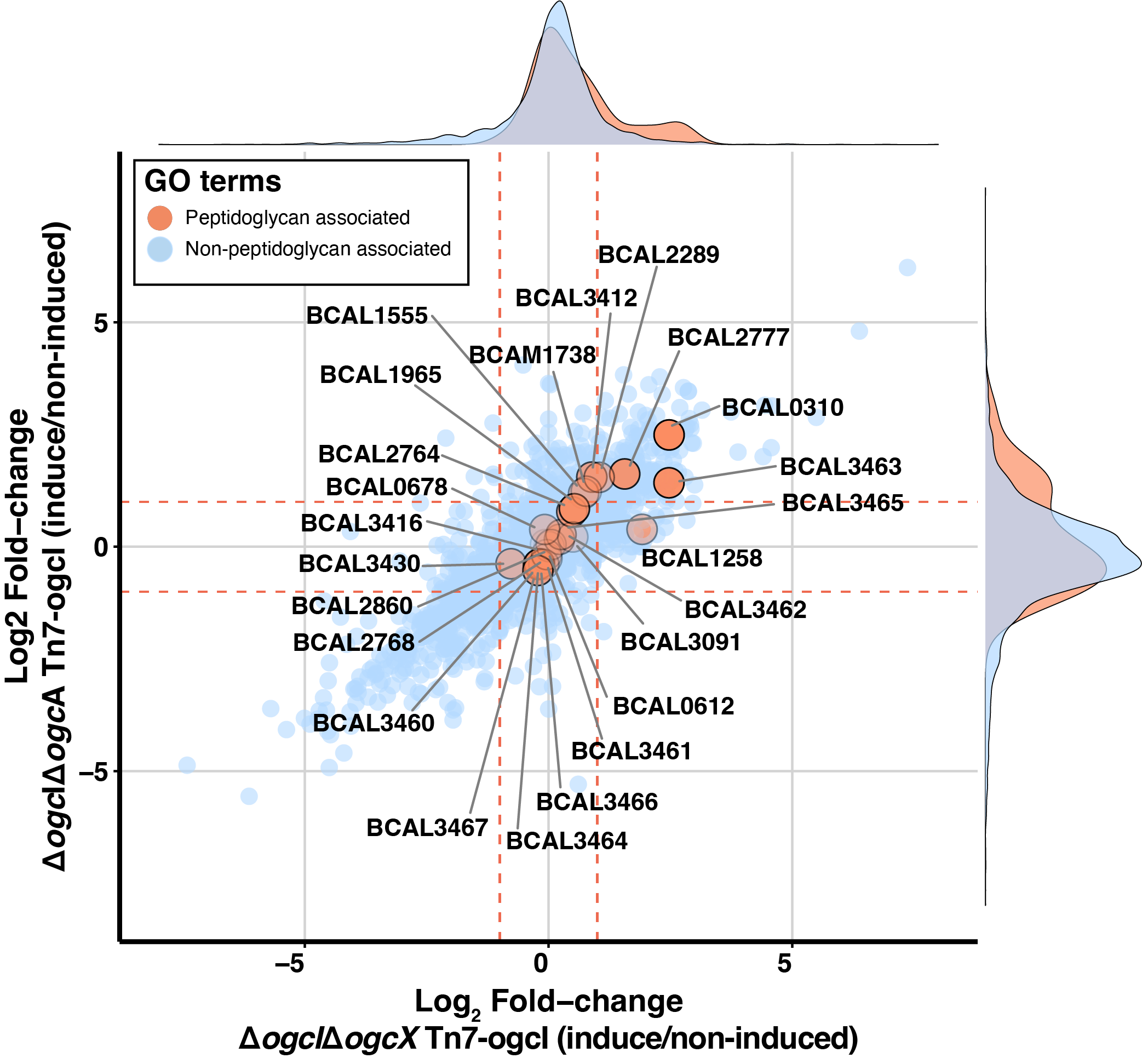
**

**Supplementary Figure 17. Proteomic analysis of peptidoglycan associated proteins within Δ*ogcI*Δ*ogcA* Tn7-*ogcI* and Δ*ogcI*Δ*ogcX* Tn7-*ogcI* in response to glycosylation initiation.** DIA proteomic analysis of Δ*ogcI*Δ*ogcA* Tn7-*ogcI* and Δ*ogcI*Δ*ogcX* Tn7-*ogcI* strains with and without rhamnose induction, with proteins assigned to the GO terms GO:0042834, GO:0009253, GO:0009252, or GO:0000270 as denoted in Supplementary Table 13 highlighted in orange.


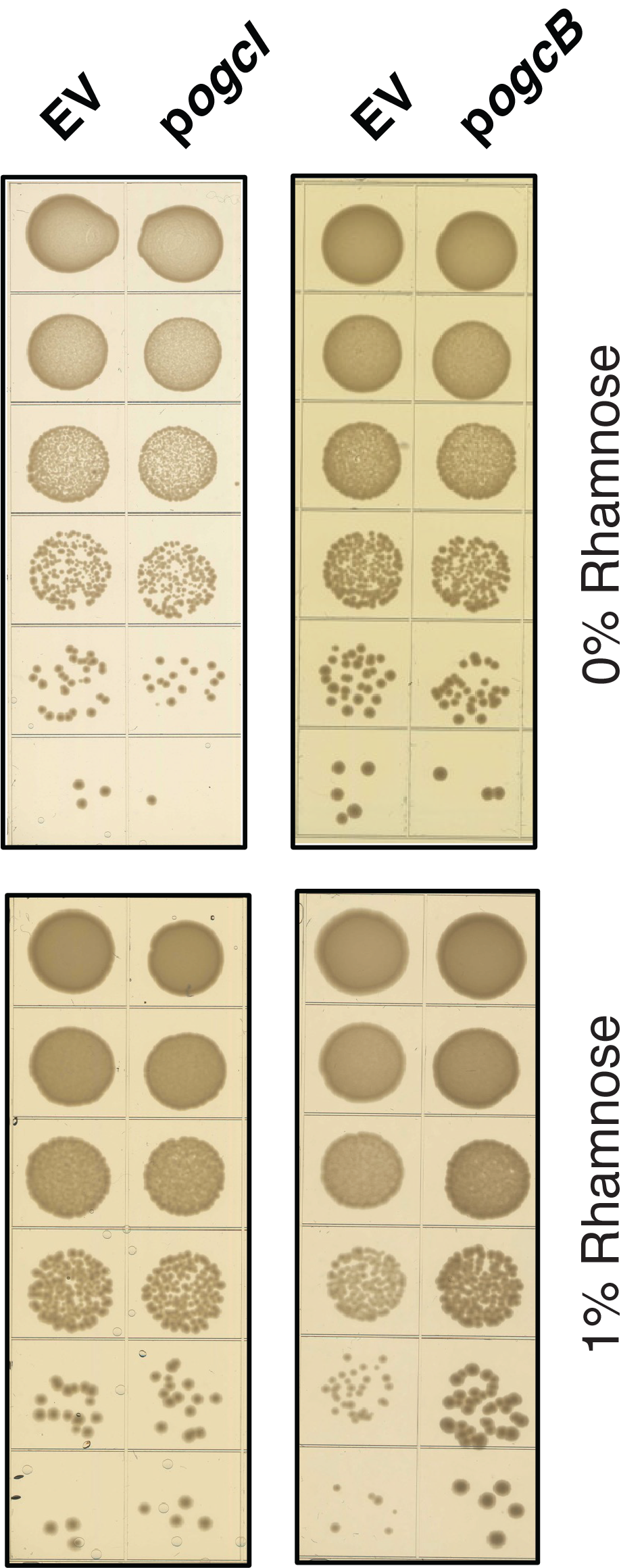


**Supplementary Figure 18. Spot plate assays of *E. coli* containing the expression vectors pSCrhaB2 (EV), pSCrhaB2-*ogcI (*p*ogcI)* and pSCrhaB2-*ogcB* (p*ogcB*).** Induction of *ogc*I and *ogc*B with 1% rhamnose does not impact the growth of *E. coli.*

**References**

1 Lopez, C. M., Rholl, D. A., Trunck, L. A. & Schweizer, H. P. Versatile dual-technology system for markerless allele replacement in Burkholderia pseudomallei. *Applied and environmental microbiology* **75**, 6496-6503 (2009). <https://doi.org:10.1128/AEM.01669-09>

2 Darling, P., Chan, M., Cox, A. D. & Sokol, P. A. Siderophore production by cystic fibrosis isolates of Burkholderia cepacia. *Infection and immunity* **66**, 874-877 (1998).

3 Oppy, C. C. *et al.* Loss of O-linked protein glycosylation in Burkholderia cenocepacia impairs biofilm formation, siderophore activity and alters transcriptional regulators *mSphere* **4**, e00660-00619 (2019).

4 Figurski, D. H. & Helinski, D. R. Replication of an origin-containing derivative of plasmid RK2 dependent on a plasmid function provided in trans. *Proceedings of the National Academy of Sciences of the United States of America* **76**, 1648-1652 (1979). <https://doi.org:10.1073/pnas.76.4.1648>

5 Garcia, E. C., Anderson, M. S., Hagar, J. A. & Cotter, P. A. Burkholderia BcpA mediates biofilm formation independently of interbacterial contact-dependent growth inhibition. *Molecular microbiology* **89**, 1213-1225 (2013). <https://doi.org:10.1111/mmi.12339>

6 Hamad, M. A., Skeldon, A. M. & Valvano, M. A. Construction of aminoglycoside-sensitive Burkholderia cenocepacia strains for use in studies of intracellular bacteria with the gentamicin protection assay. *Applied and environmental microbiology* **76**, 3170-3176 (2010). <https://doi.org:10.1128/AEM.03024-09>

7 Choi, K. H., DeShazer, D. & Schweizer, H. P. mini-Tn7 insertion in bacteria with multiple glmS-linked attTn7 sites: example Burkholderia mallei ATCC 23344. *Nature protocols* **1**, 162-169 (2006). <https://doi.org:10.1038/nprot.2006.25>

8 Cardona, S. T. & Valvano, M. A. An expression vector containing a rhamnose-inducible promoter provides tightly regulated gene expression in Burkholderia cenocepacia. *Plasmid* **54**, 219-228 (2005). <https://doi.org:10.1016/j.plasmid.2005.03.004>

9 Flannagan, R. S., Linn, T. & Valvano, M. A. A system for the construction of targeted unmarked gene deletions in the genus Burkholderia. *Environ Microbiol* **10**, 1652-1660 (2008). <https://doi.org:10.1111/j.1462-2920.2008.01576.x>

10 Lefebre, M. D. & Valvano, M. A. Construction and evaluation of plasmid vectors optimized for constitutive and regulated gene expression in Burkholderia cepacia complex isolates. *Applied and environmental microbiology* **68**, 5956-5964 (2002).

11 Aubert, D. F., Hamad, M. A. & Valvano, M. A. A markerless deletion method for genetic manipulation of Burkholderia cenocepacia and other multidrug-resistant gram-negative bacteria. *Methods in molecular biology* **1197**, 311-327 (2014). <https://doi.org:10.1007/978-1-4939-1261-2_18>

12 Kulak, N. A., Pichler, G., Paron, I., Nagaraj, N. & Mann, M. Minimal, encapsulated proteomic-sample processing applied to copy-number estimation in eukaryotic cells. *Nature methods* **11**, 319-324 (2014). <https://doi.org:10.1038/nmeth.2834>

13 Harney, D. J. *et al.* Proteomic Analysis of Human Plasma during Intermittent Fasting. *Journal of proteome research* **18**, 2228-2240 (2019). <https://doi.org:10.1021/acs.jproteome.9b00090>

14 Rappsilber, J., Mann, M. & Ishihama, Y. Protocol for micro-purification, enrichment, pre-fractionation and storage of peptides for proteomics using StageTips. *Nature protocols* **2**, 1896-1906 (2007). <https://doi.org:10.1038/nprot.2007.261>

15 Ahmad Izaham, A. R. *et al.* What Are We Missing by Using Hydrophilic Enrichment? Improving Bacterial Glycoproteome Coverage Using Total Proteome and FAIMS Analyses. *Journal of proteome research* **20**, 599-612 (2021). <https://doi.org:10.1021/acs.jproteome.0c00565>

16 Saba, J., Dutta, S., Hemenway, E. & Viner, R. Increasing the productivity of glycopeptides analysis by using higher-energy collision dissociation-accurate mass-product-dependent electron transfer dissociation. *Int J Proteomics* **2012**, 560391 (2012). <https://doi.org:10.1155/2012/560391>

17 Caval, T., Zhu, J. & Heck, A. J. R. Simply Extending the Mass Range in Electron Transfer Higher Energy Collisional Dissociation Increases Confidence in N-Glycopeptide Identification. *Anal Chem* **91**, 10401-10406 (2019). <https://doi.org:10.1021/acs.analchem.9b02125>

18 Polasky, D. A., Geiszler, D. J., Yu, F. & Nesvizhskii, A. I. Multi-attribute Glycan Identification and FDR Control for Glycoproteomics. *Molecular & cellular proteomics : MCP*, 100205 (2022). <https://doi.org:https://doi.org/10.1016/j.mcpro.2022.100205>

19 Kong, A. T., Leprevost, F. V., Avtonomov, D. M., Mellacheruvu, D. & Nesvizhskii, A. I. MSFragger: ultrafast and comprehensive peptide identification in mass spectrometry-based proteomics. *Nature methods* **14**, 513-520 (2017). <https://doi.org:10.1038/nmeth.4256>

20 Polasky, D. A., Yu, F., Teo, G. C. & Nesvizhskii, A. I. Fast and comprehensive N- and O-glycoproteomics analysis with MSFragger-Glyco. *Nature methods* **17**, 1125-1132 (2020). <https://doi.org:10.1038/s41592-020-0967-9>

21 Hayes, A. J., Lewis, J. M., Davies, M. R. & Scott, N. E. Burkholderia PglL enzymes are Serine preferring oligosaccharyltransferases which target conserved proteins across the Burkholderia genus. *Commun Biol* **4**, 1045 (2021). <https://doi.org:10.1038/s42003-021-02588-y>

22 Holden, M. T. *et al.* The genome of Burkholderia cenocepacia J2315, an epidemic pathogen of cystic fibrosis patients. *Journal of bacteriology* **191**, 261-277 (2009). <https://doi.org:10.1128/JB.01230-08>

23 Wickham, H. *ggplot2: Elegant Graphics for Data Analysis*. (Springer-Verlag New York, 2016).

24 Brademan, D. R., Riley, N. M., Kwiecien, N. W. & Coon, J. J. Interactive Peptide Spectral Annotator: A Versatile Web-based Tool for Proteomic Applications. *Molecular & cellular proteomics : MCP* **18**, S193-S201 (2019). <https://doi.org:10.1074/mcp.TIR118.001209>

25 Varga, J. J. *et al.* Draft Genome Sequences of Burkholderia cenocepacia ET12 Lineage Strains K56-2 and BC7. *Genome Announc* **1** (2013). <https://doi.org:10.1128/genomeA.00841-13>

26 Cox, J. *et al.* Accurate proteome-wide label-free quantification by delayed normalization and maximal peptide ratio extraction, termed MaxLFQ. *Molecular & cellular proteomics : MCP* **13**, 2513-2526 (2014). <https://doi.org:10.1074/mcp.M113.031591>

27 Tyanova, S. *et al.* The Perseus computational platform for comprehensive analysis of (prote)omics data. *Nature methods* **13**, 731-740 (2016). <https://doi.org:10.1038/nmeth.3901>

28 Sass, A., Everaert, A., Van Acker, H., Van den Driessche, F. & Coenye, T. Targeting the Nonmevalonate Pathway in Burkholderia cenocepacia Increases Susceptibility to Certain beta-Lactam Antibiotics. *Antimicrobial agents and chemotherapy* **62** (2018). <https://doi.org:10.1128/AAC.02607-17>
